# An integrated experimental and computational pipeline for crystallographic fragment screening of membrane protein in the lipid cubic phase

**DOI:** 10.1038/s42004-026-02059-7

**Published:** 2026-05-13

**Authors:** Chia-Ying Huang, Robert Cheng, Alexander Metz, Denis Bucher, Fabio Andres, Arianna Bacchin, Hannah Glover, Christoph P. Sager, Meitian Wang, Michel O. Steinmetz, Michael Hennig, May Sharpe

**Affiliations:** 1 PSI Center for Photon Sciences, Villigen PSI, Switzerland; 2 leadXpro AG, Park Innovaare, Villigen, Switzerland; 3 PSI Center for Life Sciences, Villigen PSI, Switzerland; 4 Biozentrum, University of Basel, Basel, Switzerland; 5 Present Address: UCB, Braine-l’Alleud, Wallonische Region, Belgien

**Keywords:** X-ray crystallography, High-throughput screening, Membrane lipids, G protein-coupled receptors

## Abstract

X-ray crystallographic fragment screening is a powerful strategy in modern drug discovery, enabling the identification of small-molecule starting points for rational hit-to-lead optimization. While highly effective for soluble proteins, its application to membrane proteins remains challenging due to low expression yields, high hydrophobicity, and the complexities of crystallization—particularly when using lipid cubic phase (LCP), which is often essential for high-resolution structural studies of targets like G-protein-coupled receptors (GPCRs). In this study, we present a methodology that integrates high-throughput X-ray crystallography with computational modeling and complementary biophysical validation to overcome these barriers. Using a thermostabilized human adenosine A_2A_ receptor crystallized in LCP as a test system, we screened 568 fragments and identified 23 initial hits. The work represents the first large-scale fragment screening effort targeting crystals of a membrane protein grown in LCP. Structure-guided virtual screening of these hits led to the design of 109 follow-up compounds, of which 56 yielded crystal structures. Of these, 19 were additionally confirmed to bind by grating-coupled interferometry (GCI), providing complementary biophysical validation. Our results demonstrated the feasibility and effectiveness of this integrated approach for fragment-based drug discovery on membrane proteins crystallized in LCP. Moreover, the detection of ligands at a previously uncharacterized intracellular pocket in a GPCR highlights the potential of this strategy to accelerate the discovery of therapeutically relevant compounds for challenging drug targets.

## Abstract (web summary)

Fragment screening by X-ray crystallography is challenging for membrane proteins, especially when crystals are grown in lipid cubic phase (LCP). Here, the authors establish a large-scale fragment screening approach integrating high-throughput LCP crystallography with computational and biophysical methods to identify ligands for adenosine A_2A_ receptor.

## Introduction

Structure-based drug design (SBDD) has long driven drug discovery, leveraging structural insights from X-ray crystallography, cryo-EM, and NMR to guide compound optimization^1,2^. Modern high-throughput synchrotron macromolecular crystallography has enabled crystallographic fragment-based drug discovery (xFBDD), in which low-molecular-weight fragments (≤ 300 Da) serve as starting points that can be elaborated into potent drug leads^3^. Particularly, xFBDD has shown success across diverse targets, including COVID-19-related proteins^4–9^.

SBDD and xFBDD have typically focused on soluble proteins, which are easier to express, purify, and crystallize. Membrane proteins (MPs), despite being major therapeutic targets, remain challenging due to low expression, instability, and hydrophobicity. Among MPs, G protein–coupled receptors (GPCRs) are of particular interest because of their key physiological roles and are targeted by many FDA-approved drugs, with growing interest in allosteric modulators^10–12^. Although FBDD has been applied to GPCRs using affinity-based methods or free energy simulations based on existing X-ray structures^13,14^, X-ray crystallography remains the most direct and informative method. Lipid cubic phase (LCP) crystallization was developed for integral MPs, especially GPCRs, as it provides a membrane-mimicking environment^15–18^. However, LCP crystallization poses significant challenges for high-throughput fragment screening. Crystal growth in fragile glass sandwich plates is low-throughput and labor-intensive, and handling or soaking crystals is technically demanding^19–22^. Additionally, fragment diffusion in LCP is slow, and the use of polar co-solvents like dimethylsulfoxide (DMSO) can destabilize the mesophase^23^. Furthermore, heterogeneity and low crystal yield constrain large-scale compound screening.

To address these challenges, several methods have been developed, including in situ thin-film soaking^24–27^, syringe-based crystal growth for XFEL^28^, and soaking strategies compatible with LCP protocols. While effective, these remain labour-intensive. More recently, Healey’s group^29^ introduced an automated platform using CrystalDirect (CD) plates, and applied it to soaking of LCP crystals, including direct LCP soaking of targets such as ACER3 and ADIPOR2. In addition, this platform is integrated with a web-based Crystallographic Information Management System (CRISM) for crystal growth, harvesting, and data collection.

Here, we present a hybrid LCP crystallographic fragment screening (LCP-CFS) workflow, which enables large-scale GPCR fragment screening. This integrates LCP crystallization, virtual screening, and biophysical assays. Using a thermostabilized human adenosine A_2A_ receptor^30^, a member of the class A GPCRs—integral MPs characterized by seven transmembrane helices that mediate signal transduction through allosteric interactions between extracellular and intracellular domains^31,32^. We designed the LCP-CFS method to utilize the Fast Fragment and Compound (FFCS) crystallographic screening pipeline at the Swiss Light Source (SLS)^33,34^, combining syringe-based LCP crystal growth with automated sample handling by robot. LCP samples were transferred into vapor diffusion sitting drop plates, enabling efficient and reproducible high-throughput fragment soaking. We initially selected 568 fragments (Supplementary Data 1) targeting the orthosteric site for X-ray crystallography. As expected, 3 fragments bound to the orthosteric site. Unexpectedly, 20 fragments were found to engage an intracellular, a funnel-like pocket on the intracellular side of the receptor. Given the novelty of this site, we shifted our focus and performed a second round of virtual screening specifically targeting it. These hits that bind on the intracellular side of the receptor guided virtual screening, yielding 109 follow-up compounds, 56 of which showed binding in crystal structures. 19 of them were additionally confirmed by grating-coupled interferometry (GCI). Together with our LCP-CFS workflow, this enables efficient ligand discovery for GPCRs and potentially other challenging MPs.

## Results

### Development of a method for lipid cubic phase crystallographic fragment screening (LCP-CFS)

**Fig. 1:**
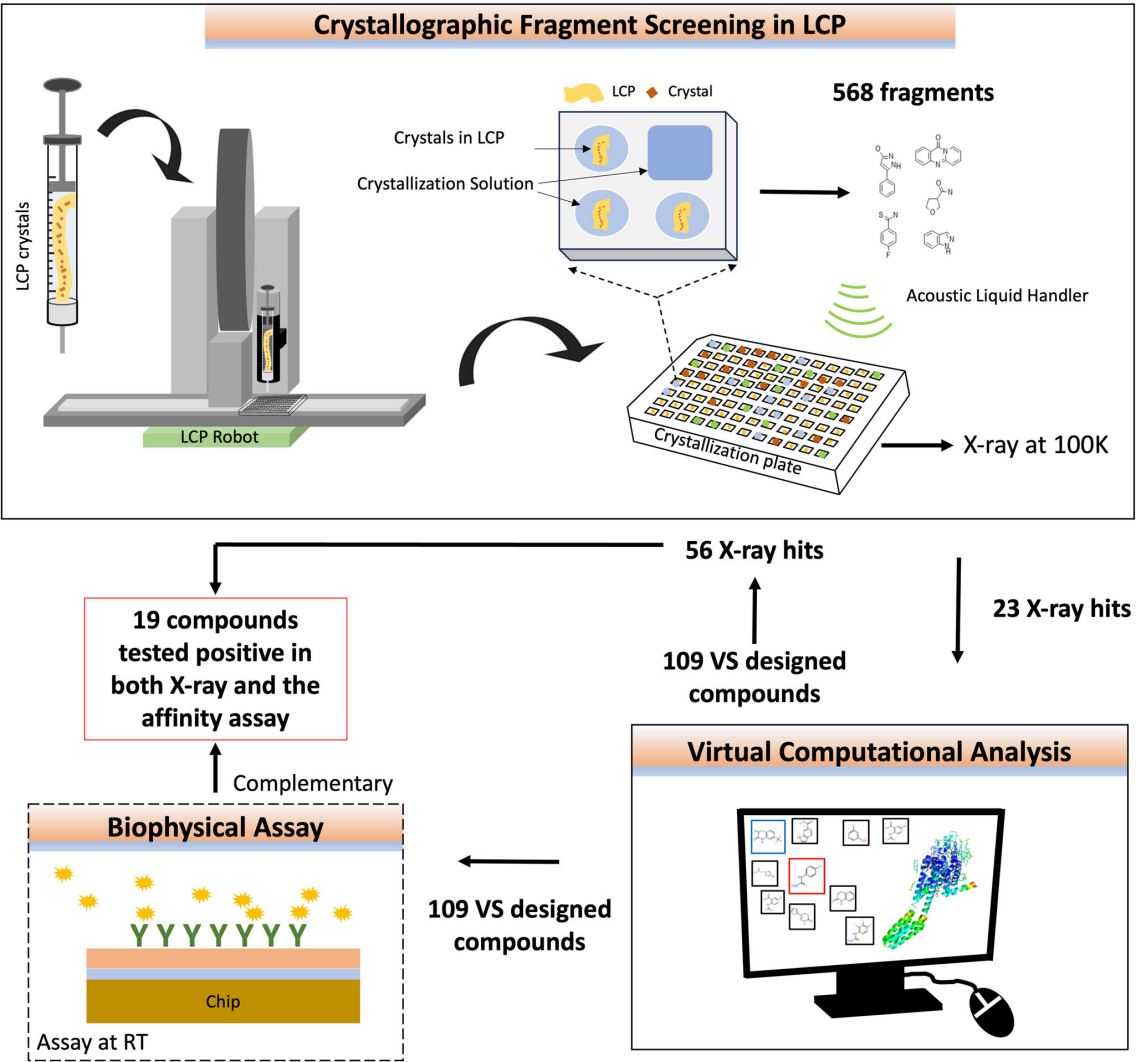
Scheme of the LCP-CFS pipeline developed in this study. The LCP sample is initially prepared in a glass syringe. Once crystals are observed, the crystallization solution is carefully removed, and the crystal-laden LCP sample is harvested. The sample is then loaded onto the LCP robot, which transfers it to a crystallization plate. This crystallization plate, containing the LCP sample in each well, can then be directly used in the FFCS pipeline developed at the SLS. Crystallographic hits are subsequently analyzed through virtual computational analysis to design potential fragment binders. These fragments are then validated for X-ray crystallography studies and can optionally be supported by additional biophysical assays, such as GCI, which may provide valuable complementary information. VS refers to virtual screening, and RT refers to room temperature.

The tsA_2A_R (human thermostabilized adenosine A_2A_ receptor with A277S revert mutation, A_2A_R-StaR2-bRIL-A277S^30^, hereafter referred to as tsA_2A_R, crystals were grown in the presence of theophylline, details in “Material and Method” section) crystal-laden LCP produced in a syringe was harvested and then transferred to a vapor diffusion sitting drop crystallization plate (SwissCI-3-lens UVXPO plates) using the Mosquito LCP robot. Crystallization buffer without theophylline was added to each well to prevent dehydration (Figs. 1 and S1). LCP crystals remained stable and birefringent for at least 24 h under these conditions.

DMSO is usually necessary to obtain high-enough fragment concentrations to achieve significant occupancy for weaker-binding fragments, but it may also affect the integrity of the LCP matrix^20,23^. To address this, we systematically optimized the DMSO concentration and soaking duration of compounds in tsA_2A_R crystal-laden LCP. Here, we used ZM241385^35–37^ as a positive control, a ligand known to bind at A_2A_R’s orthosteric site. At 10% DMSO (10 mM ZM241385), the LCP showed a phase change, while it remained intact at lower DMSO concentrations after 1.5 h of soaking (Fig. S2). The LCP in all conditions transitioned to a liquid-like sponge phase after 3 h of soaking and maintained this phase even after 21 h of soaking (Fig. S2). tsA_2A_R crystals in LCP treated with 1%, 5%, and 10% DMSO exhibited diffraction quality ranging from 2.4 to 2.6 Å in the highest resolution shell with average I/σ values of 0.8 and CC_1/2_ values of 0.34 (Fig. S3 and Supplementary Data 2). The resulting electron density maps revealed a clear density corresponding to ZM241385, demonstrating efficient soaking (5–10% DMSO, 5–10 mM ZM241385) and ligand binding (Fig. S4). We found that short soaking times (3 h) with 5–10% DMSO preserved both soaking efficiency and crystal diffraction quality. Although 10% DMSO with 10 mM ZM241385 induced an LCP phase change, it didn’t compromise the crystal quality. These data show that the soaking conditions of 3 h with 5 or 10 mM ligand (corresponding to 5 and 10% DMSO) yield comparable statistics. Given the low affinity of the fragments, we selected 10 mM ligand in 10% DMSO as the optimal condition for fragment soaking (Fig. S4). For longer soaks (21 h), ZM241385 bound effectively at a concentration of 1 mM with as little as 1% DMSO (Fig. S4f). For the tsA_2A_R-LCP sample, soaking in 15 and 20% DMSO was also performed. However, the high DMSO concentration reduced the resolution of the tsA_2A_R crystals to ~3.5 Å.

### Application of LCP-CFS to a GPCR

Using the LCP-CFS described here, we conducted a crystallographic fragment screening campaign targeting tsA_2A_R. We initially selected 568 fragments from our in-house fragment libraries (2500 Maybridge Ro3 Library (ThermoFisher) and a 1056 Ro3 selection provided by Idorsia Pharmaceuticals) for soaking. A total of 498 X-ray datasets were successfully collected from 568 tsA_2A_R-fragment-soaked crystals. For those fragments without a successful dataset, two datasets had resolutions worse than 4 Å and the remaining 68 fragments-soaked tsA_2A_R crystals either dissolved during the soaking or didn’t show any X-ray diffraction. The 498 useful datasets exhibited an average resolution of approximately 2.44 Å and achieved average I/σ values of 1.45 and CC_1/2_ values of 0.40 in the highest resolution shell (Fig. S5 and Supplementary Data 3). After molecular replacement, we performed a PanDDA analysis to identify bound fragments. To ensure the accuracy of PanDDA event detection, all datasets with R_free_ values greater than 40% were excluded. The highest-resolution dataset, F101 (1.98 Å) was selected as the reference for B-factor calculation. The analysis included datasets with resolutions ranging from 2.13 to 3.78 Å. High-confidence binding events were identified by applying filters of peak Z-score (z_peak) > 3 and event map volume (lig_volume) > ~50 Å³.

However, some partially bound 9.9 MAG compromised the identification of true fragment hits from false events in PanDDA. To mitigate this, we conducted a manual inspection to exclude hydrophobic regions near the bilayer interface where MAG typically binds (Fig. S6). Through this combined approach, our initial inspection of the electron density maps revealed 3 fragments bound in the orthosteric binding site (Fig. S7a), displacing the theophylline that was essential for crystallization, and 20 fragments bound to a distinct intracellular site (Fig. S7b, c).

### Insights into fragments binding at the extracellular site of tsA_2A_R

**Fig. 2:**
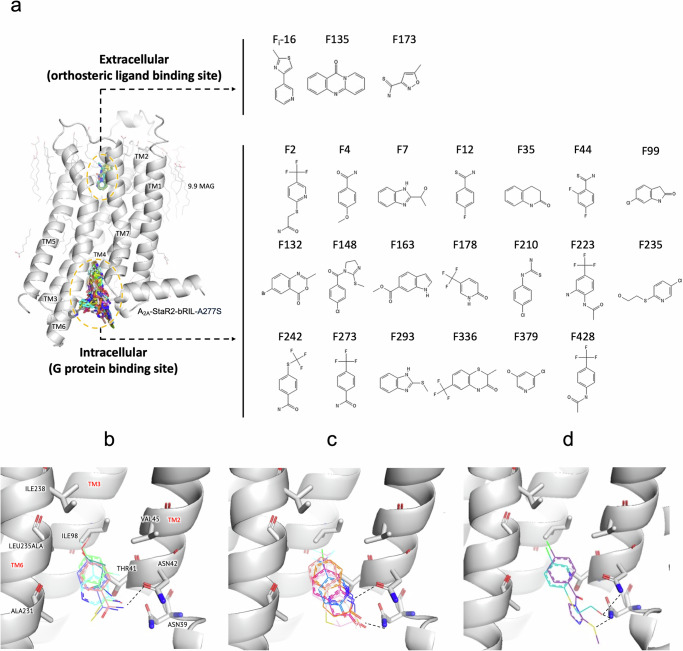
Overall structure of tsA_2A_R with initial fragment hits and their binding mode. **a** The protein is shown in cartoon representation with labels TM1 to TM7, and the surrounding 9.9 MAGs are shown in stick representation; both are colored in gray. Initial fragment hits identified in the current study are displayed in stick representation and colored variably. Three major binding modes were observed from the initial hits with the residues ASN39 and ASN42 of tsA_2A_R. **b** F4, F12, F44, F242, F273, and F379. **c** F7, F35, F99, F178, F293, and F336. **d** F148 and F235.

Among the 23 hits, the three fragments binding to the extracellular (orthosteric) site form hydrogen bonds with ASN253, a conserved residue critical for ligand binding. F_i_16 (fragments designated as “F” followed by a number) contains pyridine linked to a thiazole ring, mimicking the hydrogen bonding pattern formed by adenine in other A_2A_R ligands (Figs. 2 and S7a). F135 is a quinazolinone derivative with a larger aromatic system, forming a hydrogen bond with ASN253. F173, smaller in size, has a methyl-substituted isoxazole group and a thioamide moiety, also forms a hydrogen bond with ASN253. These findings highlight the ability of LCP-CFS to identify fragments in the tsA_2A_R orthosteric site that form key interactions with known residues and are able to displace the theophylline.

### Insights into fragments binding at the intracellular site of tsA_2A_R

Although all 568 fragments were selected to target the orthosteric site, X-ray crystallography revealed that 20 fragments unexpectedly engaged an intracellular, funnel-like pocket. Given its novelty, we shifted focus to specifically target this site.

The intracellular site is a funnel-shaped cavity lined by transmembrane helices TM2, TM3, TM6, and TM7 as well as helix 8 and the intracellular loop ICL1 (Fig. S8a). It is characterized by two main features: a distinct hydrophobic region at the narrow apex of the funnel and a hydrophilic interaction motif along the tunnel wall, formed by the side chain amide groups of ASN39 and ASN42, positioned side by side at the transition from ICL1 and TM2 (Fig. S8a). Initially, upon inspection of the electron density maps of the tsA_2A_R–fragment structures, we contoured the sigma cut-off of *2Fo–Fc* and *Fo–Fc* maps at 1.0 and 3.0, respectively, and clearly identified three fragments (F132, F210, and F428) bound at the intracellular site. The three identified fragments are bound with their hydrophobic, halogen-bearing portion in the apparent hydrophobic hot spot at the apex, with their hydrophilic features forming direct interaction to the two asparagine side chains in the tunnel wall (Fig. S7b).

It must be noted that this intracellular site appears to be further stabilized and/or deepened by the alterations introduced to tsA_2A_R. In particular, the hydrophobic portion of the cavity is located close to the stabilizing LEU235ALA_6.37_ mutation (Fig. S8), which along with the other stabilizing mutations (A54L, T88A, R107A, K122A, N154A, L202A, L235A, and V239A), affects the shape of the cavity. When superimposing a tsA_2A_R structure with the wild-type (e.g., PDBs 8GNG, 7PYR, and 4EIY), the LEU235 side chain reaches into the hydrophobic hot spot and overlaps partially with most of the bound fragments (Fig. S8b, c). The only exception is fragment F132, which shows the closest distances to the wild-type A_2A_R structure (PDB 8GNG) at 1.2 Å and to other wild-type A_2A_R structures (PDBs 7PYR and 4EIY) at 1.5 Å (Fig. S8b). Although we can only speculate to what degree these observations affect the physiological relevance of this intracellular binding site, we consider those fragments a proof of concept for the feasibility of crystallographic fragment screening against membrane protein crystals grown in LCP. Moreover, we think that it would be possible to develop those fragments into more potent binders for A_2A_R and subsequently remove those parts overlapping with LEU235.

Intrigued by the fragment hits observed at the intracellular site, we decided to investigate further to identify any additional fragment hits that may have been missed in the initial inspection. Therefore, we systematically visualize and inspect the electron density in this region using a lower sigma cut-off of 0.7–0.8 and 2.5–2.8 for the 2*F*_*o*_*–F*_*c*_ and *F*_*o*_*–F*_*c*_ maps, respectively. This revealed a substantial number of additional hits (Fig. S7c and Supplementary Data 3), along with other densities that are less certain to represent fragments but cannot be definitively excluded as such. We found an additional 17 fragments bound at the intracellular sites.

These 17 fragments can be categorized based on shared structural motifs that support intracellular binding to tsA_2A_R, and they mainly show three binding modes (Figs. 2b–d and S7c). A prominent group (e.g., F2, F223, F242, and F273) contains trifluoromethyl or fluorinated phenyl rings, enhancing hydrophobic interactions and potentially contributing to halogen bonding. Two fragments (F12 and F44) feature thiourea moieties, providing versatile hydrogen bonding capabilities. Fragments such as F7, F35, F99, F163, F293, and F336 share fused aromatic or heteroaromatic cores (e.g., indazole, isoindolinone, benzimidazole), supporting hydrophobic interactions and rigid spatial orientation. Amide-containing fragments like F4 and F223 offer synthetic flexibility and hydrogen bond donors/acceptors. Halogenated aromatics (F99, F235, and F379) enhance lipophilicity and non-covalent binding. Importantly, F148 combines a thiomethyl-imidazolidinone ring with a chlorinated phenyl, offering both polar contacts and hydrophobic anchoring. These diverse chemical features highlight fragment versatility for structure-guided development of intracellularly active A_2A_R modulators (Fig. S7). Notably, these fragments form hydrogen bonds with conserved polar residues ASN39 and ASN42, as observed in the electron density maps (Fig. S7c), suggesting that these molecules engage with a defined polar sub-pocket within the receptor.

### Follow-up rational compound design

Most of the 20 fragment hits occupy the hydrophobic hot spot and form interactions with the side chains of ASN39 and ASN42 (Fig. S7b, c). Inspecting these poses, we saw opportunities to further develop these fragments to target unoccupied regions within the cavity, forming additional interactions with ASN39, ASN42, and other nearby residues (Fig. S8a). Additionally, several fragments share common scaffolds (Fig. S7b, c), providing opportunities for merging based on their maximum common substructures (MCS).

We took this opportunity to test whether our crystallographic screening protocol could also accommodate compounds larger than fragments, to confirm the method’s suitability to support all stages of a structure-guided FBDD campaign. For this purpose, we conducted a structure-based virtual screening to identify optimized follow-up compounds based on the fragment hits observed at the intracellular site of tsA_2A_R (Supplementary Data 4 and 5). Specifically, we developed a docking workflow that tethers the MCS of fragment analogs to the crystallographically observed fragment pose, fixing their rotation and translation but allowing flexibility through rotatable dihedral bonds to explore the cavity (see “Materials and Methods”). Fragment analogs were sourced from vendor catalogs. To maintain timely progress while acknowledging existing uncertainties, we proceeded with the virtual screening using structures that were not yet fully refined, including a subset of promising yet less certain events.

Notably, a few selected follow-up candidates were manually designed based on the observed pharmacophores and interaction patterns. In particular, FU_132,428_-27 was created by merging the hydrophilic substituents of the overlapping ring systems, altering the N-acetamide to an urea to possibly form an additional hydrogen bond. Also, FU_428_-8 and FU_428_-36 were manually designed by merging F428 with a peptide loop from an antibody Fab fragment bound to A_2A_R (PDB: 8GNG). GLY105 of this loop aligned well with the amide of F428, allowing the fragment’s hydrophobic portion to extend into the hydrophobic hotspot. The closest commercially available analogs of this merged model were then selected. This approach ultimately led to the selection and acquisition of 109 follow-up candidates (Supplementary Data 5) focusing on the formation of additional interactions and aiming to maximize diversity in chemotypes and predicted interaction patterns.

### Hits identified through X-ray crystallography

Screening these 109 follow-up candidates with the same protocol used for the initial fragments revealed 56 binders, resulting in an approximate 51% hit rate (Figs. S9 and 10). The 109 datasets exhibited an average resolution of 2.2 Å in the highest resolution shell, with average I/σ values of 1.30 and CC_1/2_ values of 0.5 (Fig. S9 and Supplementary Data 4).

From those 56 fragment hits, significant structural convergence and chemical diversity were revealed, offering valuable insights into conserved binding motifs and fragment optimization strategies. Prominent among these were amide-bearing aryl and heteroaryl compounds (e.g., FU_132_-3, FU1_32,428_-27, FU_163_-67, FU_293_-69, and FU_163_-75) and fused lactams (FU_35_-2, FU_12_-19, FU_336_-22, FU_336_-32, FU_336_-34), suggesting a preference for planar, hydrogen bond-capable scaffolds. Other recurrent chemotypes included aromatic sulfonamides (FU_132_-4), benzothiazolone (FU_223_-7), N-phenyl-tetrazol-5-amine (FU_35_-5), and phenylpyrazole (FU_4_-6). These motifs combine hydrophobic and aromatic elements found in the originating fragment hits with additional hydrogen bonding motifs that often interact with the side chain amide groups of ASN39 and ASN42, as intended by our design. Notably, hydrophobic substituents such as bromine (e.g., FU_132_-3), chlorine (e.g., FU_223_-7), fluorine (e.g., FU_44_-18), trifluoromethyl (e.g., FU_2_-57, methoxy (e.g., FU_7_-1), and trifluoromethoxy (FU_242_-98) bound consistently in the hydrophobic hotspot. Notably, variations in the size of these non-cyclic hydrophobic substituents are coupled with shifts in the position and orientation of the parent ring system. Rings lacking such substituents often extend deeper into the hydrophobic hot spot, a behavior further influenced by the ligand’s other interactions.

As further examples, several members of the FU_336_ series feature a 2H-1,4-benzothiazin-3(4H)-one scaffold with the aforementioned hydrophobic and hydrogen bonding substitutions, underscoring their scaffold-hopping potential in this site (Fig. S10). Compounds such as FU_99_-31, FU_99_-89, and FU_99_-91 contained a thiobenzimidazole motif that forms a hydrogen bond to ASN42 while directing their S-linked substituent towards ASN39 and beyond. Overall, these ligands displayed favorable complementarity to the intracellular binding site, functional group diversity, and synthetic tractability. Despite the high hit rate, numerous variations of the original fragment hits did not lead to an observed binding event or yielded unanticipated binding poses that seem less promising for further design. These findings narrow down the chemical space and exclude certain chemotypes from consideration, guiding the way for further design. Collectively, the scaffold distribution in the observed hits reflects a balance of aromaticity, heterocyclic content, and key pharmacophores, providing a strong foundation for further structure-based molecular design efforts.

### GCI analysis of hits identified through structure-based virtual screening

The 109 follow-up candidates were additionally analyzed for direct interaction with A_2A_R using a surface-based biosensor, GCI, with the waveRAPID method^38^, providing complementary biophysical validation. For this, the 109 compounds were injected into parallel flow cell sensors containing immobilized wtA_2A_R, tsA_2A_R, or a non-related GPCR (tsGPR17), or no immobilized protein. Adenosine and NECA were used as functional controls, showing the expected binding response for apo A_2A_R (Fig. S11). In contrast, no binding was observed when the orthosteric site was blocked with ZM241385 (Fig. S11). Therefore, to specifically probe the intracellular site, ZM241385 was included at saturating concentration in all GCI experiments with the 109 follow-up compounds.

The waveRAPID method based on pulsed injections with increasing duration was used (see “Materials and Methods”), which in principle allows for the acquisition of kinetic and affinity data in addition to target engagement^38^. However, reliable and precise kinetic information remains challenging to obtain for MPs in detergent micelles, especially for weak interactions. This is due to signal dynamics and artifacts related to non-specific adsorption of fragments to detergent micelles, resulting in lower confidence fits and related kinetic parameters. Here, based on the observed quality of the acquired sensorgrams—such as low amplitudes, distortions, low affinities, and the goodness of the fits using a 1:1 interaction model—only binary information about target engagement could be extracted from these datasets. The estimated kinetic rate constants, *K*_D_, and *R*_*max*_ values shown on the plots are therefore not considered precise, and no interpretations are drawn from them. All identified interactions are assumed to have weak affinity with *K*_D_ > 50 μM; a range where precise characterization of kinetics and affinity for detergent-solubilized MPs with a surface biosensor  is highly challenging. Representative GCI waveRAPID assay and multi-cycle kinetics (MCK) sensorgrams are shown in Figs. S12 and S13, respectively.

**Fig. 3:**
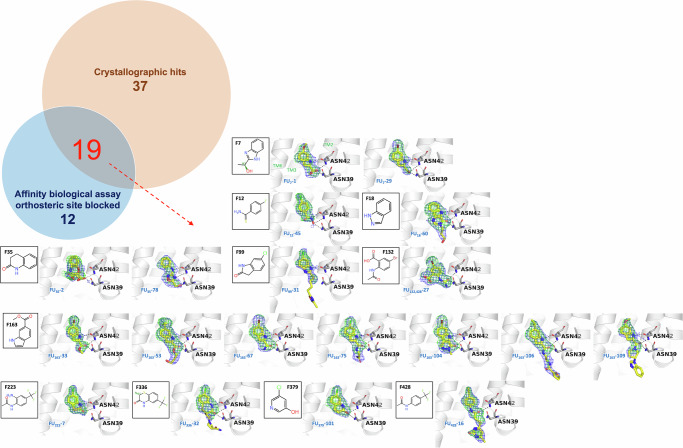
Follow-up compounds/fragments targeting the intracellular binding site of tsA_2A_R validated by X-ray crystallography and affinity biological assays. The lead fragments bound to the intracellular site of tsA_2A_R, identified from the initial hits in the LCP-CFS screen, are shown as chemical structures in squares. The following atoms are shown in the chemical structures: nitrogen and hydrogen are colored blue, oxygen red, bromine brown, fluorine light green, chlorine fluorescent green, and sulfur yellow-brown. Carbon atoms are shown as black lines. The structure of tsA_2A_R around the intracellular pocket is shown in cartoon representation in gray, with the intracellular side oriented face-down. The helices of tsA_2A_R are depicted in the first figure (FU_7_-1). The identified fragments, along with residues ASN39 and ASN42, are shown in stick representation and highlighted in yellow and gray, respectively. *F*_*o*_−*F*_*c*_ electron density maps, calculated using a model based on PDB 5IU4 with the ligand excluded, are displayed as a green mesh contoured at the 2.5 σ level. Hydrogen bonds within a distance of 3.5 Å are indicated by dashed lines. The 2*F*_*o*_−*F*_*c*_ electron density maps, calculated using the model with ligand, are displayed as a green mesh contoured at the 1.0 σ level.

Out of the 109 tested compounds, the GCI waveRAPID assay revealed 31 fragments interacting with ZM241385-saturated A_2A_R, of which 19 exhibited density in the X-ray screen (Figs. 3 and S14 and Supplementary Data 6). Although 12 of the 31 fragments exhibited positive binding signals in the GCI waveRAPID assay, no corresponding electron density was observed in the X-ray crystallographic data (Supplementary Data 7). Almost all 19 identified interactors could be confirmed to bind to both wtA_2A_R and tsA_2A_R. Two compounds (FU_336_-32 and FU-78) not detected as interactors with wtA_2A_R in the initial screen still showed binding to wtA_2A_R in the hit validation assay applying prolonged association times (Supplementary Data 6). Selected fragments were additionally subjected to classical MCK analysis and could be further confirmed as interactors with A_2A_R (Supplementary Data 6).

## Discussion

In this study, we developed an innovative lipid cubic phase-based crystallographic fragment screening (LCP-CFS) pipeline that seamlessly integrates syringe-grown LCP crystals with conventional vapor-diffusion-based crystallographic screening methods. Using a thermostabilized, human adenosine receptor as a test system, the pipeline achieved a success rate of 4% (23 out of 568 fragments), identifying fragments in two distinct binding pockets of the GPCR. Subsequent virtual screening enabled a focused follow-up, yielding a 51% success rate (56 out of 109 ligands), with 19 ligands exhibiting both significant GCI binding affinity and distinct ligand poses.

Crystal harvesting from LCP, particularly on glass plates, remains challenging, but innovations such as Laminex Film covers^21^ and affinity-based co-crystallization^20^ have improved throughput despite requiring labor-intensive well cutting. To overcome these limitations, we used a 96-well sitting-drop plate compatible with the Echo system and our FFCS pipeline for direct fragment dispensing, enabling high-throughput crystallographic screening. This setup, unlike traditional glass sandwich plates, allows more efficient LCP crystal harvesting using a Crystal Shifter (Oxford Lab Technologies)^39^ equipped with a cross-polarizer, significantly improving visibility and handling. With this setup, we harvested 100 samples within 1.5 h. On average, fewer than five crystals were harvested per mesh loop and one per regular loop. Automated X-ray diffraction data collection enabled identification of individual crystals in multicrystal loops and avoided overlapping diffraction.

Phase transitions of the lipidic cubic phase (LCP) have the potential to negatively impact certain LCP-grown crystals. In the present study, soaking conditions were optimized to minimize adverse effects, including adjustment of DMSO concentration and incubation time. Notably, tsA_2A_R crystals exhibited spontaneous transformation of LCP into the sponge phase after several hours of incubation, even in the absence of DMSO or ligand. Despite this transition, diffraction quality remained unchanged, consistent with previous reports demonstrating that tsA_2A_R crystals tolerate such phase changes. The enlarged aqueous channels characteristic of the sponge phase may, in fact, facilitate fragment diffusion and thereby enhance soaking efficiency in this system^18^. However, this tolerance is unlikely to be universal. For other MPs, particularly those sensitive to dehydration or phase instability, phase transitions may compromise crystal integrity and thus represent a methodological limitation. Careful control of humidity and minimization of transfer time during handling of LCP from syringe to plate may mitigate this risk. In addition, higher fragment concentrations could accelerate diffusion, enabling soaking to be completed prior to substantial phase transition.

To establish the robustness of the LCP-CFS soaking approach, the high-affinity antagonist ZM241385 was initially employed to validate ligand uptake and define baseline soaking parameters. Its reproducible binding under the selected conditions confirmed effective ligand incorporation and enabled the identification of parameters compatible with fragment screening. These optimized conditions subsequently yielded fragment hits, supporting the suitability of the method for weak binders. Although a low-affinity antagonist (Ki > 1 µM) would more closely approximate fragment-like behavior, practical constraints, including limited availability of suitable weak ligands during method development, necessitated the use of ZM241385 for initial validation.

The initial fragment hits effectively mapped the chemical space of A_2A_R’s intracellular binding site, revealing key interaction hotspots such as ASN39 and ASN42. By combining tethered docking, anchoring fragment analogs to observed fragment poses, we obtained a ranked list of predicted binding poses for follow-up candidates of each fragment hit. We then filtered the list for unique chemotypes and prioritized diverse follow-up compounds, each designed to test a distinct hypothesis with the potential to enhance binding. Rather than focusing solely on hit rate, potency, or immediate drug-likeness, our selection strategy also emphasized maximizing chemotype diversity among follow-up compounds to identify the most promising scaffolds for further development. In cases where the exact compound was not commercially available, we acquired the closest commercially available analogs that test the same hypothesis.

While our virtual screening strategy, based on freely available software, successfully enriched potent follow-up hits, its performance could likely be further improved by integrating advanced computer aided drug design techniques such as free energy perturbation (FEP), currently the gold standard for computational prediction of ligand binding affinities^40^. FEP is most effective for congeneric series of ligands, whereas fragment screening aims to explore diverse chemical starting points, which limits the efficiency of relative FEP and instead requires absolute binding free energy (AB-FEP) calculations, an approach that is more computationally demanding and often less accurate^41^. Nevertheless, the cost and effort of FEP calculations are broadly comparable to acquiring on stock follow-up compounds and conducting an LCP-CFS campaign, and an integrated computational, experimental workflow would likely be the most effective strategy. Nonetheless, our approach enabled a rapid progression to the second round of screening by X-ray crystallography, accelerating the evaluation of 109 follow-up compounds and streamlining the fragment optimization process.

Based on GCI assays and X-ray crystallography, 19 fragments showed unambiguous evidence of binding in both methods (Fig. 3 and Supplementary Data 6). While GCI can resolve affinity and kinetics for small molecules with molecular mass higher than 350 Da, fragment binding assessment is more challenging. Likely, data on fragments with better affinity and longer residence time are more reliable. In addition, GCI data can determine whether fragments bind at a site overlapping with ZM241385 (a ligand known to bind at A_2A_R’s orthosteric site, in current study) but do not provide detailed information about the exact binding location. In contrast, crystallography offers direct structural insight into the binding site and pose, which represents a key advantage for subsequent fragment elaboration. Notably, 37 fragments were identified only by X-ray and 12 only by GCI, which is not unexpected given the distinct experimental conditions of the two methods. Crystal soaking tolerates much higher fragment concentrations in different buffer and experimental conditions compared to GCI. Supporting this, previous studies have shown that over half of fragment hits observed by cryo-cooled X-ray crystallography fail to appear in biophysical assays^42^ or at room-temperature X-ray conditions^43–49^. Thus, discrepancies between the sets of binders identified by the two methods likely arise from differences in experimental conditions and from the inherent strengths and limitations of each technique. Accordingly, all hits identified by either method were considered potential candidates for follow-up studies. Nevertheless, the structural information obtained from crystallography provides a significant advantage for prioritizing and optimizing fragment hits. Together, the two approaches deliver complementary information on fragment binding, consistent with previous reports^50–54^.

Using the GPCRdb, we searched for compounds with more than 50% similarity to our compounds/fragments. This revealed that 42 of our 109 follow-up fragments have analogs previously tested against targets in the database, with similarity scores ranging from 50% to 100%, although these analogs do not necessarily show activity (Supplementary Data 5). Notably, FU_113_-13 is listed in the GPCRdb (ID 50365) and has been tested for activity against the human thyroid-stimulating hormone receptor (TSH receptor, a Class A Rhodopsin-like GPCR) with a potency of 39.8 μM listed in the ChEMBL database^55^. Among these 42 compounds/fragments with analogs listed in the GPCRdb, 21 were identified as X-ray hits in our study, 11 were GCI hits with the A_2A_R orthosteric site blocked, 6 were hits in both X-ray and GCI assays (with A_2A_R orthosteric site blocked). These findings suggest that our LCP-CFS method and design strategy can effectively identify small-molecule effectors of GPCR targets, as many follow-up fragments share similarity with previously tested compounds.

While the development of our fragment hits into lead or drug-like compounds is beyond the scope of this study, we highlight a few selected cases that exemplify the potential of this site (compare Figs. S7 and S10). FU_336_-32, with the highest fragment-based druglikeness value (*d* = 7.7) calculated using DataWarrior^56^ and FU_35_-78, which is well resolved and forms strong interactions, represent promising starting points for structure-guided design because they are well anchored in the binding pocket and present clear opportunities for further elaboration. FU_428_-8 and FU_428_-36 were manually designed by merging fragment F428 with a peptide loop from an antibody Fab fragment bound to A_2A_R (PDB: 8GNG), demonstrating how fragment screening can be combined with design inspiration from protein–protein interactions. FU_132,428_-27 exemplifies precise fragment merging, as it maintains the exact binding positions of its parent fragments F132 and F428 while uniting their interaction motifs. Collectively, these examples illustrate how our high-resolution LCP-CFS structures provide a strong foundation for chemically expanding these ligands through structurally enabled molecular design. While most fragments identified in this study bind to the hydrophobic hot spot at the apex of the intracellular funnel, a pocket potentially unique to the A_2A_R mutant and absent in the wild-type receptor, we have not yet explored the potential of these ligands to be converted into more potent wild-type A_2A_R binders by removing the moiety occupying this site.

Despite potential concerns regarding the physiological relevance of binding to an intracellular site influenced by mutations in the A_2A_R, the crystallographic identification and subsequent optimization of fragment binders reported in this study establishes a foundation for alternative strategies to identify and develop GPCR ligands and unlock new therapeutic opportunities. These examples underscore how our fragment-derived ligands exemplify the relevance of intracellular GPCR binding, while demonstrating that our LCP-CFS approach is broadly applicable to both intra- and extracellular GPCR sites and potentially to other membrane protein targets. Moreover, the method could also be tuned to specifically target allosteric site(s) if one had used a potent orthosteric compound during crystallization that could not be displaced with fragments during soaking.

Looking ahead, the broader applicability and future development of the LCP-CFS platform merit further application in drug design. Recent crystal structures have revealed a striking diversity of allosteric binding sites in GPCRs, including conserved intracellular pockets such as those observed in C–C Chemokine Receptor Type 2 (CCR2), C–C chemokine receptor type 9 (CCR9), and β₂-adrenergic receptor (β₂AR)^57^, which represent highly attractive pharmacological targets. Targeting these sites with small molecules is challenging, and LCP-CFS is well-suited to systematically explore their ligand ability and provide structural templates for fragment-based drug discovery. This raises the question of which GPCRs or other MPs already solved in LCP are most amenable to such studies, and conversely, which fragile targets may remain challenging for this approach. The success of LCP-CFS requires the availability of a large number of crystals diffracting to 2.5 Å or better. Moreover, while our fragment library was designed primarily for orthosteric binding, its unexpected success in identifying allosteric ligands highlights the adaptability of LCP-CFS and points to future opportunities to refine fragment selection toward intracellular or other allosteric sites, including tuning physicochemical properties relevant to membrane permeability. Notably, several of our hits contain charged groups, suggesting that membrane permeation is not an intrinsic limitation under LCP conditions, potentially facilitated by the composition and layered geometry of the LCP matrix. Finally, beyond structural applications, LCP-CFS could be leveraged to systematically investigate ligand engagement across intra- and extracellular GPCR sites, supporting small-molecule discovery for a wide range of membrane protein targets.

### Conclusions

In summary, we developed a high-throughput lipid cubic phase-crystallographic fragment screening (LCP-CFS) platform targeting a GPCR. We overcome technical challenges by method development and optimization of all the steps from crystallization to data analysis. Here, we performed the first large-scale (> 500 fragments) fragment screening in LCP-grown MPs. This approach yielded a 4% (23 out of 568 fragments) initial fragment hit rate and a significantly increased hit rate for follow-up compounds 51% (56 out of 109 ligands) identified through virtual screening. Among the X-ray hits, nineteen fragments also showed binding in GCI assays, highlighting their potential for intracellular targeting. Optimized soaking with 10% DMSO preserved crystal integrity while enhancing solubility of the compounds. Intracellular hits, forming key interactions with ASN39 and ASN42 of A_2A_R, represent promising scaffolds and explore a previously uncharacterized intracellular pocket that could be used for allosteric modulation of the activity of the receptor, providing synergistic activity effects with orthosteric ligands, selectivity for A_2A_R, and a new starting point for drug discovery efforts. Our LCP-CFS platform demonstrates the ability to apply fragment screening on membrane protein targets using X-ray crystallography and successfully discovered new chemical matter for both orthosteric and allosteric sites. High-throughput X-ray crystallography is a powerful and complementary approach to biophysical and cellular-based assays, enabling the identification of previously uncharacterized chemical hits. It readily tolerates higher compound concentrations that often interfere with other assay formats and delivers immediate structural insights that directly accelerate ligand optimization.

## Methods

### Protein purification and LCP preparation

For crystallization, a thermostabilized (ts) human adenosine A_2A_ receptor with A277S revert mutation^30^ (A_2A_R-StaR2-bRIL-A277S; hereafter referred to as tsA_2A_R) was used. Briefly, the construct contains eight mutations: A54L, T88A, R107A, K122A, N154A, L202A, L235A, and V239A. A FLAG tag is added at the N-terminus of A_2A_R. After K315, the remaining A_2A_R residues are replaced with 3× alanines and 10× histidines. Apocytochrome b562 RIL (bRIL) is inserted into the third intracellular loop (ICL3) between L208 and E219. tsA_2A_R was expressed and purified as described previously^58^ except no ligand was used in the purification. Briefly, protein was expressed in *Trichoplusia ni* Hi5 cells grown in Sf900 II medium at 27 °C. After infection with baculovirus (2.5% VOI), cells were incubated for 48 h before harvesting. Protein purification was performed at 4 °C using a Ni-NTA (nickel–nitrilotriacetic acid) Superflow cartridge (Qiagen) pre-equilibrated in 40 mM Tris (pH 7.5), 200 mM sodium chloride, and 0.15% (w/v) n-Decyl-β-maltoside. The column was washed with the same buffer containing 70 mM imidazole and eluted with 280 mM imidazole. The eluate was further purified by size exclusion chromatography on a Superdex 200 10/300 column (GE Healthcare) equilibrated in the same buffer. Purified A_2A_R was concentrated to 25–30 mg ml^−1^, aliquoted, snap-frozen in liquid nitrogen, and stored at −80 °C until use.

For GCI, tsA_2A_R and wild-type (wt) A_2A_R constructs (the wtA_2A_R construct is designed the same as tsA_2A_R, but no mutations are introduced) were purified using the same protocol as described above except LMNG was used throughout solubilization and purification (final LMNG/CHS concentration in the buffer: 0.005% (w/v) LMNG/0.0005% (w/v) CHS).

A_2A_R-laden mesophase was produced by homogenizing two volumes of protein solution at 25–30 mg ml^−1^ in buffer (40 mM Tris-HCl pH 7.5, 200 mM sodium chloride, 0.15% (w/v) n-Decyl-β-maltoside, 0.002% CHS, 5% glycerol) with three volumes of the monoacylglycerol (MAG) lipid monoolein (9.9 MAG) with 10% cholesterol in a coupled-syringe mixing device^21^ at 20 °C, as described previously^15^. Seven µl of protein-laden mesophase was injected into a 100 µl Hamilton syringe with 70 µl crystallization buffer (100mM sodium citrate pH 5.0, 50 mM sodium thiocyanate, 3% 2-Methyl-2,4-pentanediol (MPD) (v/v), 5 mM theophylline, 25–30% PEG 400) and stored at 20 °C. Crystals grew over 2–3 weeks and only harvested before use.

### Lipid cubic phase crystallographic fragment screening (LCP-CFS)

tsA_2A_R crystals were obtained after 14–21 days with ~50 µm at the longest dimension. Crystals were harvested from the syringe after removing the crystallization solution. One hundred nL of crystal-laden LCP was dispensed into the protein well of a SwissCI-3-lens crystallization (SWISSCI) plate using a Mosquito robotic dispenser (SPT Labtech) (Fig. 1) and immersed in 500–800 nL of crystallization solution without theophylline. Additionally, 30 µL of reservoir solution was added to the reservoir well to maintain vapor equilibrium and prevent dehydration of the crystallization drop after sealing. The reservior solution here consisted of 100 mM sodium citrate pH 5.0, 50 mM sodium thiocyanate, 30% (v/v) PEG 400, 3% (v/v) MPD.

To evaluate the impact of DMSO concentration on crystal quality and fragment binding, a strong binder of A_2A_R, ZM241385^35–37^ was prepared at 100 mM in 100% DMSO. This stock solution was then diluted to achieve final concentrations of 1% (v/v), 5% (v/v), and 10% (v/v) DMSO for soaking experiments with tsA_2A_R-laden LCP crystals using an Echo 550 acoustic liquid-handling robot (Labcyte). A soaking concentration of 10% (v/v) DMSO was selected, as X-ray diffraction data revealed no deterioration in quality at this concentration.

Solutions of 568 fragments at concentrations of 100 or 200 mM in DMSO were prepared in either Echo Qualified 384 low-dead-volume COC microplates (Beckman Coulter) or Echo Qualified 384-well polypropylene microplates (Beckman Coulter). These fragment solutions were then transferred to LCP crystals prepared in SwissCI-3-lens crystallization plates using the Echo 550 acoustic liquid-handling robot (Labcyte) to a final concentration of 10 or 20 mM (10% (v/v) final concentration of DMSO). Plates were then sealed and incubated at 20 °C. Fragment-soaked crystals were harvested after either 3 or 21 h, and a Crystal Shifter^®^ robot (Oxford Lab Technologies)^36^ was used to facilitate and record the harvesting process. Cross-polarization light was used to facilitate the observation of the LCP crystals during the harvesting. Crystals were carefully harvested using MiTeGen cryoloops or mesh loops and snap-cooled in liquid nitrogen without the addition of cryoprotectant. Samples were then stored in UniPucks for subsequent X-ray data collection.

### Initial fragment selection: virtual screening in the orthosteric site

Fragments from our two inhouse fragment fragment libraries, a 2500 Maybridge Ro3 Library (ThermoFisher) and a 1056 Ro3 selection provided by Idorsia Pharmaceuticals, were pre-screened using a pharmacophore approach (Phase), in the Schrödinger software. The pharmacophore model was designed to enrich ligands that bind to the orthosteric site by forming two hydrogen-bonds with the key residue, ASN253. The pharmacophore also included an aromatic pi–pi stacking interaction with PHE168. The top 1/3 of the fragments ranked by the pharmacophore score were then re-docked with Glide SP, followed by manual selection of the most interesting and original fragments (Supplementary Data 1).

### Automation data collection and structure determination

X-ray diffraction experiments were carried out on the X10SA-PXII protein crystallography beamline at the SLS, Villigen, Switzerland, and the European Synchrotron Radiation Facility (ESRF) ID30B, Grenoble, France. Data were collected at 100 K with a cryostream. Automatic data collection was conducted using the Smart Digital User^59^ developed at the SLS with crystal-rotation steps of 0.2° at a speed of 0.02 s per step using an EIGER2 16 M detector (DECTRIS) operated in continuous/shutterless data-collection mode. The beam transmission, flux, beam size, and total angular range per data set were 100%, ∼2.0 × 10^12^ photons s^−1^, 75 × 20 µm, and 180°, respectively. The estimated maximum X-ray dose was 12.06 MGy^60^. For the data collected at the ESRF ID30B, measurements were made using the MXPressE automation strategy with steps of 0.2° at a speed of 0.1 s per step using a PILATUS detector. The beam transmission, flux, beam size, and total angular range per data set were 100%, ∼2.0 × 10^11^ photons s^−1^, 30 × 30 µm, and 220°, respectively. The estimated maximum X-ray dose was 12.69 MGy^60^. The initial screening of 568 fragments and the DMSO optimization with tsA_2A_R LCP crystals were performed at the SLS, while the subsequent soaking (follow-up) of 109 fragments with tsA_2A_R LCP crystals was carried out at ESRF.

The data were processed and scaled using *XDS* and *XSCALE*^61^ with the *autoPROC* (1.1.7) pipeline^62^. *STARANISO* was used to calculate the diffraction limit and for anisotropic correction. Automated molecular replacement was carried out with *DIMPLE* (2.6.2) from *CCP*4^63^ using the tsA_2A_R structure (PDB entry 5IU4) without any ligand as a template. *PanDDA*^64^ was used for the automated detection and analysis of weakly bound fragments. *Coot* (0.9.8.96)^65^ was used for the manual inspection of fragment-bound structures and model building. *Phenix.refine*^66^, *BUSTER*^67^, and *REFMAC*^68^ were used for structure refinement. *REFMAC* (5.8.0267) was used during *PanDDA* (0.2.14) analysis, and *BUSTER* (2.10.4) was applied to refine ambiguous hits. Furthermore, all final fragment hits were ultimately refined using Phenix (1.20_4459) to ensure consistency in the reported statistics. All the *F*_*o*_*–F*_*c*_ maps shown (green mesh) were generated by *DIMPLE* after removing the ligand. Missing reflections in the input data were not filled with *F*_*c*_; the maps, therefore, reflect only the experimentally observed structure factors. Data collection and refinement statistics are reported in Supplementary Data 2–4. Structure figures were generated with *PyMOL* (3.1.0)^69^ and the chemical structures of the fragments were generated by DataWarrior^56^ and PubChem Sketcher V2.4^70^.

### Tethered docking and pose evaluation

To explore the potential for expanding the observed crystallographic fragment hits into follow-up compounds, we employed a tethered docking approach using a semi-automated pipeline implemented in Python (version 3.11.6) within a *Docker* environment based on Ubuntu 18.04, ensuring reproducibility across systems. Fragment analogs were retrieved from ChemSpace (https://chem-space.com) and MolPort (https://www.molport.com) in digital format. These analogs were pre-aligned to the crystallographic fragments using *RDKit* (version 2023.09.2; RDKit: Open-source cheminformatics, https://www.rdkit.org), tethering each ligand to the fragment’s MCS.

Docking was conducted with *rDock*^71^, fixing rotational and translational motions in the tethered pose while allowing torsional flexibility, generating up to ten poses per compound. This tethered docking technique drew methodological inspiration from an approach detailed in a blog post^72^ and enabled consistent alignment with the fragment’s MCS. The lists of docking poses were merged and converted to MOL2 format using *fconv*^73^ for subsequent re-scoring with *DrugScoreX*^74^, followed by clustering and selection of the highest-ranking poses for each compound.

An additional filtering step created Minimal Chemotype (MCT) representatives by iteratively removing non-essential substituents (e.g., terminal halogens and simple alkyl groups), yielding a canonicalized SMILES for each MCT. From all compounds sharing the same MCT, only the best-scoring representative pose was retained, ensuring that each MCT preserved the most favorable binding configuration and captured the core chemotype while maintaining key interaction motifs. This approach consolidated chemically distinct but structurally related hypotheses.

For triage, DSX-scored poses were visualized in *PyMOL*^69^. After further refinement of the selection, a final set of 109 compounds was acquired for experimental validation. Detailed information on the pipeline and methodology is available in the supporting information.

### Grating-coupled interferometry (GCI) screening of follow-up candidates

Direct interaction analysis of 109 follow-up candidates (ChemSpace) was performed using GCI on a Creoptix WAVEdelta system (Malvern Panalytical). Four channels of a PCP-NTA sensor chip were conditioned according to the manufacturer’s specifications. The surface is activated with a 420 s injection of 0.5 mM NiCl_2_ solution. Purified wtA_2A_R, tsA_2A_R, and a non-related reference GPCR (ts GPR17) were diluted into running buffer (40 mM Tris pH 7.5, 200 mM sodium chloride, 0.005% (v/v) LMNG/ 0.0005% (v/v) CHS) to 0.15 mg ml^−1^ and injected in subsequent pulses to separate flow channels at a flow rate of 2 μl min^−1^ to reach an immobilization level around 10,000 pg mm^−2^. The GPR17 construct used as negative control on GCI corresponds to residue 1–342 of human GPR17 (The GPR17 construct includes an N-terminal HA signal sequence and FLAG tag. The C-terminus was truncated at residue 342 and replaced with a 3 C cleavage site, followed by a 10 × His tag, a TEV cleavage site, and eGFP to aid purification. To improve thermal stability, seven mutations (D69V, C108Y, A155L, S315V, S107L, F158Y, and T230L) were introduced into the wild-type sequence). The surface of all channels was stabilized with several injections of running buffer. Analytes (follow-up candidates) were diluted from stock solutions at 200 mM in DMSO directly into running buffer (40 mM Tris pH 7.5, 200 mM sodium chloride, 0.005% (w/v) LMNG/ 0.0005% (w/v) CHS) to a concentration of 200 μM. The analyte solutions are injected over the stabilized flow channels using waveRAPID method^38^ based on 6 pulsed injections from the same stock solution with increasing pulse-width to generate varying analyte concentrations over time. Sensor response is monitored over a series of injection pulses, each with a fixed association and dissociation time, followed by a longer final dissociation time, in a single-cycle type experiment without regeneration of the surface between injections. Pulsed injections were performed using settings determined to be optimal for weak binders: 5 s of association time, followed by 20 s of dissociation time at a flow rate of 100 µl min^−1^. Realtime sensor signals from target and reference channels were recorded at 400 Hz. Signals from the target channel with A_2A_R were double-referenced, subtracting blank injections (running buffer) and reference channel signals and fit to a 1:1 interaction model using the WAVEcontrol software. The referenced sensor response as a discontinuous series of dissociation phases with masked association phases, bulk mismatch, and injection switching artifacts. The full assay was performed in the presence of the orthosteric ligand ZM241385^35–37^ at saturating concentration in all buffers and solutions throughout the experiment. Interacting analytes were defined based on filtering of evaluated parameters from the fitting, using statistical errors (confidence level) calculated for association (*k*_a_) and dissociation (*k*_d_) rate constants as well as the R_max_ calculated from fitting to a 1:1 interaction model. In addition, all sensorgrams were visually inspected, and fits optimized where applicable. Positive interaction signals after double-referencing, with calculated *k*_a_ and *k*_d_ errors below 100% and R_max_ smaller or equal to 20 pg mm^−2^ were defined as hits. These criteria provide filtering for interactions resembling stoichiometric 1:1 binding. Finally, some additional hits not fulfilling the initial hit-calling criteria were identified based on visual inspection of all signals. R_max_ values and confidence parameters (errors) of kinetic parameters derived from the fits were only used for hit-calling. The kinetic and affinity parameters from the fitting cannot be used for confident ranking of interactions.

In a subsequent assay, the 19 fragments identified as hits in both GCI screening assays as well as in the X-ray screen were re-tested for validation in another GCI waveRAPID experiment, using slightly longer association times (15 s) followed by 60 s of dissociation time as compared to the initial screening. Higher fragments concentration of 400 μM were also tested for validation of these 19 hits (Supplementary Data 6). Dose-response curves for eight selected fragments were further tested using traditional MCK with a 6-point concentration series of the compounds between 5 and 400 μM.

### Reporting summary

Further information on research design is available in the Nature Portfolio Reporting Summary linked to this article.

## Supplementary information

Transparent Peer Review file

SUPPLEMENTAL MATERIAL

Description of Additional Supplementary Files

Data Set 1

Data Set 2

Data Set 3

Data Set 4

Data Set 5

Data Set 6

Data Set 7

Life sciences reporting summary

## Data Availability

The structure and structure factors for A_2A_ receptor complexes with fragments have been deposited in the Protein Data Bank under the accession codes listed below. The associated statistics are provided in Supplementary Data 2–4. 7IN5, 7IN6, 7IN7, 7IN8, 7IN1, 7IN2, 7IN3, 7IN4, 7IN9, 7INA, 7INB, 7INC, 7IND, 7INE, 7INF, 7ING, 7INH, 7INI, 7INJ, 7INK, 7INL, 7INM, 7INN, 7INO, 7INP, 7INQ, 7INR, 7INT, 7INU, 7INV, 7INW, 7INX, 7INY, 7INZ, 7IO0, 7IO1, 7IO2, 7IO3, 7IO4, 7IO5, 7IO6, 7IO7, 7IO8, 7IO9, 7IOA, 7IOB, 7IOC, 7IOD, 7IOE, 7IOF, 7IOG, 7IOH, 7IOI, 7IOJ, 7IOK, 7IOL, 7IOM, 7ION, 7IOO, 7IOP, 7IOQ, 7IOR, 7IOS, 7IOT, 7IOU, 7IOV, 7IOW, 7IOX, 7IOY, 7IOZ, 7IP0, 7IP1, 7IP2, 7IP3, 7IP4, 7IP5, 7IP6, 7IP7, 7IP8, 7IP9, 7IPA, 7IPB, 7IPC, 7IPD, 7IPE, 7IPF, 7IPG. All relevant data supporting the findings of this study are available from the corresponding author, Chia-Ying Huang (chia-ying.huang@psi.ch), upon reasonable request.
