# Supplementary material for: An integrated experimental and computational pipeline for crystallographic fragment screening of membrane protein in the lipid cubic phase
Source: Commun Chem. 2026 May 13;9:257. doi: 10.1038/s42004-026-02059-7 (PMC13402683; doi:10.1038/s42004-026-02059-7)
Supplement: Supplementary file 2 — SUPPLEMENTAL MATERIAL [file 42004_2026_2059_MOESM2_ESM.pdf]

Supplementary Information

An Integrated Experimental and Computational Pipeline for Crystallographic Fragment Screening of Membrane Proteins in the Lipid Cubic Phase

Chia-Ying Huang[a*], Robert Cheng[b], Alexander Metz[a,c], Denis Bucher[b], Fabio Andres[b], Arianna Bacchin[b], Hannah Glover[b], Christoph P. Sager[b], Meitian Wang[a], Michel O. Steinmetz[d,e], Michael Hennig[b], and May Sharpe[a]

a. PSI Center for Photon Sciences, Forschungsstrasse 111, 5232 Villigen PSI, Switzerland

b. leadXpro AG, Park Innovaare, 5234 Villigen, Switzerland

c. Current address: UCB, Braine-l'Alleud, Wallonische Region, Belgien

d. PSI Center for Life Sciences, Forschungsstrasse 111, 5232 Villigen PSI, Switzerland

e. Biozentrum, University of Basel, 4056 Basel, Switzerland

* Corresponding email to: chia-ying.huang@psi.ch

Supplementary Method

Figure S1-14

Supplementary Data 1-7 are provided in separate Excel files

Supplementary Method

Tethered Docking and Pose Evaluation

The source code for the previously published software *fconv* [1] and *DrugScoreX* (DSX) [2] is available at https://github.com/gneu77/fconv-dsx.

To explore how to expand the crystallographic fragment hits into follow-up compounds, we implemented a tethered docking [3] approach utilizing a semi-automated pipeline. This pipeline was implemented in Python 3.11.6 within a Docker environment based on Ubuntu 18.04, ensuring reproducibility and consistent dependency management across systems. Key functionalities were managed through Python scripts and Conda-managed packages.

Fragment analogs were retrieved via the APIs (application programming interfaces) of ChemSpace (https://chem-space.com) and MolPort (https://www.molport.com) in digital format, using a combination of substructure and similarity searches. A Tanimoto similarity cutoff of 0.65 was applied in the similarity search. Additional queries pruned the fragment to emphasize relevant structural motifs or adjust tautomeric states as necessary. The SDF reference fragment was converted to SMILES (simplified molecular input line entry system) format using *RDKit* (version 2023.09.2; RDKit: Open-source cheminformatics. https://www.rdkit.org), undergoing sanitization and neutralization steps to ensure a stable, consistent representation for retrieval queries. Compounds were filtered to retain only organic molecules, eliminating any containing inorganic elements.

Following retrieval, compounds were pre-aligned and tethered to the maximum common substructure (MCS) of the crystallographic fragment pose using *RDKit*. This step ensured spatial alignment with the reference fragment by anchoring each ligand to the fragment's MCS. For ligands with symmetrical MCSs, the script used RDKit's GetSubstructMatches function to explore all possible alignments, generating multiple valid orientations. Each alignment was minimized with the Universal Force Field [4] to achieve a relaxed conformation before docking, with the fragment core atoms fully fixed while allowing torsional flexibility within the ligands.

To remove redundant tethered poses, we applied a filtering step that retained only unique orientations for each compound. This step was necessary because the previous alignment process exhaustively explored all possible substructure matches, producing quasi-identical poses. We used RDKit's RMSD-based clustering method with a 0.2 Å cutoff to group similar orientations, selecting one representative pose per cluster.

To prepare compounds for subsequent docking with *rDock*[5], all non-polar hydrogens were removed using *RDKit*. Compound size was generally capped at 35 non-hydrogen atoms, but if this yielded too many compounds for practical docking, the limit was reduced, with a minimum set at fragment size plus 10 non-hydrogen atoms. The docking cavity was defined using rbcavity, centered on the crystallographic fragment pose, with default settings except for RADIUS (12.0 Å), SMALL_SPHERE (1.0 Å), and MAX_CAVITIES (1).

Docking was conducted using *rDock* (GitHub commit d6fd39ca), with the ligands fully fixed in both translational and rotational dimensions to preserve alignment with the fragment's MCS.

Only torsional flexibility was allowed, enabling the ligands to explore binding interactions within the pocket, with 10 docking poses generated per tethered compound orientation. This tethered docking approach drew methodological inspiration from an approach detailed in a blog post [6].

After docking, all poses for each compound, including those from each tethered orientation, were merged into a single SDF file and converted to MOL2 format using *fconv* [1] for subsequent re-scoring with *DrugScoreX* [2]. DSX re-scoring, with equal weightings for scoring terms (T0 - T4), annotated each pose with a normalized DSX score per non-hydrogen atom within the SDF file. Using these scores, poses were filtered to retain only those with a DSX score below -20.0.

The remaining poses for each compound were clustered in place (i.e., without pose realignment) using *fconv* for complete linkage clustering, with an RMSD cutoff of 1 Å. For each compound, up to three best-scoring clusters were sorted by DSX score, retaining the highest-ranking pose per cluster. Finally, all poses across compounds were organized by each ligand's best overall DSX score, preserving these ranked triplets to streamline visual comparisons of different poses of the same compound.

After the DSX re-scoring and clustering, an additional filtering step was applied to streamline the selection of chemically distinct hypotheses for fragment hit expansion. Each pose was processed to generate a Minimal Chemotype (MCT) SMILES, capturing the essential chemotype of each compound while omitting non-critical substituents. The MCT generation involved iteratively removing halogens and simple terminal alkyl groups (methyl, methylene, methine, and cyclopropyl), ensuring that all hydrogen bond donors or acceptors were retained to preserve the chemotype characteristics. SMILES of the MCTs were canonicalized to ensure consistent comparisons of compounds sharing the same MCT. For all compounds sharing the same MCT, only the pose of the original, unmodified compound with the lowest DSX score per non-hydrogen atom was retained as the Minimal Chemotype Representative, preserving the most favorable configuration within each chemotype category. This approach allowed for the consolidation of chemically distinct representatives while ensuring that the final selection included only the optimal binding pose for each chemotype, representing a unique hypothesis.

For triage, the remaining poses were visualized in *PyMOL* [7] using DSX-based visualizations that highlighted each atom's contribution to the overall DSX score, potential polar interactions, steric clashes between the docking pose and protein, and any unusual torsion angles. In a rapid initial selection, approximately 1,000 poses per fragment hit were reviewed. The selected set was further refined in a second round of triage, focusing on the plausibility of interactions and compound conformations, with particular attention to expanding the starting fragments into diverse and feasible motifs capable of interacting with key amino acids Asn38 and Asn42, as well as additional features within the binding pocket.

In the final triage round, poses and chemotypes were compared extensively to identify the best candidates among similar hypotheses. Vendor catalog prices, retrieved via their APIs, were a secondary consideration in selecting among similar hypotheses. In cases where the exact docked compound was unavailable or prohibitively priced, close analogs adhering to the Minimal Chemotype hypothesis were substituted and assessed for compatibility with the predicted binding pose. Ultimately, 109 compounds representing unique chemotype and interaction hypotheses were acquired for experimental evaluation.

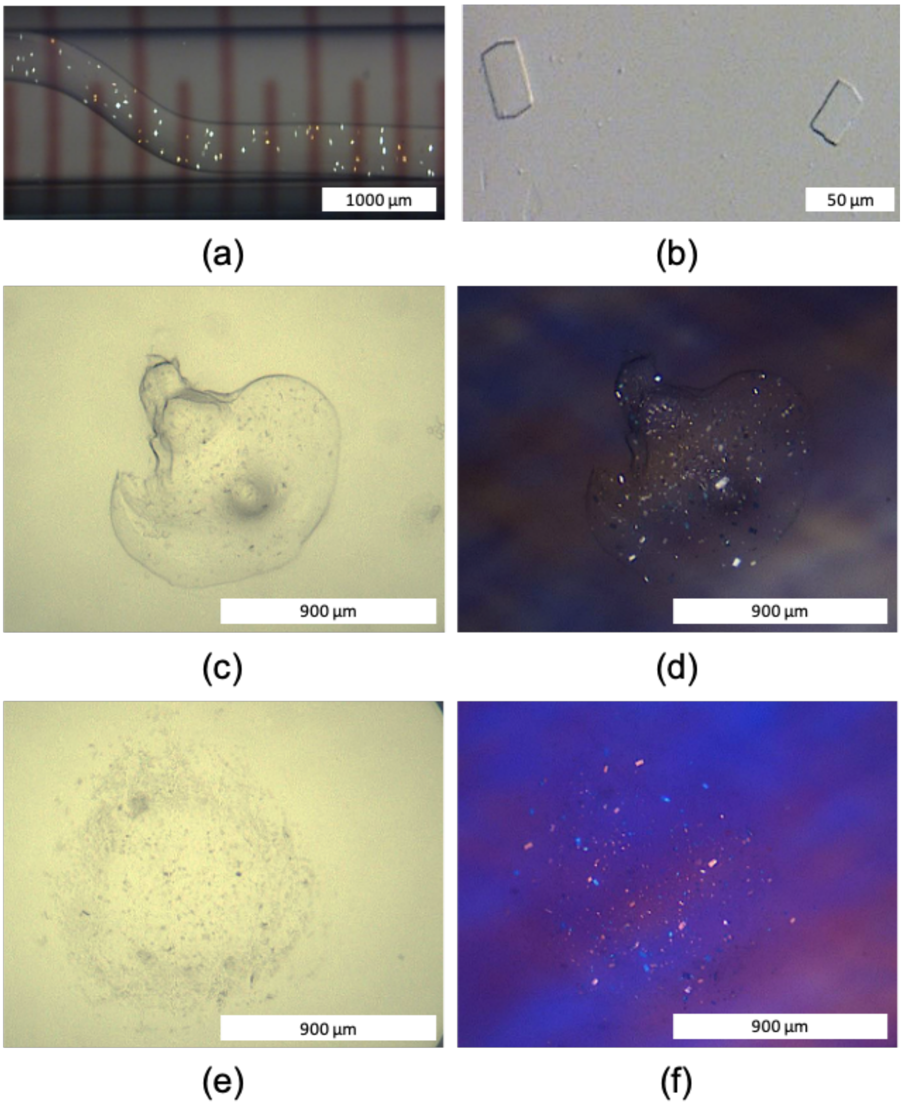

Figure S1. tsA$_{2A}$R crystal images. (a) tsA$_{2A}$R crystal-laden LCP in a syringe viewed under cross-polarized light, (b) tsA$_{2A}$R crystal on a glass plate viewed with standard microscope lighting, (c) tsA$_{2A}$R crystal on an SwissCI-3-lens crystallization plate imaged with the Rock Imager under standard lighting, and (d) the same crystal under cross-polarized light. (e) The same tsA$_{2A}$R crystal as in (c), soaked with a fragment at 10 mM in 10% DMSO and imaged with the Rock Imager under standard lighting, and (f) the same crystal as in (d) viewed under cross-polarized light.

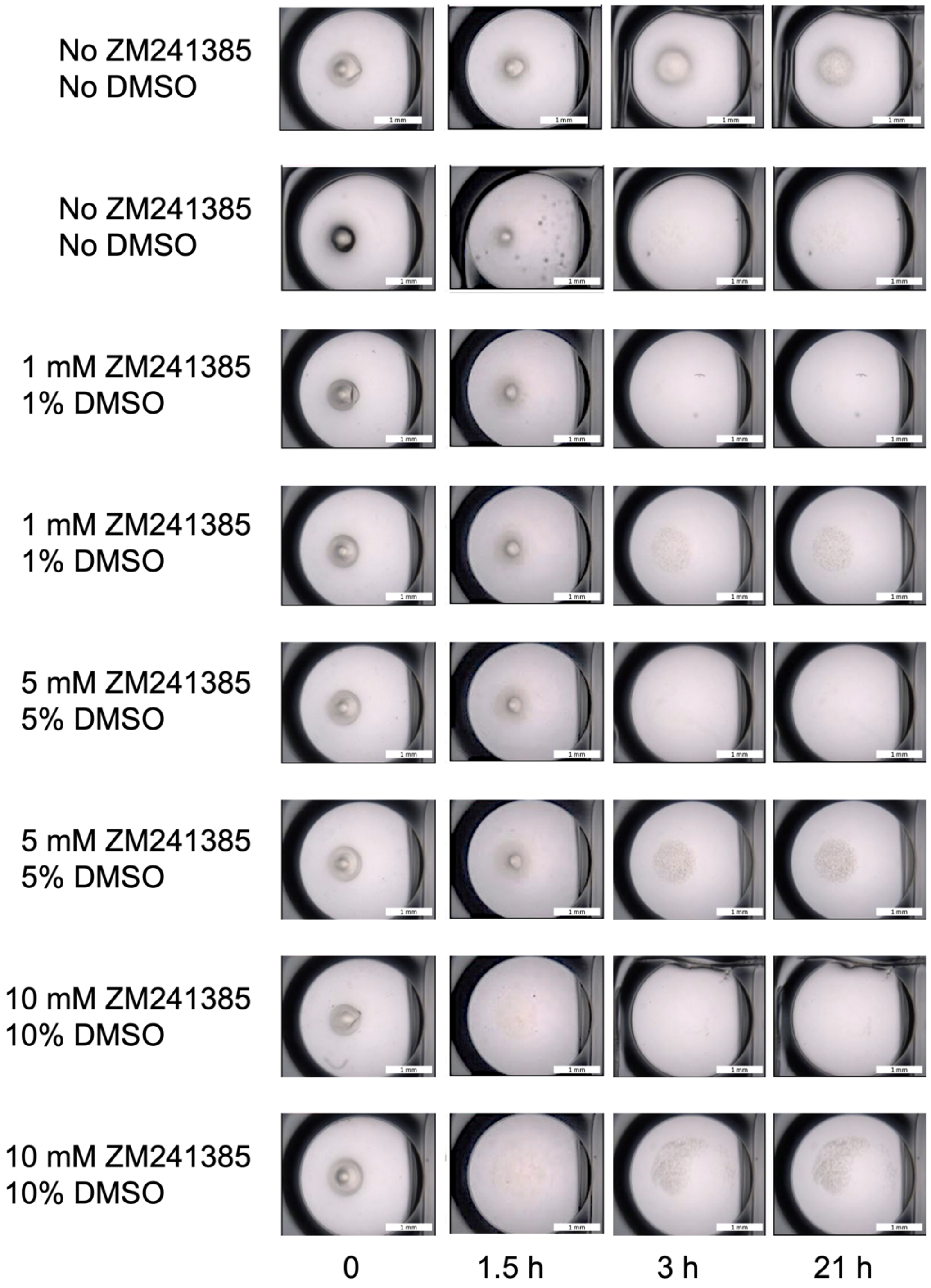

Figure S2. tsA$_{2A}$R-laden LCP behavior under varying DMSO concentrations. Testing was conducted using ZM241385 and tsA$_{2A}$R-laden LCP samples in the SwissCI-3-lens crystallization plates. Each condition was tested in duplicate to ensure reliability and reproducibility.

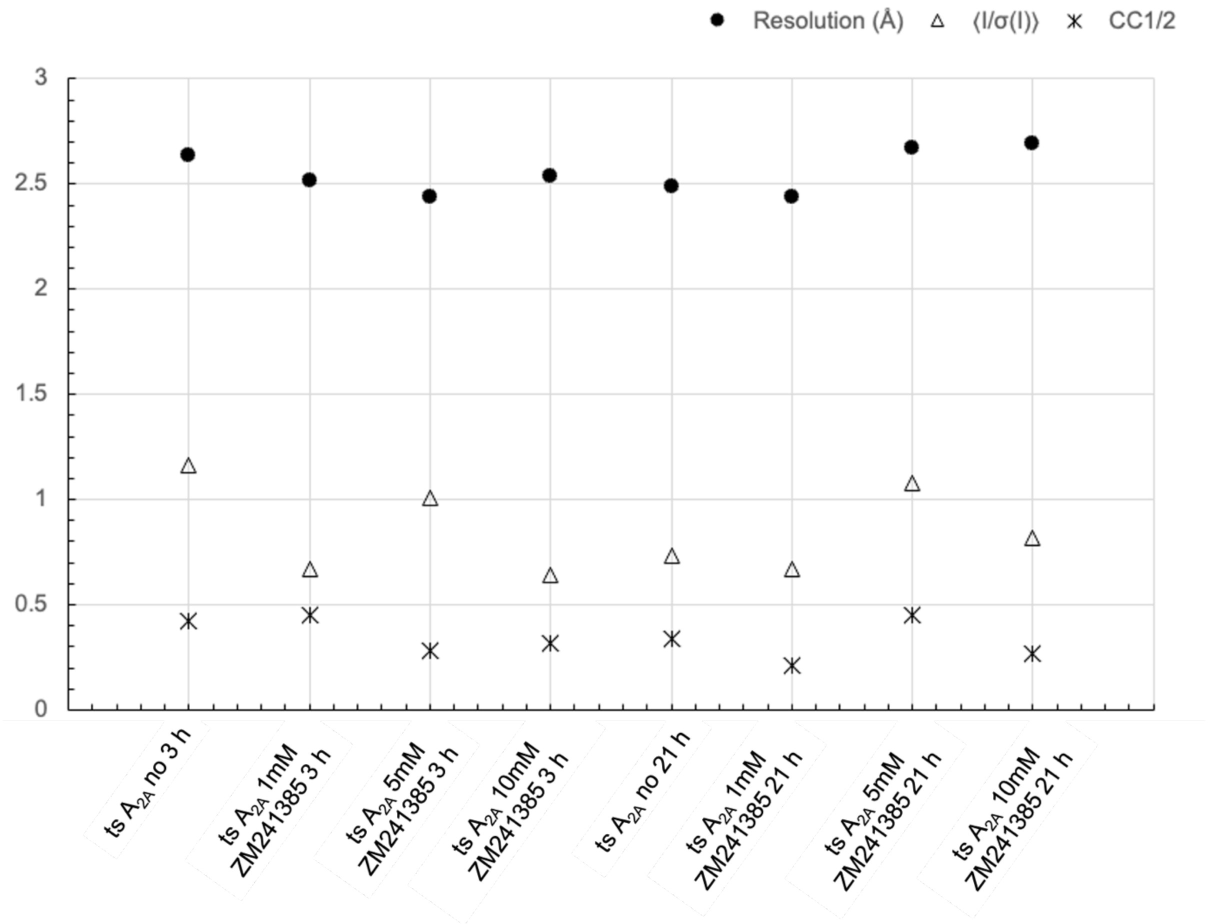

Figure S3. Statistics of the data sets of serial DMSO testing and ZM241385 replacement in tsA$_{2A}$R crystals. Here shows the resolution (Å), ⟨I/σ(I), and CC$_{1/2}$ of the highest resolution shell of each data set.

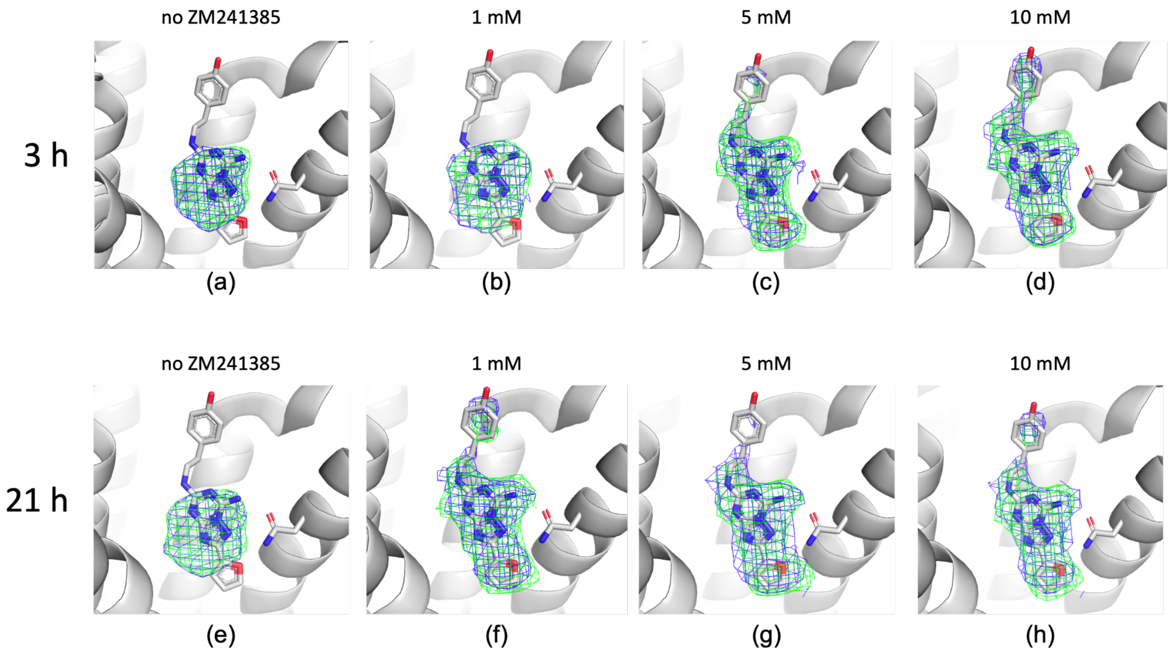

	3 h, 1 mM ZM241385	3 h, 5 mM ZM241385	3 h, 10 mM ZM241385
**B-factor**	-	55.77	59.95
**RSCC**	-	0.953	0.927
**Occupancy**	-	0.92	1
	**21 h, 1 mM ZM241385**	**21 h, 5 mM ZM241385**	**21 h, 10 mM ZM241385**
**B-factor**	62.75	57.53	64.32
**RSCC**	0.935	0.946	0.936
**Occupancy**	0.98	0.99	0.95

Figure S4. Serial DMSO testing and ZM241385 replacement of theophylline at the tsA$_{2A}$R extracellular site. The first and second rows show the tsA$_{2A}$R LCP crystals soaked with fragments for 3 h (b-d) and 21 h (f-h), respectively, with serial DMSO/ZM241385 concentration. PDB 5IU4 was used to superimpose the map, displayed in cartoon representation, with ZM241385 depicted in stick representation. The $2F_o - F_c$ electron-density maps are contoured at the 1.0 σ level with blue-colored mesh around the ZM241385. The $F_o - F_c$ electron-density maps generated using the model without the ligand are also shown contoured at the 3.0 σ level and colored as a green mesh. A table below presents the B-factor, real-space correlation coefficient (RSCC), and occupancy of 5 or 10 mM ZM241385 at various soaking times.

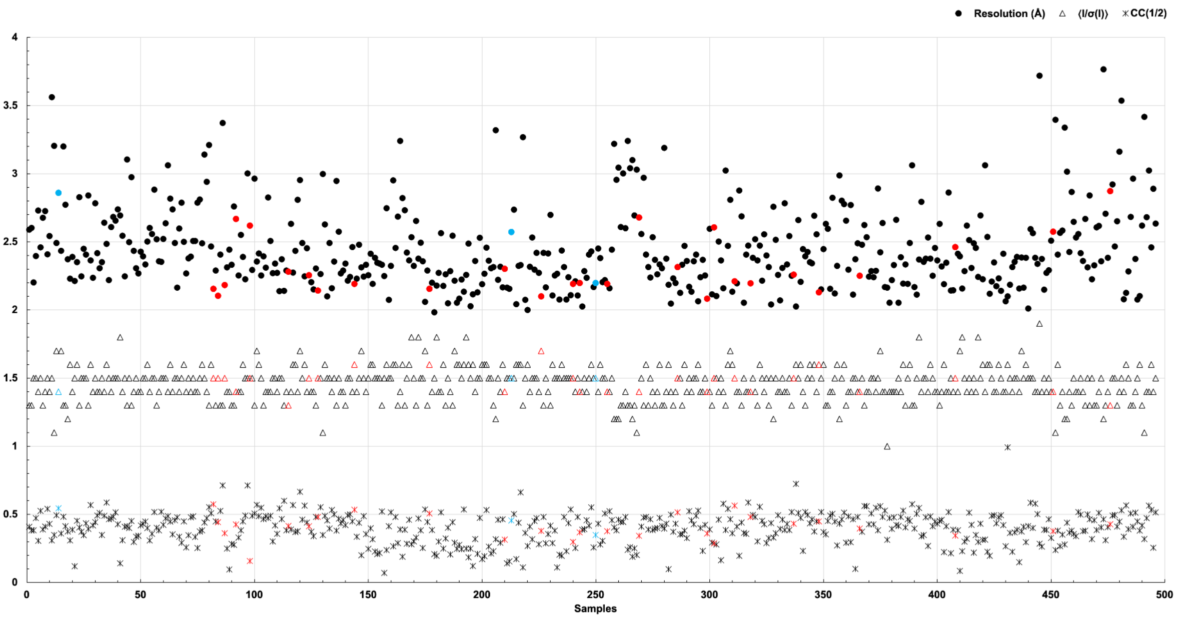

Figure S5. Statistics of X-ray data sets for tsA$_{2A}$R with 498 fragments. Here shows the resolution (Å), ⟨I/σ(I)⟩, and CC$_{1/2}$ of the highest resolution shell of each data set. The hits at the intracellular of tsA$_{2A}$R are indicated in red color, and at the extracellular of tsA$_{2A}$R are indicated in blue color.

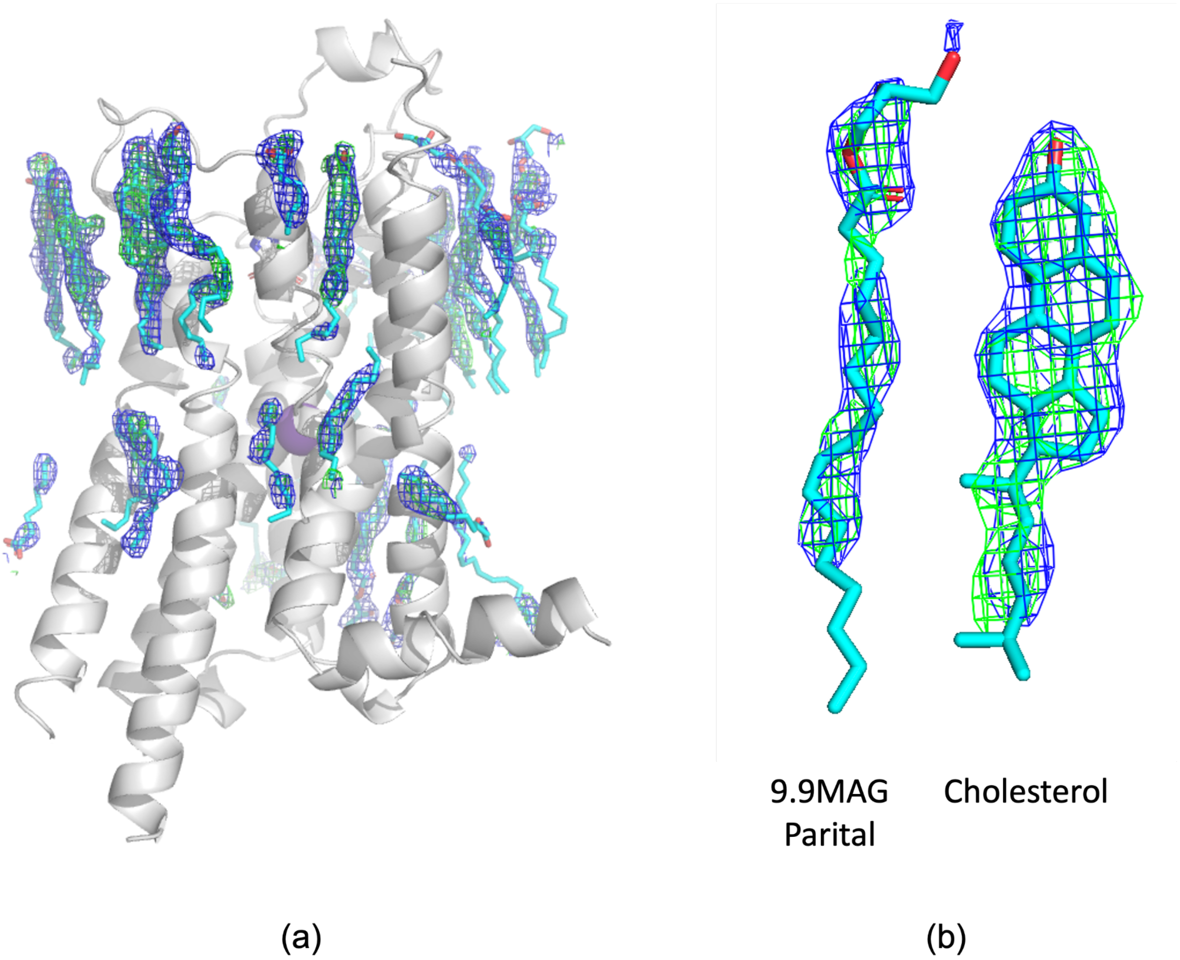

Figure S6. Overall structure of tsA$_{2A}$R with electron density map of 9.9 MAGs and cholesterol. (a) The protein is shown in cartoon representation with gray color and the surrounding 9.9 MAGs and cholesterol are shown in stick representation with cyan color. The $F_o - F_c$ electron density maps, calculated using a FU$_{163}$-106 excluding the lipid, are displayed as a green mesh contoured at the 2.5 σ level. The $2F_o - F_c$ electron density maps, calculated using the model with lipid, are displayed as a green mesh contoured at the 1.0 σ level.

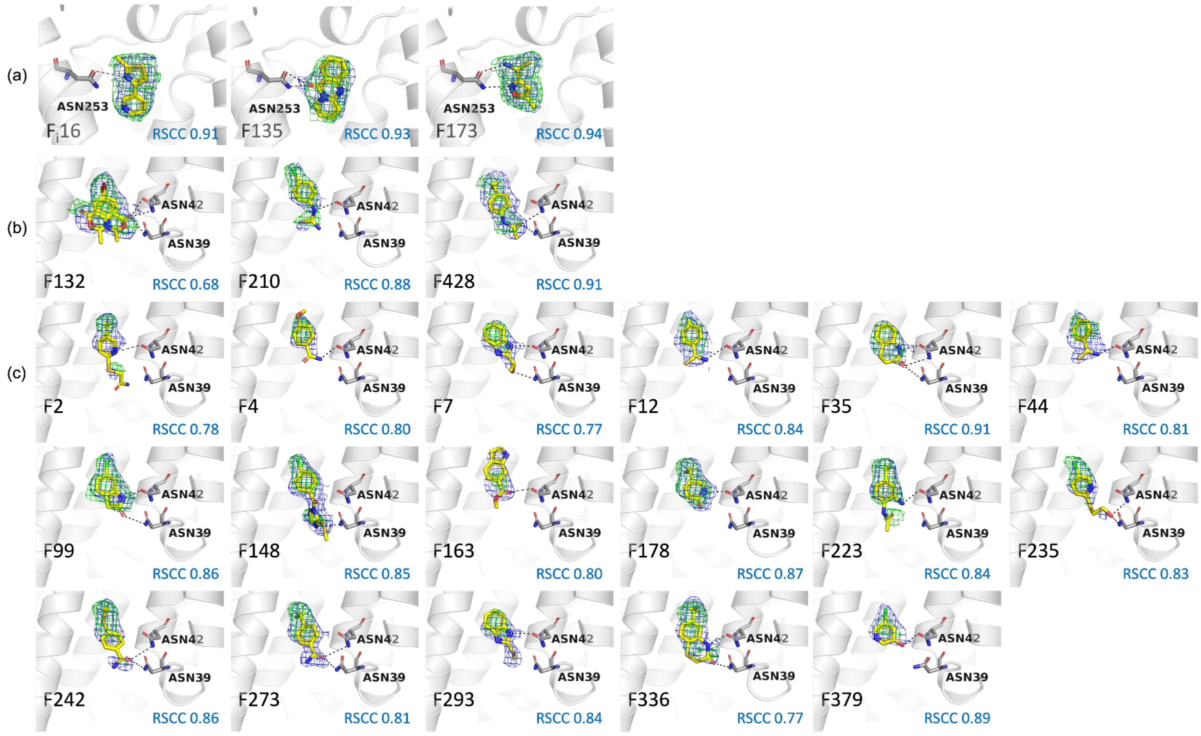

Figure S7. Initial 23 hits out of 568 fragments screened with LCP-CFS. The $tsA_{2A}R$ around the extracellular pocket for (a) and intracellular pocket for (b-c) are shown in cartoon representation with the gray color and with the intracellular site oriented face-down. The identified fragments are shown in stick representation and highlighted in yellow. The identified fragments, along with residues ASN253 for extracellular pocket, and ASN39 and ASN42 for intracellular pocket, are shown in stick representation and highlighted gray. The $F_o - F_c$ electron density maps, calculated using a model (PDB 5IU4) excluding the ligand, are displayed as a green mesh contoured at the 2.5 σ level. The $2F_o - F_c$ electron density maps, calculated using the model with ligand, are displayed as a green mesh contoured at the 1.0 σ level. RSCC values were taken from the wwPDB validation report, which uses filled 2mFo–DFc maps (FWT/PHWT) with a standard soft mask.

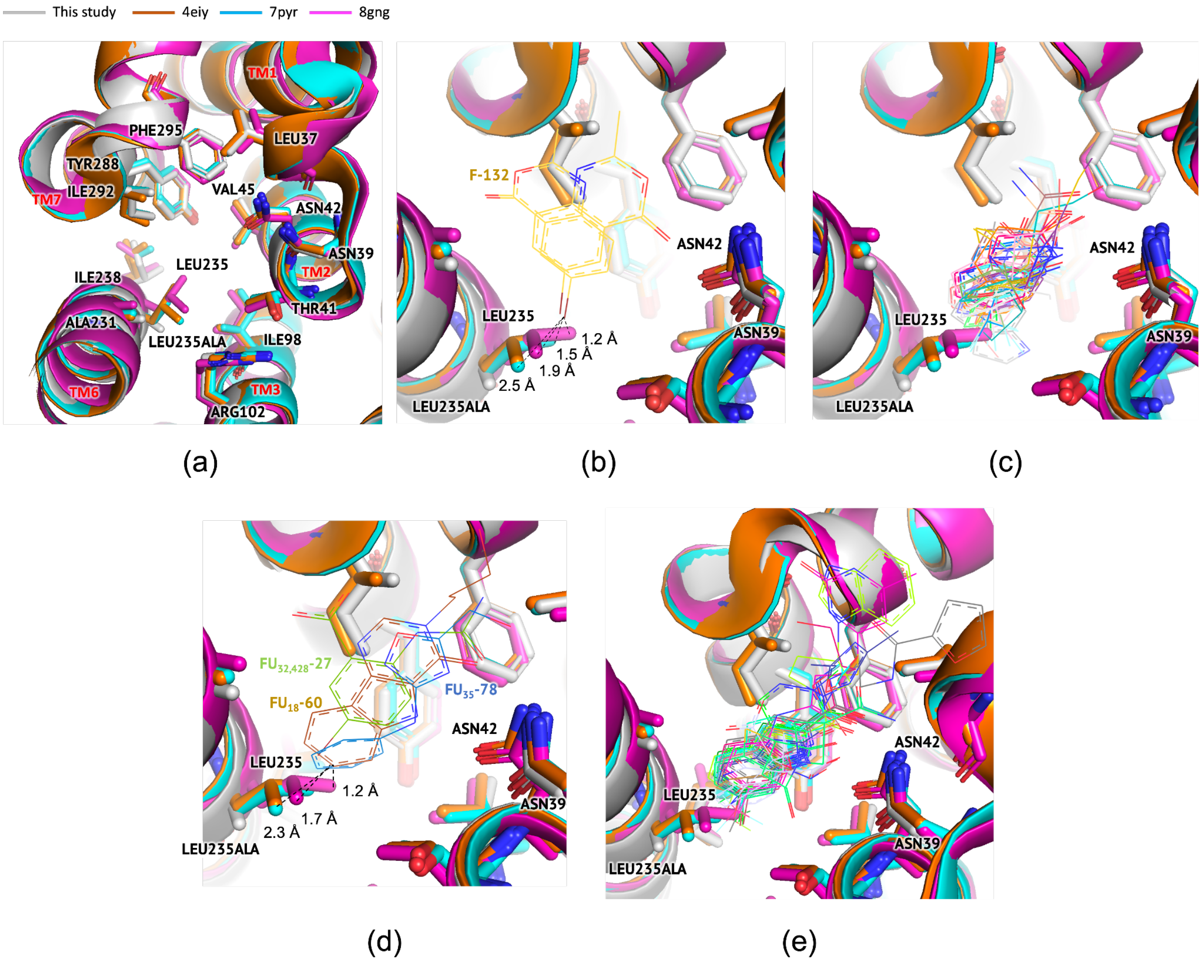

Figure S8. View of the intracellular site of the A_{2A} receptors. (a) A funnel-shaped cavity lined by transmembrane helices TM2, TM3, TM6, and TM7 of A_{2A} receptors, with the relevant residues labeled. (b) The closest distance between F132 and LEU235 as observed in structures PDB 8GNG and PDB 7PYR. (c) Steric clash between the rest of the initial fragment hits and LEU235 in PDB 8GNG and PDB 7PYR. (d) The closest distance between follow-up hits $FU_{32,428}$-27, FU_{18}-60, FU_{35}-78, and LEU235 as observed in structures PDB 8GNG and PDB 7PYR. (e) Steric clash between the rest of the follow-up hits and LEU235 in PDB 8GNG and PDB 7PYR. The A_{2A} receptors are shown in cartoon representation, with coloring as indicated in the figure, and viewed from the intracellular side. The identified fragments are shown in line representation and highlighted in different colors.

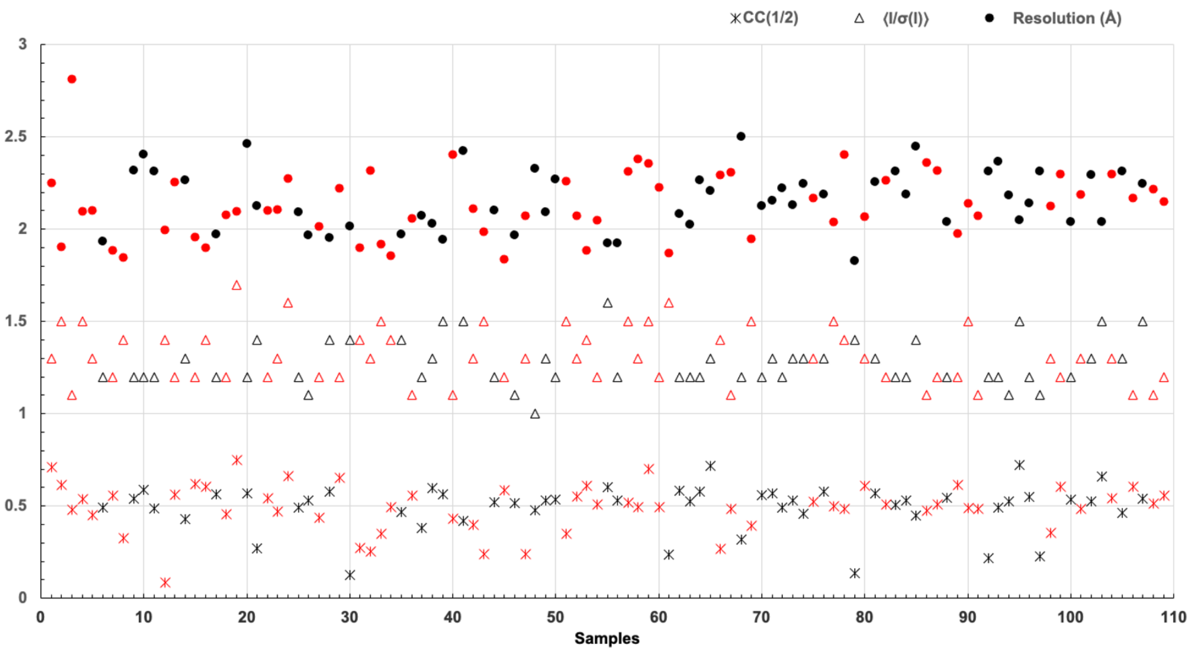

Figure S9. Statistics of X-ray data sets for $tsA_{2A}R$ with follow-up fragments 1 to 109. Here shows the resolution (Å), ⟨I/σ(I), and $CC_{1/2}$ of the highest resolution shell of each data set. The hits are indicated in red color.

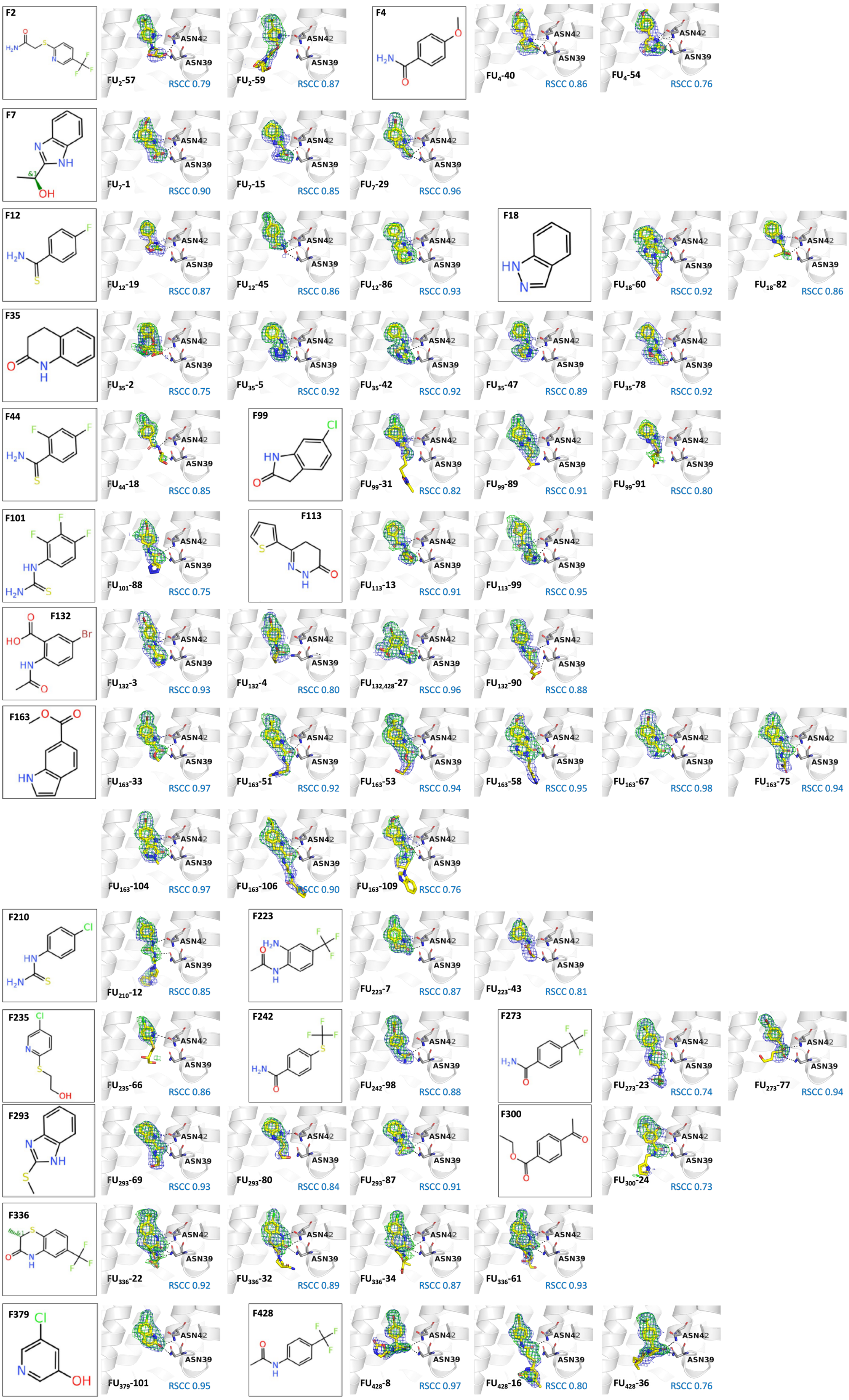
ASN42
ASN39
F2
FU$_{2}$-57 RSCC 0.79
FU$_{2}$-59 RSCC 0.87
F4
FU$_{4}$-40 RSCC 0.86
FU$_{4}$-54 RSCC 0.76
F7
FU$_{7}$-1 RSCC 0.90
FU$_{7}$-15 RSCC 0.85
FU$_{7}$-29 RSCC 0.96
F12
FU$_{12}$-19 RSCC 0.87
FU$_{12}$-45 RSCC 0.86
FU$_{12}$-86 RSCC 0.93
F18
FU$_{18}$-60 RSCC 0.92
FU$_{18}$-82 RSCC 0.86
F35
FU$_{35}$-2 RSCC 0.75
FU$_{35}$-5 RSCC 0.92
FU$_{35}$-42 RSCC 0.92
FU$_{35}$-47 RSCC 0.89
FU$_{35}$-78 RSCC 0.92
F44
FU$_{44}$-18 RSCC 0.85
F99
FU$_{99}$-31 RSCC 0.82
FU$_{99}$-89 RSCC 0.91
FU$_{99}$-91 RSCC 0.80
F101
FU$_{101}$-88 RSCC 0.75
F113
FU$_{113}$-13 RSCC 0.91
FU$_{113}$-99 RSCC 0.95
F132
FU$_{132}$-3 RSCC 0.93
FU$_{132}$-4 RSCC 0.80
FU$_{132,428}$-27 RSCC 0.96
FU$_{132}$-90 RSCC 0.88
F163
FU$_{163}$-33 RSCC 0.97
FU$_{163}$-51 RSCC 0.92
FU$_{163}$-53 RSCC 0.94
FU$_{163}$-58 RSCC 0.95
FU$_{163}$-67 RSCC 0.98
FU$_{163}$-75 RSCC 0.94
FU$_{163}$-104 RSCC 0.97
FU$_{163}$-106 RSCC 0.90
FU$_{163}$-109 RSCC 0.76
F210
FU$_{210}$-12 RSCC 0.85
F223
FU$_{223}$-7 RSCC 0.87
FU$_{223}$-43 RSCC 0.81
F235
FU$_{235}$-66 RSCC 0.86
F242
FU$_{242}$-98 RSCC 0.88
F273
FU$_{273}$-23 RSCC 0.74
FU$_{273}$-77 RSCC 0.94
F293
FU$_{293}$-69 RSCC 0.93
FU$_{293}$-80 RSCC 0.84
FU$_{293}$-87 RSCC 0.91
F300
FU$_{300}$-24 RSCC 0.73
F336
FU$_{336}$-22 RSCC 0.92
FU$_{336}$-32 RSCC 0.89
FU$_{336}$-34 RSCC 0.87
FU$_{336}$-61 RSCC 0.93
F379
FU$_{379}$-101 RSCC 0.95
F428
FU$_{428}$-8 RSCC 0.97
FU$_{428}$-16 RSCC 0.80
FU$_{428}$-36 RSCC 0.76

Figure S10. Follow-up compound and fragment hits of $tsA_{2A}R$. The structure in the square highlights the initial hits, with the corresponding compounds/fragments listed on its right-hand side. The $tsA_{2A}R$ around the intracellular pocket is shown in cartoon representation with the gray color and with the intracellular site oriented face-down. The identified fragments are shown in stick representation, and highlighted in yellow. The $F_o - F_c$ electron density maps, calculated using a model (PDB 5IU4) excluding the ligand, are displayed as a green mesh contoured at the 2.5 σ level. The $2F_o - F_c$ electron density maps, calculated using the model with ligand, are displayed as a green mesh contoured at the 1.0 σ level. RSCC values were taken from the wwPDB validation report, which uses filled 2mFo–DFc maps (FWT/PHWT) with a standard soft mask. F indicates the fragment, while FU represents the follow-up fragments designed based on the fragments in the subscript number.

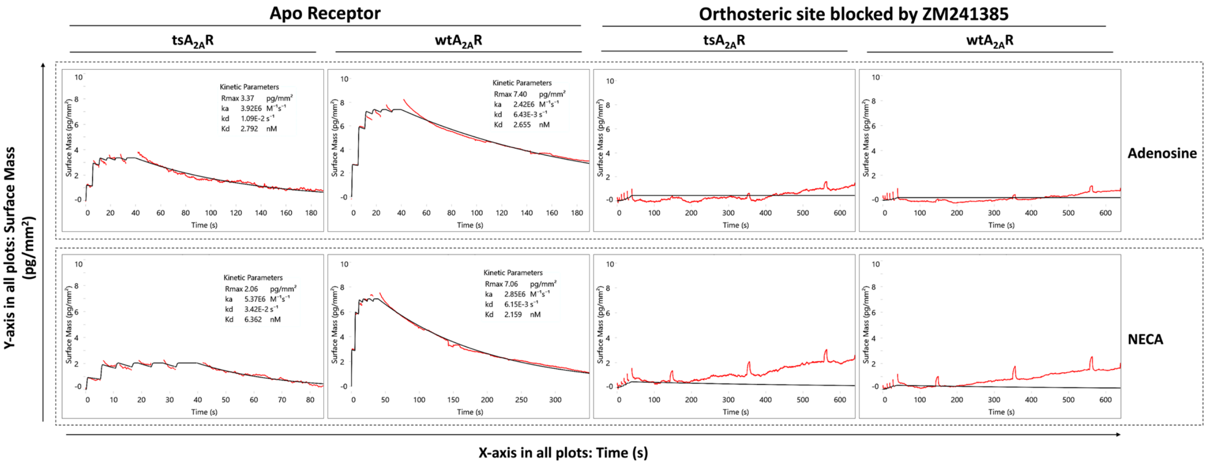

Figure S11. GCI waveRAPID assay controls. Control agonists, Adenosine and NECA, bind to $A_{2A}R$. Double-referenced sensor signals for waveRAPID pulsed injections generating dose responses up to 200 μM of the fragments are shown in red, 1:1 interaction models are shown as black lines. Y-axis in all plots of GCI waveRAPID is surface mass (pg/mm^2) and X-axis in all plots of GCI waveRAPID is time (s).

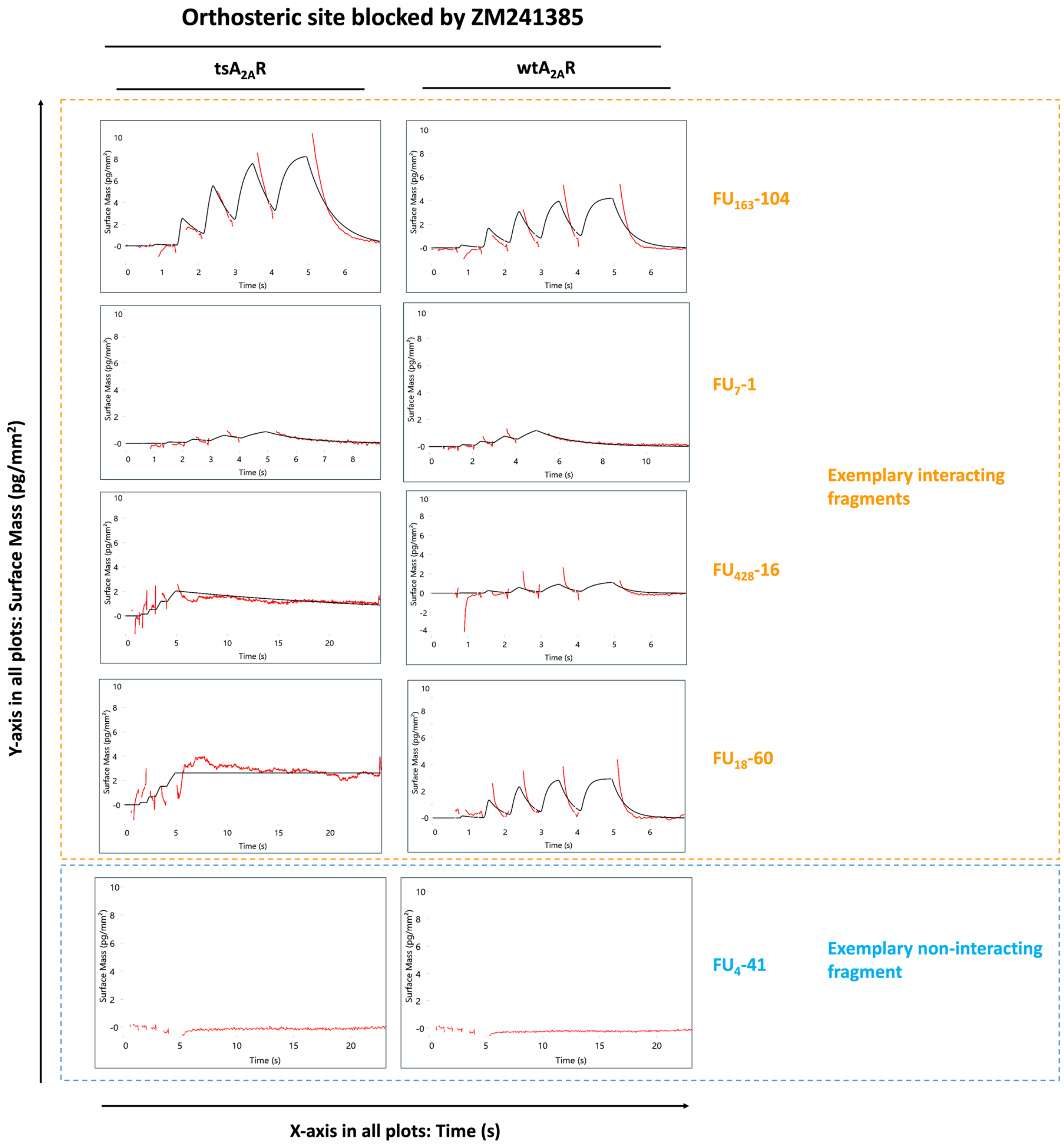

Figure S12. Example GCI waveRAPID sensorgrams of tested fragments. Interacting and non-interacting fragments binding to $A_{2A}R$ are shown in orange and blue dashed squares, respectively. Double-referenced sensor signals for waveRAPID pulsed injections generating dose responses up to 200 µM of the fragments are shown in red, 1:1 interaction models are shown as black lines. F indicates the fragment, while FU represents the follow-up fragments designed based on the fragments in the subscript number. Y-axis in all plots of GCI waveRAPID is surface mass (pg/mm^2) and X-axis in all plots of GCI waveRAPID is time (s).

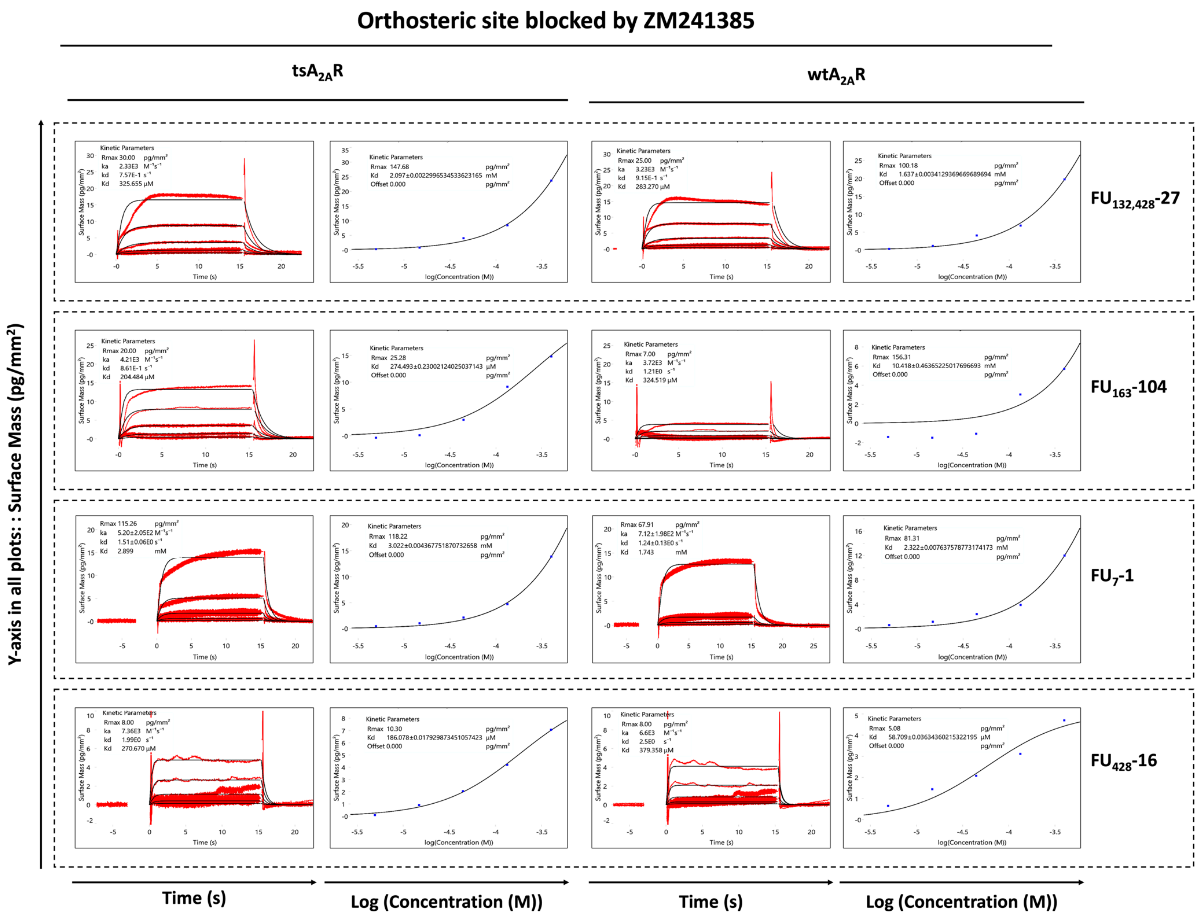

Figure S13. Example GCI Multi-cycle Kinetics (MCK) sensorgrams of tested fragments. Double-referenced sensor signals for 6-point dose responses from 5 µM to 400 µM of the fragments are shown in red, 1:1 interaction models are shown as black lines. Equilibrium plots from steady-state levels shown on the right of the respective sensorgram. The estimated kinetic rate constants, K_D and R_{max} values as shown on the plots are not considered precise and no interpretations are drawn from them. F indicates the fragment, while FU represents the follow-up fragments designed based on the fragments in the subscript number. Y-axis in all plots of GCI MCK is surface mass (pg/mm^2) and X-axis in a pair of left plot is time (s) and right plot is Log (Concentration (M))

Compound /fragment	GCI with orthosteric site blocked by ZM241385	X-ray
FU_{7}-1	■	■
FU_{35}-2	■	■
FU_{223}-7	■	■
FU_{428}-16	■	■
$FU_{132,428}$-27	■	■
FU_{7}-29	■	■
FU_{99}-31	■	■
FU_{336}-32	■	■
FU_{163}-33	■	■
FU_{12}-45	■	■
FU_{163}-53	■	■
FU_{18}-60	■	■
FU_{163}-75	■	■
FU_{163}-67	■	■
FU_{35}-78	■	■
FU_{379}-101	■	■
FU_{163}-104	■	■
FU_{163}-106	■	■
FU_{163}-109	■	■

Compound /fragment	GCI with orthosteric site blocked by ZM241385	X-ray
FU_{210}-14	■	
FU_{100}-28	■	
FU_{210}-39	■	
FU_{99}-52	■	
FU_{35}-62	■	
FU_{64}-72	■	
$FU_{132,428}$-74	■	
FU_{178}-76	■	
FU_{293}-85	■	
FU_{223}-94	■	
FU_{132}-100	■	
FU_{163}-105	■	
FU_{132}-3		■
FU_{132}-4		■
FU_{35}-5		■
FU_{428}-8		■
FU_{210}-12		■
FU_{113}-13		■
FU_{7}-15		■
FU_{44}-18		■
FU_{12}-19		■
FU_{336}-22		■
FU_{273}-23		■
FU_{300}-24		■
FU_{336}-34		■
FU_{428}-36		■
FU_{4}-40		■
FU_{35}-42		■
FU_{223}-43		■
FU_{35}-47		■
FU_{163}-51		■
FU_{4}-54		■
FU_{2}-57		■
FU_{163}-58		■
FU_{2}-59		■
FU_{336}-61		■
FU_{253}-66		■
FU_{293}-69		■
FU_{273}-77		■
FU_{293}-80		■
FU_{18}-82		■
FU_{12}-86		■
FU_{293}-87		■
FU_{101}-88		■
FU_{99}-89		■
FU_{99}-91		■
FU_{132}-90		■
FU_{242}-98		■
FU_{113}-99		■

Compound /fragment	GCI with orthosteric site blocked by ZM241385	X-ray
FU_{4}-6		
FU_{4}-9		
FU_{210}-10		
FU_{99}-11		
FU_{223}-17		
FU_{18}-20		
FU_{100}-21		
FU_{7}-25		
FU_{210}-26		
FU_{44}-30		
FU_{210}-35		
FU_{210}-37		
FU_{2}-38		
FU_{4}-41		
FU_{99}-44		
FU_{132}-46		
FU_{132}-48		
FU_{35}-49		
FU_{132}-50		
FU_{4}-55		
FU_{132}-56		
FU_{99}-63		
FU_{64}-64		
FU_{18}-65		
FU_{273}-68		
FU_{223}-70		
FU_{35}-71		
FU_{18}-73		
FU_{273}-79		
FU_{4}-81		
FU_{132}-83		
FU_{223}-84		
FU_{132}-92		
FU_{4}-93		
FU_{99}-95		
FU_{223}-96		
FU_{113}-97		
FU_{132}-102		
FU_{178}-103		
FU_{336}-107		
FU_{242}-108		

Figure S14. GCI interaction screening of 109 follow-up candidates. Follow-up candidates were tested for interaction with wtA_{2A}R and tsA_{2A}R with the orthosteric binding pocket blocked with ZM241385. Green cells for the GCI assays represent interactions with A_{2A}R as detected in the assay. F indicates the fragment, while FU represents the follow-up fragments designed based on the fragments in the subscript number.

Supplementary References

1. Neudert, G. & Klebe, G. fconv: Format conversion, manipulation and feature computation of molecular data. *Bioinformatics* **27**, 1021–1022 (2011).
2. Neudert, G. & Klebe, G. DSX: a knowledge-based scoring function for the assessment of protein-ligand complexes. *J. Chem. Inf. Model.* **51**, 2731–2745 (2011).
3. Miñarro-Lleonar, M., Ruiz-Carmona, S., Alvarez-Garcia, D., Schmidtke, P. & Barril, X. Development of an automatic pipeline for participation in the CELPP Challenge. *Int. J. Mol. Sci.* **23**, 4756 (2022).
4. Rappe, A. K., Casewit, C. J., Colwell, K. S., Goddard, W. A., III & Skiff, W. M. UFF, a full periodic table force field for molecular mechanics and molecular dynamics simulations. *J. Am. Chem. Soc.* **114**, 10024–10035 (1992).
5. Ruiz-Carmona, S. *et al.* rDock: a fast, versatile and open source program for docking ligands to proteins and nucleic acids. *PLoS Comput. Biol.* **10**, e1003571 (2014).
6. Schmidtke, P. Tethered minimization of small molecules with RDKit. *Discngine* https://www.discngine.com/blog/2019/6/6/tethered-minimization-of-small-molecules-with-rdkit-towards-tethered-docking-on-proteins-with-rdock (2019).
7. PyMOL. The PyMOL Molecular Graphics System, Version 3.0 Schrödinger, LLC. (2024)
