# Supplementary material for: An integrated experimental and computational pipeline for crystallographic fragment screening of membrane protein in the lipid cubic phase
Source: Commun Chem. 2026 May 13;9:257. doi: 10.1038/s42004-026-02059-7 (PMC13402683; doi:10.1038/s42004-026-02059-7)
Supplement: Supplementary file 3 — Description of Additional Supplementary Files [file 42004_2026_2059_MOESM3_ESM.pdf]

Description of Additional Supplementary Files:

File name: Supplementary Data 1

Description: SMILES codes of initially selected fragments

File name: Supplementary Data 2

Description: Data collection and refinement statistics of the serial DMSO testing and ZM241385 replacement of theophylline at the thermostabilized adenosine A2A receptor extracellular site

File name: Supplementary Data 3

Description: Data collection and refinement statistics of the initial hits for the thermostabilized adenosine A2A receptor

File name: Supplementary Data 4

Description: Data collection and refinement statistics for follow-up compound/fragment hits on the thermostabilized adenosine A2A receptor

File name: Supplementary Data 5

Description: Summary of SMILES codes, X-ray hits, affinity assay results, and GPCRdb similarity for the fragments/compounds used in this study

File name: Supplementary Data 6

Description: GCI waveRAPID analysis of fragments positive in both GCI and X-ray screening, with MCK analysis for selected fragments

File name: Supplementary Data 7

Description: GCI waveRAPID analysis of fragments positive in GCI but negative in X-ray screening
