# Supplementary material for: An integrated experimental and computational pipeline for crystallographic fragment screening of membrane protein in the lipid cubic phase
Source: Commun Chem. 2026 May 13;9:257. doi: 10.1038/s42004-026-02059-7 (PMC13402683; doi:10.1038/s42004-026-02059-7)
Supplement: Supplementary file 11 — Life sciences reporting summary [file 42004_2026_2059_MOESM11_ESM.pdf]

nature portfolio

Corresponding author(s): Chia-Ying Huang

Last updated by author(s): Apr 22, 2026

Reporting Summary

Nature Portfolio wishes to improve the reproducibility of the work that we publish. This form provides structure for consistency and transparency in reporting. For further information on Nature Portfolio policies, see our Editorial Policies and the Editorial Policy Checklist.

Statistics

For all statistical analyses, confirm that the following items are present in the figure legend, table legend, main text, or Methods section.

n/a	Confirmed	
☒	☐	The exact sample size (*n*) for each experimental group/condition, given as a discrete number and unit of measurement
☒	☐	A statement on whether measurements were taken from distinct samples or whether the same sample was measured repeatedly
☒	☐	The statistical test(s) used AND whether they are one- or two-sided<br>*Only common tests should be described solely by name; describe more complex techniques in the Methods section.*
☒	☐	A description of all covariates tested
☒	☐	A description of any assumptions or corrections, such as tests of normality and adjustment for multiple comparisons
☒	☐	A full description of the statistical parameters including central tendency (e.g. means) or other basic estimates (e.g. regression coefficient) AND variation (e.g. standard deviation) or associated estimates of uncertainty (e.g. confidence intervals)
☒	☐	For null hypothesis testing, the test statistic (e.g. *F*, *t*, *r*) with confidence intervals, effect sizes, degrees of freedom and *P* value noted<br>*Give P values as exact values whenever suitable.*
☒	☐	For Bayesian analysis, information on the choice of priors and Markov chain Monte Carlo settings
☒	☐	For hierarchical and complex designs, identification of the appropriate level for tests and full reporting of outcomes
☒	☐	Estimates of effect sizes (e.g. Cohen's *d*, Pearson's *r*), indicating how they were calculated

Our web collection on statistics for biologists contains articles on many of the points above.

Software and code

Policy information about availability of computer code

Data collection	Data collection was done at the synchrotron beamlines at the SLS and ESRF. All necessary software is made available after a successful beam time proposal.
Data analysis	XDS (20230630), XSCALE, autoPROC STARANISO (1.1.7), DIMPLE (2.6.2), Coot (0.9.8.96), REFMAC (5.8.0267), PanDDA analysis (0.2.14), BUSTER (2.10.4) Phenix (1.20_4459), PyMOL (3.1.0), RDKit (2023.09.2), rDock, DrugScoreX, WAVEcontrol software Datawarier, PubChem Sketcher V2.4

For manuscripts utilizing custom algorithms or software that are central to the research but not yet described in published literature, software must be made available to editors and reviewers. We strongly encourage code deposition in a community repository (e.g. GitHub). See the Nature Portfolio guidelines for submitting code & software for further information.

Data

Policy information about availability of data

All manuscripts must include a data availability statement. This statement should provide the following information, where applicable:

- Accession codes, unique identifiers, or web links for publicly available datasets
- A description of any restrictions on data availability
- For clinical datasets or third party data, please ensure that the statement adheres to our policy

The structure and structure factors for A2A receptor complexes with fragments have been deposited in the Protein Data Bank under the accession codes listed

below. The associated statistics are provided in Supplementary Data S2–S4.
7IN5, 7IN6, 7IN7, 7IN8, 7IN1, 7IN2, 7IN3, 7IN4, 7IN9, 7INA, 7INB, 7INC, 7IND, 7INE, 7INF, 7ING, 7INH, 7INI, 7INJ, 7INK, 7INL, 7INM, 7INN, 7INO, 7INP, 7INQ, 7INR, 7INT, 7INU, 7INV, 7INW, 7INX, 7INY, 7INZ, 7IO0, 7IO1, 7IO2, 7IO3, 7IO4, 7IO5, 7IO6, 7IO7, 7IO8, 7IO9, 7IOA, 7IOB, 7IOC, 7IOD, 7IOE, 7IOF, 7IOG, 7IOH, 7IOI, 7IOJ, 7IOK, 7IOL, 7IOM, 7ION, 7IOO, 7IOP, 7IOQ, 7IOR, 7IOS, 7IOT, 7IOU, 7IOV, 7IOW, 7IOX, 7IOY, 7IOZ, 7IP0, 7IP1, 7IP2, 7IP3, 7IP4, 7IP5, 7IP6, 7IP7, 7IP8, 7IP9, 7IPA, 7IPB, 7IPC, 7IPD, 7IPE, 7IPF, 7IPG
All relevant data supporting the findings of this study are available from the corresponding author, Chia-Ying Huang (chia-ying.huang@psi.ch), upon reasonable request.

Research involving human participants, their data, or biological material

Policy information about studies with human participants or human data. See also policy information about sex, gender (identity/presentation), and sexual orientation and race, ethnicity and racism.

Reporting on sex and gender	not involved
Reporting on race, ethnicity, or other socially relevant groupings	not involved
Population characteristics	not involved
Recruitment	not involved
Ethics oversight	not involved

Note that full information on the approval of the study protocol must also be provided in the manuscript.

Field-specific reporting

Please select the one below that is the best fit for your research. If you are not sure, read the appropriate sections before making your selection.

☒ Life sciences ☐ Behavioural & social sciences ☐ Ecological, evolutionary & environmental sciences

For a reference copy of the document with all sections, see nature.com/documents/nr-reporting-summary-flat.pdf

Life sciences study design

All studies must disclose on these points even when the disclosure is negative.

Sample size	The selection of X-ray crystallography datasets was based on standard quality metrics, including I/σ(I), CC1/2, and completeness.
Data exclusions	no data were excluded form the analysis
Replication	All crystallographic data were collected from single crystals. No replicate crystallographic measurements were performed.
Randomization	This study focuses on the development of a crystallographic platform and the analysis of protein–ligand structures; therefore, randomization is not applicable.
Blinding	This study focuses on the development of a crystallographic platform and the analysis of protein–ligand structures; therefore, blinding is not applicable.

Reporting for specific materials, systems and methods

We require information from authors about some types of materials, experimental systems and methods used in many studies. Here, indicate whether each material, system or method listed is relevant to your study. If you are not sure if a list item applies to your research, read the appropriate section before selecting a response.

Materials & experimental systems

n/a	Involved in the study	
☒	☐	Antibodies
☐	☒	Eukaryotic cell lines
☒	☐	Palaeontology and archaeology
☒	☐	Animals and other organisms
☒	☐	Clinical data
☒	☐	Dual use research of concern
☒	☐	Plants

Methods

n/a	Involved in the study	
☒	☐	ChIP-seq
☒	☐	Flow cytometry
☒	☐	MRI-based neuroimaging

Eukaryotic cell lines

Policy information about cell lines and Sex and Gender in Research

Cell line source(s)	Hi5 expression cell lines were purchased
Authentication	This is used for protein expression . No authentication was necessaary.
Mycoplasma contamination	no contaminations were detected in this study.
Commonly misidentified lines (See ICLAC register)	no commonly misidentified lines were used.

Plants

Seed stocks	*Report on the source of all seed stocks or other plant material used. If applicable, state the seed stock centre and catalogue number. If plant specimens were collected from the field, describe the collection location, date and sampling procedures.*
Novel plant genotypes	*Describe the methods by which all novel plant genotypes were produced. This includes those generated by transgenic approaches, gene editing, chemical/radiation-based mutagenesis and hybridization. For transgenic lines, describe the transformation method, the number of independent lines analyzed and the generation upon which experiments were performed. For gene-edited lines, describe the editor used, the endogenous sequence targeted for editing, the targeting guide RNA sequence (if applicable) and how the editor was applied.*
Authentication	*Describe any authentication procedures for each seed stock used or novel genotype generated. Describe any experiments used to assess the effect of a mutation and, where applicable, how potential secondary effects (e.g. second site T-DNA insertions, mosiacism, off-target gene editing) were examined.*
