# Supplementary material for: An integrated experimental and computational pipeline for crystallographic fragment screening of membrane protein in the lipid cubic phase
Source: Commun Chem. 2026 May 13;9:257. doi: 10.1038/s42004-026-02059-7 (PMC13402683; doi:10.1038/s42004-026-02059-7)
Supplement: Supplementary file 1 — Transparent Peer Review file [file 42004_2026_2059_MOESM1_ESM.pdf]

nature portfolio

Peer Review File

An Integrated Experimental and Computational Pipeline for Crystallographic Fragment Screening of Membrane Protein in the Lipid Cubic Phase

Corresponding Author: Dr Chia-Ying Huang

This file contains all reviewer reports in order by version, followed by all author rebuttals in order by version.

Version 0:

Reviewer comments:

Reviewer #1

(Remarks to the Author)
This paper is well written and clearly describes an interesting approach for discovery of hits against challenging targets. The use of biosensors for validation is good, but there is no data presented from these experiments, they are only described in the text. It would be important to see the quality of the data and how the hits were discriminated from the non-hits. Also controls should be shown.

The text describing the GCI experiments is misleading. On row 258 it says that the 109 compounds were injected in parallell, but I assume they mean that they were injected one at a time over three protein surfaces and a blank surface, in parallel.

Please see the guidelines for publishing biosensor data for inspiration of how to present data in a meaningful way:
Belcher PE, Moberg A, Murphy MB. Standards for reporting optical biosensor experiments (STROBE): Improving standards in the reporting of optical biosensor-based data in the literature. SLAS Discov. 2024 Dec;29(8):100192. doi: 10.1016/j.slasd.2024.100192.

Reviewer #2

(Remarks to the Author)
This manuscript by Huang et al. presents a significant methodological advancement in the field of fragment-based drug discovery (FBDD) for membrane proteins. The authors have developed an integrated pipeline for lipidic cubic phase crystallographic fragment screening (LCP-CFS), that enables high-throughput ligand screening with LCP-grown crystals using a mutated Adenosine A2A receptor, a member of the the G-protein coupled receptor (GPCRs) for method validation. This is a notoriously challenging area due to the difficulties associated with handling and screening membrane proteins in LCP. A library of 568 fragments was screened, identifying 23 initial hits in two distinct binding sites. 109 follow up molecules were selected of which 19 showed binding both by crystallography and grating-coupled interferometry (GCI). The results convincingly demonstrate the potential of this approach, even if the biological relevance of the extracellular binding site pursued here is not clearly established and the quality of the electron density maps for some of the ligands is poor. The manuscript is well-written, and concise and the authors are transparent about the potential limitations of their findings, particularly regarding the influence of stabilizing mutations on the intracellular site. This study will be of interest to the structural biology and medicinal chemistry communities focused on challenging membrane protein targets.

Comments
The manuscript is of high quality and requires only minor revisions.

- The authors acknowledge that GCI data allows the acquisition of affinity data (line 262). However, this data is not presented. Please, include affinity data. Are the 19 hits identified both through crystallography and CGI showing higher affinities than the rest ? Adding to this, while GCI confirms binding, it does not definitively prove that binding occurs at the exact same intracellular site identified crystallographically. The authors should briefly acknowledge this nuance in the

discussion. This could be particularly relevant for the hits showing weaker correlation wit the electron density.

- The adenosine A_2A receptor (A_2AR) is indeed a "gold standard" model system in the field of GPCR structural biology, largely because it is exceptionally well-behaved compared to most other membrane proteins. Currently, the manuscript successfully presents the LCP-CFS pipeline as a powerful tool but does not sufficiently address that its applicability is contingent on solving the primary challenge of membrane protein structural biology: obtaining large amounts of robust crystals. For readers not intimately familiar with GPCR crystallography, the relative ease with which the system behaves might be missed. To strengthen the manuscript and provide a more balanced perspective, we suggest outlining in the discussion the ideal characteristics of a target protein suitable for this pipeline.

- In lines 95-96 the authors note LCP transitions to a "liquid-like sponge phase" after 3 hours across all tested DMSO concentrations. Is this a significant outcome that should be pursued in this method? Was a control experiment performed to determine if the phase transition is induced by DMSO alone? The authors could give their opinion on the implications, which could be of help for those attempting to reproduce the method. Does this sponge phase potentially be indicative of fragment binding ? or could it enhance fragment soaking by increasing the diffusion rate, representing a beneficial feature of the optimized conditions?

- In Lines 125-126 the authors mention "partially bound 9.9 MAG" and it use to optimize soaking protocol. 9.9 MAG (1-oleoyl-rac-glycerol) is a component of the LCP matrix and is known to produce density that can be mistaken for ligands. The authors should provide more detail on how their manual inspection reliably distinguished MAG density from a true fragment hit. For example, were there characteristic features of the MAG density (e.g., shape, location, connection to the lipid environment) that were used as exclusion criteria? Providing a supplementary figure showing an example of this ambiguity would be highly instructive.

- Lines 170-173 The authors re-inspected maps at lower sigma cut-offs (0.7-0.8 for 2Fo-Fc, 2.5-2.8 for Fo-Fc) to find 17 additional hits. What additional validation was performed on these hits to ensure they were not false positives? Overall, visual inspection of the maps for numerous hits is not convincing. Based on the figures provided (Figures 3, S6, and S9), several maps—such as, F99-31 (fig3), FU163-106 (fig3), FU163-109 (fig3), FU336-32 (fig3), F132 (figS6), F2 (figS6), F4 (figS6), F7 (figS6), F12 (figS6), F44 (figS6), F148 (figS6), F163 (figS6), F235 (figS6), F242 (figS6), F273 (figS6), F293 (figS6), F336 (figS6), F379 (figS6), FU2-57 (figS9), FU4-54 (figS9), FU-40 (figS9), FU7-15 (figS9), FU12-19 (figS9), FU44-18 (figS9), FU99-31 (figS9), FU99-91 (figS9), FU163-109 (figS9), FU210-12 (figS9), FU223-43 (figS9), FU235-66 (figS9), FU273-23 (figS9), FU27377 (figS9), FU300-24 (figS9), FU336-32 (figS9), FU428-8 (figS9), FU426-36 (figS9) —appear ambiguous and may be subject to interpretation. Please, include additional data that could help interpretation, like real-space correlation coefficient (RSCC) and B factors for ligand atoms only, 2Fo-Fc maps ....

- The reported CC1/2 value for the outer resolution shell in PDB entry 7IOY is 0.98 (0.09) (Table, page 33). It is unclear whether the value of 0.09 represents the true data cut-off, as it appears unusually low. Were the resulting ligand models chemically plausible and well-refined (e.g., stable B-factors, satisfactory real-space correlation coefficients)? The manuscript should more robustly substantiate the validity of hits identified under these less stringent map thresholds to strengthen confidence in the reported findings See also point above.

- It would be helpful to present representative refinement statistics (R-factors, Ligand RSCC …) for the full fragment screening dataset, either in Figure S5 or as a separate figure.

Other Comments
- In the abstract, the phrase "the identification of a novel intracellular ligand-binding site in a GPCR …" could be moderated as the physiological role of this site and its relevance for drug design are yet to be established. For example “ numerous ligands were found at a so far uncharacterized intracellular pocket…”

- The hits for the well-established extracellular binding site have not been pursued ? Why ? It would have seem logical to focus follow-up efforts on that site.

- LCP-CFS pipeline is undoubtedly effective. However, the negative comments concerning the previous work by the Marquez group at EMBL (Healey et al. ref 29) “ lack of reproducibility of vapour diffusion” and “limited access” don't seem justified. Vapour diffusion plates are used in the present work as well and the CrystalDirect technology is available both commercially and through user facilities at two synchrotrons sites. Healey et al. did also demonstrate soaking of LCP crystals, which is not mentioned here.

- The manuscript claims 10% DMSO "yielded the best electron density map quality for the ligand." How was "best quality" determined? This subjective assessment should be supported by quantitative data. Please provide metrics such as the refined B-factor, occupancy, and/or real-space correlation coefficient (RSCC) for the ligand under the different conditions tested.

Reviewer #3

(Remarks to the Author)
The manuscript presents a pipeline for fragment screening of GPCR crystals grown in LCP. The pipeline was applied to a

thermostabilized adenosine A_{2A} receptor to screen 568 fragments, identifying 23 initial hits. Further optimization of these hits led to the design of 109 follow-up compounds, 56 of which yielded crystal structures. This is an impressive work by a team from the Paul-Scherrer Institute in collaboration with the company LeadXpro. Fragment screening has been widely used with crystals of soluble proteins, however, its application here to membrane proteins crystallized in LCP is novel. Importantly, this screening identified a novel allosteric site at the intracellular side of the receptor and discovered several compounds binding to this site. The caveat is that the receptor had to be heavily thermostabilized and an orthosteric ligand was required for crystallization, limiting the applicability for screening fragments binding in the orthosteric site by this method. Nevertheless, the work should attract attention of structural biologists and medicinal chemists, however, the authors should address specific comments below before this manuscript can be recommended for publication.

Lines 84-88. Please describe the abbreviation tsA_{2A}R and explain that the receptor was crystallized in the presence of 5 mM of theophylline in the first paragraph of the Results section.

Line 86. The main text mentions that crystallization buffer without theophylline was added to each well, however, in the Methods (Line 430) the crystallization buffer added to each well contains theophylline. Which one is correct?

Lines 89-108. According to Figure S2, the LCP transforms into a sponge phase after about 3 h of incubation in the SwissCI-3-lens plate even without ligand and DMSO added, meaning that the phase change occurs mostly due to dehydration rather than DMSO or ligand addition. Although tsA_{2A}R crystals apparently survive the phase change, such change in conditions may be incompatible with crystals of other membrane proteins. This would be an additional limitation of this method.

Lines 89-108. The ligand soaking method was developed using the high affinity antagonist ZM241385 with Ki~1 nM. Of course, none of the screened fragments is expected to bind with such high potency. Therefore, the soaking time and concentrations optimized for ZM241385 are unlikely to be optimal for most low potency fragments. It would be helpful if the authors re-assess the soaking time vs concentration using a low affinity antagonist with Ki>1 µM.

Lines 257-269. Please provide complete results for the GCI assays, including raw curves, association (Ka) and dissociation (Kd) rates and Rmax values for the screened 109 compounds, as well as the dose-rate results and measured affinities for the 19 compounds identified as hits in both X-ray and GCI assays. Which His tag and at which terminus was used for capturing purified receptors on a PCP-NTA chip for the GCI assays? Additionally, it would be important to evaluate the allosteric effect of the most potent compounds in cell signaling assays. Do they act as negative allosteric modulators?

Line 259. Does the wtA_{2A}R contain bRIL fusion? This should be clarified, and perhaps it would be more appropriate to use wtA_{2A}R-bRIL abbreviation instead of wtA_{2A}R. The tsGPR17 construct should also be described.

Line 428. SwissCI-3-lens plates are offered in three different materials: PS, UVP, and UVXPO. Each of these polymers display different birefringent background under cross-polarizers. Please, specify, which exactly plates were used.

Line 446. The authors mention that “fragment-soaked crystals were harvested after either 3 or 21 hours”. This is a large difference. The actual soaking time for each fragment should be indicated in Table S4.

Lines 462-474. Some X-ray data were collected at SLS and some at ESRF. Please, indicate explicitly which datasets were collected at each of the synchrotron source.

Line 476. STARANISO was used to apply anisotropic truncation to X-ray data. Please indicate resolution limits applied by STARANISO for each dataset in Tables S1-S3.

Lines 480-481. Phenix, BUSTER, and REFMAC were used for refinement. Please, include the versions of the refinement programs and indicate the reason why different programs were used. It is difficult to compare statistics when different datasets are refined by different programs.

Tables S1-S3. The overall completeness of the fragment-soaked datasets ranges from 42% (FU35-2) to 88.4% (F99) with majority around 70%. The completeness of the ZM241385-soaked data in Table S1 is much higher (>99%). What is the reason for such low completeness of the fragment-soaked data, and how confident the authors are in the Fo-Fc densities for such datasets? Do the Fo-Fc densities shown in Figures 3, S6, S9 use missing Fo filled with Fc? If yes, how do the densities change if filling is not used?

Tables S1-S3. The Rmeas values are relatively high, exceeding 30% for many datasets and even 70% for some. It is hard to believe that such data can produce reliable density for the fragments. Wilson-B is also suspicious in several datasets, such as 9.48 (FU132-4) and 11.08 (FU35-47).
Table S4. Please include molecular weights for all the fragments. Also please provide a list of all 568 fragments used in this work.

Supplementary Method. The authors describe a pipeline implemented in Python 3.11.6. They should provide the code for it in github.

Reviewer #4

(Remarks to the Author)
I co-reviewed this manuscript with one of the reviewers who provided the listed reports. This is part of the Communications Chemistry initiative to facilitate training in peer review and to provide appropriate recognition for Early Career Researchers who co-review manuscripts.

Version 1:

Reviewer comments:

Reviewer #1

(Remarks to the Author)
The authors have added exemplary original data to illustrate the results from the GCI biosensor analysis. However, even with the additional data, necessary information is still missing. Clearly the authors have a challenge in using it for membrane bound receptors and they correctly state that it is not realistic to expect the estimation of kinetic parameters or affinities. So, this is not what is asked for. Nevertheless, since they state that they have integrated GCI biosensor analysis in their fragment screening pipeline, it is therefore important to understand how this method contributed to identifying and validating the hits. Without describing the method and criteria used for identifying hits and discriminating them from non-hits, this is not possible. This should be seen in the light of GCI biosensor technology being relatively new and the interpretation of the data is difficult. The reader needs to have access to the complete dataset and be guided in interpreting it. If the authors believe that there is relevant information on the procedure (screening criteria and data etc) already available in the literature, it should be referenced so the reader can find it.

Specific points:
• GCI biosensor data is only shown for 5 hits although 19 out of the 56 compounds that yielded crystal structures were stated to be validated by GCI analyses. Data for all these compounds should be shown so it is possible to see that they fulfil criteria used to identify them as hits.

• Figure S11.
o The criteria used to confirm binding of the hits to the target by the GCI method is described in the Methods section, but a combination of a variety of criteria appears to be considered. The way this is implemented if not shown so the reader can understand why the data shown has been interpreted to represent hits. What about the other 14 hits?
o The font size for the units on the y-axis is too small to be read. (The units can be explained in the legend.) What was the rationale for setting the confidence to high for signals between 1 and 10 pg/mm²?
o The dilution series from 200 uM top concentration is not specified.
o The data for the two controls with adenosine and NECA that have been included, as requested. But they are not commented at all, so it is not possible to understand in what way these compounds serve as controls. What information is extracted from these control measurements?
o It is not a 1:1 interaction model that is shown in black, but most likely theoretical traces obtained by regression analysis using a 1:1 model.

•Figure S12.
o The font size in the figures is too small to be legible.
o It is not a 1:1 interaction model that is shown in black, but most likely theoretical traces obtained by regression analysis using a 1:1 model.
o It appears that different KD and Rmax values are estimated in the global fit and the steady-state analysis. This should be commented.
o There is no explanation to how the data extracted from this figure is interpreted.

Reviewer #2

(Remarks to the Author)
The authors have satisfactorily addressed this reviewer's concerns with this version of the MS.

Reviewer #3

(Remarks to the Author)
The authors have addressed most of the critiques and improved the manuscript, however, several questions remain unresolved. My additional comments are provided below in bold.

Lines 84-88. Please describe the abbreviation tsA2AR and explain that the receptor was crystallized in the presence of 5 mM of theophylline in the first paragraph of the Results section.

→ We thank the reviewer for this comment. A description for the tsA2AR has been added to the line 84 "(human adenosine A2A receptor with A277S revert mutation, A2AR-StaR2-bRIL-A277S, hereafter referred to as tsA2AR, crystals were grown in the present of theophylline, details in method section)"

Please, add thermostabilized – ("human thermostabilized adenosine A2A …)

Line 86. The main text mentions that crystallization buffer without theophylline was added to each well, however, in the Methods (Line 430) the crystallization buffer added to each well contains theophylline. Which one is correct?

→ *Thank the reviewer for pointing out this. The method (line 430) has been corrected. We removed "5 mM theophyline" in the description and change the "crystallization solution" to "reservoir solution".*

Ok. Theophylline is misspelled.

Lines 89-108. According to Figure S2, the LCP transforms into a sponge phase after about 3 h of incubation in the SwissCI-3-lens plate even without ligand and DMSO added, meaning that the phase change occurs mostly due to dehydration rather than DMSO or ligand addition. Although tsA2AR crystals apparently survive the phase change, such change in conditions may be incompatible with crystals of other membrane proteins. This would be an additional limitation of this method.

→ *We are aware that the phase transition could potentially have negative effects on certain LCP-grown crystals. For this reason, we optimized the DMSO concentration and soaking time to minimize adverse impacts during soaking. In the case of tsA2A receptor crystals, we observed that the LCP spontaneously transforms into the sponge phase after several hours of incubation, even without DMSO or ligand addition, yet the diffraction quality remains unaffected. This observation is consistent with previous reports that tsA2A crystals can tolerate such transitions.*
Importantly, the sponge phase, characterized by enlarged aqueous channels, may actually facilitate fragment diffusion and thus improve soaking efficiency in tsA2A crystals. Nevertheless, we acknowledge that for other membrane proteins, especially those sensitive to dehydration or phase instability, such transitions could indeed pose limitations. To minimize this risk, we recommend controlling humidity and reducing the transfer time when moving LCP from the syringe to the plate. Alternatively, using higher fragment concentrations can improve diffusion efficiency, allowing soaking to be completed before the phase transition occurs.

Please, add these recommendations to the manuscript.

Lines 89-108. The ligand soaking method was developed using the high affinity antagonist ZM241385 with Ki~1 nM. Of course, none of the screened fragments is expected to bind with such high potency. Therefore, the soaking time and concentrations optimized for ZM241385 are unlikely to be optimal for most low potency fragments. It would be helpful if the authors re-assess the soaking time vs concentration using a low affinity antagonist with Ki>1 µM.

→ *We thank the reviewer for this helpful comment. Our initial objective was to confirm that the LCP-CFS soaking method functioned reliably, so we used the high-affinity antagonist ZM241385 to validate ligand uptake clearly and to establish basic soaking parameters. This initial test also successfully produced fragment hits, indicating that the optimized conditions are suitable even for weak binders.*
We agree that a low-affinity antagonist (Ki > 1 µM) would better represent fragment behavior. However, due to time constraints and the limited availability of suitable weak binders during the experimental phase, we proceeded with ZM241385 for method development. We will consider further optimization with a representative weak binder in future work.

Please, acknowledge this limitation in the manuscript.

Lines 257-269. Please provide complete results for the GCI assays, including raw curves, association (Ka) and dissociation (Kd) rates and Rmax values for the screened 109 compounds, as well as the dose-rate results and measured affinities for the 19 compounds identified as hits in both X-ray and GCI assays. Which His tag and at which terminus was used for capturing purified receptors on a PCP-NTA chip for the GCI assays? Additionally, it would be important to evaluate the allosteric effect of the most potent compounds in cell signaling assays. Do they act as negative allosteric modulators?

→ *Please see our responses to Reviewer 1 above.*
→ *The target proteins used contain a c-terminal 10x Histidine tag for capturing on the Ni-NTA PCP sensor surface. This has been clarified in the GCI method section.*
→ *Biological signaling assays for testing potential cellular effects of the identified compounds have not yet been conducted.*

C-terminal 10x His-tag is described for GPR17 but no description is given for tsA_{2A} and wtA_{2A}.

Line 259. Does the wtA2AR contain bRIL fusion? This should be clarified, and perhaps it would be more appropriate to use wtA2AR-bRIL abbreviation instead of wtA2AR. The tsGPR17 construct should also be described.

→ *We thank the reviewer for this comment. Both wtA2AR and tsA2AR constructs used in this study contain a bRIL fusion; this has now been clarified in the manuscript. The GPR17 construct used as a negative control on GCI corresponds to residues 1-342 of human GPR17. A HA signal sequence and FLAG peptide were inserted at the N-terminus of the protein. The C-terminus was truncated at residue 342 and replaced with a 3C cleavage site, followed by a 10x His, TEV cleavage site and enhanced green fluorescence protein (eGFP) to facilitate purification. To enhance protein's thermal-stability, 7 mutations (D69V, C108Y, A155L, S315V, S107L, F158Y and T230L) were introduced to the wild type sequence.*
This has also been added to the corresponding method part.

ok

Line 428. SwissCI-3-lens plates are offered in three different materials: PS, UVP, and UVXPO. Each of these polymers display different birefringent background under cross-polarizers. Please, specify, which exactly plates were used.

→ We thank the reviewer for pointing this out. All experiments reported in this study were performed using SwissCI-3-lens UVXPO plates. This plate type exhibits low birefringence and is therefore well suited for observing LCP crystals under cross-polarized light. This information has now been added to the Methods section.

ok

Line 446. The authors mention that "fragment-soaked crystals were harvested after either 3 or 21 hours". This is a large difference. The actual soaking time for each fragment should be indicated in Table S4.

→ We thank the reviewer for pointing this out. All samples listed in Table S4 correspond to tsA2A LCP crystals soaked with fragments for 3 hours. We have added a note at the end of Table S4 to clarify the soaking time. This duration was chosen because the fragment binding efficiency, as indicated by the electron density maps and quantitative statistics (Fig. S4), reached sufficient occupancy within 3 hours.

Sorry, I do not see a note about soaking time in Table S4?

Lines 462-474. Some X-ray data were collected at SLS and some at ESRF. Please, indicate explicitly which datasets were collected at each of the synchrotron source.

→ We thank the reviewer for pointing this out. The initial screening of 568 fragments and the DMSO optimization with tsA2A LCP crystals were performed at the SLS, while the subsequent soaking (follow-up) of 109 fragments with tsA2A LCP crystals was carried out at ESRF. We have added this information to the Methods section as well as to Tables S1, S2, and S3.

ok

Line 476. STARANISO was used to apply anisotropic truncation to X-ray data. Please indicate resolution limits applied by STARANISO for each dataset in Tables S1-S3.

→ We thank the reviewer for this comment. The datasets listed in Table S1 were processed using XDS, whereas all fragment datasets in Tables S2 and S3 were processed using autoPROC with the STARANISO option. This distinction and the corresponding anisotropic resolution limits determined by STARANISO have now been clarified and included in Tables S1–S3.

I do not see anisotropic resolution limits in Tables S1-S3. In table S3, the resolution range for the highest resolution shell and the total resolution range are switched (the highest resolution shell should be in parentheses).

Lines 480-481. Phenix, BUSTER, and REFMAC were used for refinement. Please, include the versions of the refinement programs and indicate the reason why different programs were used. It is difficult to compare statistics when different datasets are refined by different programs.

→ We thank the reviewer for pointing this out. REFMAC (5.8.0267) was used during PanDDA analysis, and BUSTER (2.10.4) was applied to refine ambiguous hits. However, all final fragment hits were ultimately refined using Phenix (1.20_4459) to ensure consistency in the reported statistics. We have added this information to the Methods section for clarification.

ok

Tables S1-S3. The overall completeness of the fragment-soaked datasets ranges from 42% (FU35-2) to 88.4% (F99) with majority around 70%. The completeness of the ZM241385-soaked data in Table S1 is much higher (>99%). What is the reason for such low completeness of the fragment-soaked data, and how confident the authors are in the Fo-Fc densities for such datasets? Do the Fo-Fc densities shown in Figures 3, S6, S9 use missing Fo filled with Fc? If yes, how do the densities change if filling is not used?

→ We thank the reviewer for the comment. The dataset for ZM241385 was processed using XDS, as this ligand is a well-characterized high-affinity A2A binder and the dataset could be reliably handled with a straightforward processing approach. All fragment datasets, however, were processed with autoPROC with the default STARANISO option to ensure that all usable reflections were retained.
All the Fo-Fc maps shown (green mesh) were generated by DIMPLE after removing the ligand. Missing reflections in the input data were not filled with Fc; the maps therefore reflect only the experimentally observed structure factors. We note that the fragment densities remain clearly visible even with this conservative calculation.

Please, include the information about the Fo-Fc maps generation and Fc filling in Methods.

Tables S1-S3. The Rmeas values are relatively high, exceeding 30% for many datasets and even 70% for some. It is hard to

believe that such data can produce reliable density for the fragments. Wilson-B is also suspicious in several datasets, such as 9.48 (FU132-4) and 11.08 (FU35-47).

→We thank the reviewer for raising this important point regarding the data quality. We acknowledge that the Rmeas values of some datasets appear relatively high. This is mainly due to the weak and anisotropic diffraction commonly observed in GPCR–LCP crystals, especially for small-fragment soaks. Notably, despite the elevated Rmeas, the electron density maps for these datasets remain interpretable, and the fragment-binding features are clearly supported by both 2Fo–Fc and Fo–Fc maps. We also note that all datasets were processed using autoPROC with the default STARANISO option, and no processing parameters were manually restricted, which may explain the higher global statistics.
We agree that the Wilson B-values of FU132-4 and FU35-47 (9.48 and 11.08 Å²) are unusually low. Diffraction data were processed using autoPROC with anisotropic correction applied by STARANISO. Anisotropic truncation alters the distribution of reflection intensities by selectively retaining high-resolution data along stronger reciprocal-space directions while removing weak reflections in others. As a consequence, the assumptions underlying the Wilson plot, which require isotropic and uniformly sampled data, are no longer strictly valid. Wilson B-factors calculated after STARANISO correction are therefore systematically underestimated.
However, both crystals contain strong and well-defined electron density (Fig. S10), and the refined atomic B-factors are consistent with well-ordered ligand–protein complexes, indicating that the underlying diffraction data are not problematic. For completeness, we have included the refined B-factors of individual fragments in Tables S2 and S3, and the corresponding RSCC values are provided in Figs. S7 and S10.

ok

Table S4. Please include molecular weights for all the fragments. Also please provide a list of all 568 fragments used in this work.

→ We thank the reviewer for this suggestion. The molecular weights of all hit fragments have now been added to Table S4. We would be happy to provide a complete list of all 568 fragments used in this work upon request.

A complete list could be easily included as a supplemental Excel file.

Supplementary Method. The authors describe a pipeline implemented in Python 3.11.6. They should provide the code for it in github.

→ We thank the reviewer for this suggestion. The Python-based pipeline described in the Supplementary Methods is a workflow tailored to our local setup rather than a general software tool. The underlying code is not in a publishable form—it consists of multiple interconnected scripts tied to our in-house infrastructure, vendor APIs requiring private access keys, and interactive third-party software. Preparing it for public release would require substantial redevelopment.
However, the workflow is described in sufficient detail for others to conceptually reproduce the approach, and we note that a more robust and portable version may be developed and published separately in the future.

This is unfortunate, as this part of the workflow could not be exactly reproduced by other researchers.

Reviewer #4

(Remarks to the Author)
I co-reviewed this manuscript with one of the reviewers who provided the listed reports. This is part of the Communications Chemistry initiative to facilitate training in peer review and to provide appropriate recognition for Early Career Researchers who co-review manuscripts.

Version 2:

Reviewer comments:

Reviewer #1

(Remarks to the Author)
The revised manuscript has addressed the concerns I had with the last version of the manuscript. There are a few minor points that remain:

1. What is meant by “fully bound” and “partially bound” fragments?
2. The figures in Figure S11 have axis text and text inside the panels that cannot be read. Pleas make these figures larger or provide the information by some other means.
3. Page 8: It is stated on row 8 that “ZM241385 blocked the orthosteric site and showed no binding (Fig. S11).” Please explain, it is not logical. If there was a pre-incubation of the surface with ZM241385, it could be possible, but the experiment should be explained, if so and the sentence rephrased.

Reviewer #3

(Remarks to the Author)
The authors have addressed my comments satisfactory and I'm happy to recommend this manuscript for publication. One small typo in Supplementary Fig. S4, the bottom right section should likely be labeled with 10 μM ZM241385 instead of 5 μM.

Open Access This Peer Review File is licensed under a Creative Commons Attribution 4.0 International License, which permits use, sharing, adaptation, distribution and reproduction in any medium or format, as long as you give appropriate credit to the original author(s) and the source, provide a link to the Creative Commons license, and indicate if changes were made.
In cases where reviewers are anonymous, credit should be given to 'Anonymous Referee' and the source.
The images or other third party material in this Peer Review File are included in the article's Creative Commons license, unless indicated otherwise in a credit line to the material. If material is not included in the article's Creative Commons license and your intended use is not permitted by statutory regulation or exceeds the permitted use, you will need to obtain permission directly from the copyright holder.
To view a copy of this license, visit https://creativecommons.org/licenses/by/4.0/

Point-by-point response revision 1

Reviewer #1 (Remarks to the Author):

This paper is well written and clearly describes an interesting approach for discovery of hits against challenging targets. The use of biosensors for validation is good, but there is no data presented from these experiments, they are only described in the text. It would be important to see the quality of the data and how the hits were discriminated from the non-hits. Also controls should be shown.

→ We thank the reviewer for this comment. To address this point, we have included representative biosensor data in Figs. S11–S12, showing five positive fragments, two control samples, and one non-hit fragment to illustrate data quality and hit discrimination. While the biosensor method is, in principle, capable of providing affinity and kinetic parameters, in this study the data were used solely for binary classification of fragment binding (hit vs. non-hit). In addition, the results of the GCI interaction screening for all 109 follow-up candidates are summarized in Fig. S13.

The text describing the GCI experiments is misleading. On row 258 it says that the 109 compounds were injected in parallell, but I assume they mean that they were injected one at a time over three protein surfaces and a blank surface, in parallel.

→ Thank the reviewer for pointing out this. The compounds were subsequently injected, and this has been clarified in the relevant section.

Please see the guidelines for publishing biosensor data for inspiration of how to present data in a meaningful way:
Belcher PE, Moberg A, Murphy MB. Standards for reporting optical biosensor experiments (STROBE): Improving standards in the reporting of optical biosensor-based data in the literature. SLAS Discov. 2024 Dec;29(8):100192. doi: 10.1016/j.slasd.2024.100192.

→ We have included the GCI data, and please check Fig. S11-13

Reviewer #2 (Remarks to the Author):

This manuscript by Huang et al. presents a significant methodological advancement in the field of fragment-based drug discovery (FBDD) for membrane proteins. The authors have developed an integrated pipeline for lipidic cubic phase crystallographic fragment screening (LCP-CFS), that enables high-throughput ligand screening with LCP-grown crystals using a mutated Adenosine A2A receptor, a member of the the G-protein coupled receptor (GPCRs) for method validation. This is a notoriously challenging area due to the difficulties associated with handling and screening membrane proteins in LCP. A library of 568 fragments was screened, identifying 23 initial hits in two distinct binding sites. 109 follow up molecules were selected of which 19 showed binding both by crystallography and grating-coupled interferometry (GCI). The results convincingly demonstrate the potential of this approach, even if the biological relevance of the extracellular binding site pursued here is not clearly established and the quality of the electron density maps for some of the ligands is poor. The manuscript is well-written, and concise and the authors are transparent about the potential limitations of their findings, particularly regarding the influence of stabilizing mutations on the intracellular site. This study will be of interest to the structural biology and medicinal chemistry communities focused on challenging membrane protein targets.

Comments
The manuscript is of high quality and requires only minor revisions.

- The authors acknowledge that GCI data allows the acquisition of affinity data (line 262). However, this data is not presented. Please, include affinity data. Are the 19 hits identified both through crystallography and CGI showing higher affinities than the rest ? Adding to this, while GCI confirms binding, it does not definitively prove that binding occurs at the exact same intracellular site identified crystallographically. The authors should briefly acknowledge this nuance in the discussion. This could be particularly relevant for the hits showing weaker correlation wit the electron density.

→ Despite the fact that the method applied would in principle allow the acquisition of affinity and kinetic information, the data generated from these experiments were only used for binary identification of potential interacting fragments (yes/no). As outlined in the results section, this is due to the quality of the data obtained. As not at all untypical for biosensor experiments with detergent-solubilised membrane proteins and small and very weak analyses, the data show low signal amplitudes, additional sensor dynamics, multi-phasic signals, poor signal saturation and/or low signal-to-noise. Based on our strong expertise with such biosensor experiments, we do not consider the kinetic parameters from the fits as reliable and therefore suitable for ranking affinities or kinetics. As described, we are utilising the statistical confidence parameters for the 1:1 fits as calculated by the WAVEcontrol software for the identification of interactions, a common method applied for the analysis of GCI waveRAPID screening datasets.
→ Additional figures (Fig. S11-13) showing the quality of the biosensor data, controls and non-interacting fragments were added.
→ The notion about the uncertainty on the exact binding site from the GCI data has been added to the discussion.
→ Added "However, the GCI does not prove that fragments bind at the exact same intracellular site." on page 9.

- The adenosine A_2A receptor (A_2AR) is indeed a "gold standard" model system in the field of GPCR structural biology, largely because it is exceptionally well-behaved compared to most other membrane proteins. Currently, the manuscript successfully presents the LCP-CFS pipeline as a powerful tool but does not sufficiently address that its applicability is contingent on solving the primary challenge of membrane protein structural biology: obtaining large amounts of robust crystals. For readers not intimately familiar with GPCR crystallography, the relative ease with which the system behaves might be missed. To strengthen the manuscript and provide a more balanced perspective, we suggest outlining in the discussion the ideal characteristics of a target protein suitable for this pipeline.

→ We thank the reviewer for this insightful comment. We have addressed this point in the Discussion (lines 373–375, page 11) "This raises the question of which GPCRs or other membrane proteins already solved in LCP are most amenable to such studies, and conversely, which fragile targets may remain challenging for this approach." in the revision, we added the following statement to clarify the ideal characteristics of suitable targets: "The success of LCP-CFS requires the availability of a large number of crystals diffracting to 2.5 Å or better."

- In lines 95-96 the authors note LCP transitions to a "liquid-like sponge phase" after 3 hours across all tested DMSO concentrations. Is this a significant outcome that should be pursued in this method? Was a control experiment performed to determine if the phase transition is induced by DMSO alone? The authors could give their opinion on the implications, which could be of help for those attempting to reproduce the method. Does this sponge phase potentially be indicative of fragment binding ? or could it enhance fragment soaking by increasing the diffusion rate, representing a beneficial feature of the optimized conditions?

→ We thank the reviewer for this thoughtful question. The transition of LCP to the sponge phase under crystallization conditions containing MPD or high PEG concentrations is a well-known phenomenon (ref: doi:10.1107/S2053230X14026843). Although we did not perform a DMSO-only control, it is known that DMSO can affect the LCP mesophase in fragment studies, as noted in lines 52–53: "Additionally, fragment diffusion in LCP is slow, and the use of polar co-solvents like DMSO can destabilize the mesophase."

In our experiments, all LCP samples transitioned to the sponge phase after approximately 3 hours, both across all tested DMSO concentrations and in the absence of DMSO (Fig. S2), indicating that this phase change is not related to fragment binding. Such behavior has also been commonly observed for tsA$_{2A}$ receptor LCP samples, which frequently undergo a transition to the sponge phase under similar conditions.

The sponge phase, derived from the cubic phase, has lower bilayer curvature and reduced bending rigidity, which allows it to incorporate more aqueous solution (lyotrope). This property likely enhances the diffusion rate within the matrix and may thus facilitate fragment soaking. Therefore, while the phase transition itself is not indicative of binding, it could be beneficial for improving fragment diffusion and overall soaking efficiency.

- In Lines 125-126 the authors mention "partially bound 9.9 MAG" and it use to optimize soaking protocol. 9.9 MAG (1-oleoyl-rac-glycerol) is a component of the LCP matrix and is known to produce density that can be mistaken for ligands. The authors should provide more detail on how their manual inspection reliably distinguished MAG density from a true fragment hit. For example, were there characteristic features of the MAG density (e.g., shape, location, connection to the lipid environment) that were used as exclusion criteria? Providing a supplementary figure showing an example of this ambiguity would be highly instructive.

→ We appreciate the reviewer's insightful comment. In our analysis, we used the A$_{2A}$ receptor structure (PDB ID: 5IU4) as a template for model building and referenced the MAG position as a guide for interpreting the fragment-bound density. The density corresponding to 9.9 MAG was identified based on its characteristic shape and location — specifically, it surrounded the protein surface at hydrophobic regions near the bilayer interface, consistent with typical MAG positioning in LCP structures.

Although previous reports (e.g., PDB ID: 7E2Y) have shown lipid-like density extending into the intracellular pocket of Serotonin-bound Serotonin 1A (5-HT1A) receptor (DOI: https://doi.org/10.1038/s41586-021-03376-8), our structure does not display such features. The observed fragment density is spatially distinct from the MAG-associated regions and does not show continuous connection to the lipid matrix, supporting that it represents a true fragment binding event rather than residual MAG density.

We have included a supplementary figure (Fig. S6) to illustrate the typical MAG-like density and their location around the membrane protein and added an explanation in the main text as "To mitigate this, we conducted a manual inspection to exclude hydrophobic regions near the bilayer interface where MAG binds typically (Fig. S6)."

- Lines 170-173 The authors re-inspected maps at lower sigma cut-offs (0.7-0.8 for 2Fo-Fc, 2.5-2.8 for Fo-Fc) to find 17 additional hits. What additional validation was performed on these hits to ensure they were not false positives? Overall, visual inspection of the maps for numerous hits is not convincing. Based on the figures provided (Figures 3, S6, and S9), several maps—such as, F99-31 (fig3), FU163-106 (fig3), FU163-109 (fig3), FU336-32 (fig3), F132 (figS6), F2 (figS6), F4 (figS6), F7 (figS6), F12 (figS6), F44 (figS6), F148 (figS6), F163 (figS6), F235 (figS6), F242 (figS6), F273 (figS6), F293 (figS6), F336 (figS6), F379 (figS6),

FU2-57 (figS9), FU4-54 (figS9), FU-40 (figS9), FU7-15 (figS9), FU12-19 (figS9), FU44-18 (figS9), FU99-31 (figS9), FU99-91 (figS9), FU163-109 (figS9), FU210-12 (figS9), FU223-43 (figS9), FU235-66 (figS9), FU273-23 (figS9), FU27377 (figS9), FU300-24 (figS9), FU336-32 (figS9), FU428-8 (figS9), FU426-36 (figS9) —appear ambiguous and may be subject to interpretation. Please, include additional data that could help interpretation, like real-space correlation coefficient (RSCC) and B factors for ligand atoms only, 2Fo-Fc maps ....

→ We thank the reviewer for this careful assessment. We agree that the identification of weakly bound fragments can be ambiguous and that visual inspection alone is insufficient in some cases. To reduce the risk of false positives, all additional hits identified at lower sigma cutoffs were evaluated using multiple criteria, including Fo–Fc and 2Fo–Fc maps, refined ligand B-factors, and comparison of B-factors between the ligand and surrounding residues.

While some of these weak "potential" hits remain borderline, we believe they still provide helpful starting points for fragment optimization. To improve transparency and support interpretation, we have added 2Fo–Fc maps and RSCC values for these fragments in Fig. S7 and S10, as well as 2Fo–Fc maps in Fig. 3. The refined B-factors for individual ligands are now included in Tables S2 and S3.

- The reported CC1/2 value for the outer resolution shell in PDB entry 7IOY is 0.98 (0.09) (Table, page 33). It is unclear whether the value of 0.09 represents the true data cut-off, as it appears unusually low. Were the resulting ligand models chemically plausible and well-refined (e.g., stable B-factors, satisfactory real-space correlation coefficients)? The manuscript should more robustly substantiate the validity of hits identified under these less stringent map thresholds to strengthen confidence in the reported findings See also point above.

→ We thank the reviewer for this valuable comment. All datasets were processed using the same workflow in autoPROC with the default STARANISO settings. No manual adjustments were made to the resolution or CC1/2 cut-off. In STARANISO, anisotropic resolution limits are determined automatically based primarily on ⟨I/σ(I)⟩ rather than a fixed CC1/2 threshold, and no explicit CC1/2 cut-off is defined by default. Therefore, the reported value (0.09) reflects the statistics of the outermost shell but not a manually applied threshold. The resulting ligand models were chemically reasonable and well-refined. We added 2Fo-Fc maps and RSCC to Fig. S7 and S10 and B factors in Table S2 and S3 to support the identification of the hits.

- It would be helpful to present representative refinement statistics (R-factors, Ligand RSCC …) for the full fragment screening dataset, either in Figure S5 or as a separate figure.

→ The RSCC has been added to Fig S7 and S10 for comparison. Furthermore, the B-factors for individual fragments have been recalculated and are included in Tables S2 and S3.
→ the previous Fig S6 and S9, now renumbered to Fig S7 and Fig S10, respectively.

Other Comments
- In the abstract, the phrase "the identification of a novel intracellular ligand-binding site in a GPCR …" could be moderated as the physiological role of this site and its relevance for drug design are yet to be established. For example " numerous ligands were found at a so far uncharacterized intracellular pocket…"

→ Thank you for the reviewer's helpful suggestion. In response, we have revised the sentence to a more moderate formulation: 'Moreover, the detection of ligands at a previously uncharacterized intracellular pocket in a GPCR highlights the potential of this strategy to accelerate the discovery of therapeutically relevant compounds for challenging drug targets.'

- The hits for the well-established extracellular binding site have not been pursued ? Why ? It would have seem logical to focus follow-up efforts on that site.

→ We appreciate the reviewer's comment and fully agree that the extracellular binding site is a logical and well-established target. However, this site has been extensively characterized in many studies, and the three hits we identified correspond to core scaffolds already known from optimized ligands at this site. Considering the overlap with known chemistry, as well as practical constraints of time, resources, and the specific focus of our study, we chose not to pursue further work on this region. Instead, we focused on the less explored intracellular binding site, which we believe offers novel mechanistic and pharmacological insights.

- LCP-CFS pipeline is undoubtedly effective. However, the negative comments concerning the previous work by the Marquez group at EMBL (Healey et al. ref 29) " lack of reproducibility of vapour diffusion" and "limited access" don't seem justified. Vapour diffusion plates are used in the present work as well and the CrystalDirect technology is available both commercially and through user facilities at two synchrotrons sites. Healey et al. did also demonstrate soaking of LCP crystals, which is not mentioned here.

→ We agree with the reviewer. The negative comments have been removed, and the paragraph is rephrased as
"More recently, Healey's group [29] introduced an automated platform using CrystalDirect (CD) plates, and applied it to soaking of LCP crystals, including direct LCP soaking of targets such as ACER3 and ADIPOR2. In addition, this platform is integrated with a web-based Crystallographic Information Management System (CRIMS) for crystal growth, harvesting and data collection."

- The manuscript claims 10% DMSO "yielded the best electron density map quality for the ligand." How was "best quality" determined? This subjective assessment should be supported by quantitative data. Please provide metrics such as the refined B-factor, occupancy, and/or real-space correlation coefficient (RSCC) for the ligand under the different conditions tested.

→ We appreciate the reviewer's comment. The quantitative metrics, including refined B-factors, RSCC values, and ligand occupancy, are provided in Fig. S4. An additional sentence has been added to the manuscript for clarification: "These data show that the soaking conditions of 3 h with 5 mM or 10 mM ligand (corresponding to 5% and 10% DMSO) yield comparable statistics. Given the low affinity of the fragments, we selected 10 mM ligand in 10% DMSO as the optimal condition for fragment soaking."

Reviewer #3 (Remarks to the Author):

The manuscript presents a pipeline for fragment screening of GPCR crystals grown in LCP. The pipeline was applied to a thermostabilized adenosine A_{2A} receptor to screen 568 fragments, identifying 23 initial hits. Further optimization of these hits led to the design of 109 follow-up compounds, 56 of which yielded crystal structures. This is an impressive work by a team from the Paul-Scherrer Institute in collaboration with the company LeadXpro. Fragment screening has been widely used with crystals of soluble proteins, however, its application here to membrane proteins crystallized in LCP is novel. Importantly, this screening identified a novel allosteric site at the intracellular side of the receptor and discovered several compounds binding to this site. The caveat is that the receptor had to be heavily thermostabilized and an orthosteric ligand was required for crystallization, limiting the applicability for screening fragments binding in the orthosteric site by this method. Nevertheless, the work should attract attention of structural biologists and medicinal

chemists, however, the authors should address specific comments below before this manuscript can be recommended for publication.

Lines 84-88. Please describe the abbreviation $tsA_{2A}R$ and explain that the receptor was crystallized in the presence of 5 mM of theophylline in the first paragraph of the Results section.

→ We thank the reviewer for this comment. A description for the $tsA_{2A}R$ has been added to the line 84 "(human adenosine A_{2A} receptor with A277S revert mutation, $A_{2A}R$-StaR2-bRIL-A277S, hereafter referred to as $tsA_{2A}R$, crystals were grown in the present of theophylline, details in method section)"

Line 86. The main text mentions that crystallization buffer without theophylline was added to each well, however, in the Methods (Line 430) the crystallization buffer added to each well contains theophylline. Which one is correct?

→ Thank the reviewer for pointing out this. The method (line 430) has been corrected. We removed "5 mM theophyline" in the description and change the "crystallization solution" to "reservoir solution".

Lines 89-108. According to Figure S2, the LCP transforms into a sponge phase after about 3 h of incubation in the SwissCI-3-lens plate even without ligand and DMSO added, meaning that the phase change occurs mostly due to dehydration rather than DMSO or ligand addition. Although $tsA_{2A}R$ crystals apparently survive the phase change, such change in conditions may be incompatible with crystals of other membrane proteins. This would be an additional limitation of this method.

→ We are aware that the phase transition could potentially have negative effects on certain LCP-grown crystals. For this reason, we optimized the DMSO concentration and soaking time to minimize adverse impacts during soaking. In the case of tsA_{2A} receptor crystals, we observed that the LCP spontaneously transforms into the sponge phase after several hours of incubation, even without DMSO or ligand addition, yet the diffraction quality remains unaffected. This observation is consistent with previous reports that tsA_{2A} crystals can tolerate such transitions.

Importantly, the sponge phase, characterized by enlarged aqueous channels, may actually facilitate fragment diffusion and thus improve soaking efficiency in tsA_{2A} crystals. Nevertheless, we acknowledge that for other membrane proteins, especially those sensitive to dehydration or phase instability, such transitions could indeed pose limitations. To minimize this risk, we recommend controlling humidity and reducing the transfer time when moving LCP from the syringe to the plate. Alternatively, using higher fragment concentrations can improve diffusion efficiency, allowing soaking to be completed before the phase transition occurs.

Lines 89-108. The ligand soaking method was developed using the high affinity antagonist ZM241385 with Ki~1 nM. Of course, none of the screened fragments is expected to bind with such high potency. Therefore, the soaking time and concentrations optimized for ZM241385 are unlikely to be optimal for most low potency fragments. It would be helpful if the authors re-assess the soaking time vs concentration using a low affinity antagonist with Ki>1 μM.

→ We thank the reviewer for this helpful comment. Our initial objective was to confirm that the LCP-CFS soaking method functioned reliably, so we used the high-affinity antagonist ZM241385 to validate ligand uptake clearly and to establish basic soaking parameters. This

initial test also successfully produced fragment hits, indicating that the optimized conditions are suitable even for weak binders.

We agree that a low-affinity antagonist (Ki > 1 µM) would better represent fragment behavior. However, due to time constraints and the limited availability of suitable weak binders during the experimental phase, we proceeded with ZM241385 for method development. We will consider further optimization with a representative weak binder in future work.

Lines 257-269. Please provide complete results for the GCI assays, including raw curves, association (Ka) and dissociation (Kd) rates and Rmax values for the screened 109 compounds, as well as the dose-rate results and measured affinities for the 19 compounds identified as hits in both X-ray and GCI assays. Which His tag and at which terminus was used for capturing purified receptors on a PCP-NTA chip for the GCI assays? Additionally, it would be important to evaluate the allosteric effect of the most potent compounds in cell signaling assays. Do they act as negative allosteric modulators?

→ Please see our responses to Reviewer 1 above.
→ The target proteins used contain a c-terminal 10x Histidine tag for capturing on the Ni-NTA PCP sensor surface. This has been clarified in the GCI method section.
→ Biological signaling assays for testing potential cellular effects of the identified compounds have not yet been conducted.

Line 259. Does the wtA_{2A}R contain bRIL fusion? This should be clarified, and perhaps it would be more appropriate to use wtA_{2A}R-bRIL abbreviation instead of wtA_{2A}R. The tsGPR17 construct should also be described.

→ We thank the reviewer for this comment. Both wtA_{2A}R and tsA_{2A}R constructs used in this study contain a bRIL fusion; this has now been clarified in the manuscript. The GPR17 construct used as a negative control on GCI corresponds to residues 1-342 of human GPR17. A HA signal sequence and FLAG peptide were inserted at the N-terminus of the protein. The C-terminus was truncated at residue 342 and replaced with a 3C cleavage site, followed by a 10x His, TEV cleavage site and enhanced green fluorescence protein (eGFP) to facilitate purification. To enhance protein's thermal-stability, 7 mutations (D69V, C108Y, A155L, S315V, S107L, F158Y and T230L) were introduced to the wild type sequence. This has also been added to the corresponding method part.

Line 428. SwissCI-3-lens plates are offered in three different materials: PS, UVP, and UVXPO. Each of these polymers display different birefringent background under cross-polarizers. Please, specify, which exactly plates were used.

→ We thank the reviewer for pointing this out. All experiments reported in this study were performed using SwissCI-3-lens UVXPO plates. This plate type exhibits low birefringence and is therefore well suited for observing LCP crystals under cross-polarized light. This information has now been added to the Methods section.

Line 446. The authors mention that “fragment-soaked crystals were harvested after either 3 or 21 hours”. This is a large difference. The actual soaking time for each fragment should be indicated in Table S4.

→ We thank the reviewer for pointing this out. All samples listed in Table S4 correspond to tsA_{2A} LCP crystals soaked with fragments for 3 hours. We have added a note at the end of Table S4 to clarify the soaking time. This duration was chosen because the fragment binding efficiency, as indicated by the electron density maps and quantitative statistics (Fig. S4), reached sufficient occupancy within 3 hours.

Lines 462-474. Some X-ray data were collected at SLS and some at ESRF. Please, indicate explicitly which datasets were collected at each of the synchrotron source.

→ We thank the reviewer for pointing this out. The initial screening of 568 fragments and the DMSO optimization with tsA$_{2A}$ LCP crystals were performed at the SLS, while the subsequent soaking (follow-up) of 109 fragments with tsA$_{2A}$ LCP crystals was carried out at ESRF. We have added this information to the Methods section as well as to Tables S1, S2, and S3.

Line 476. STARANISO was used to apply anisotropic truncation to X-ray data. Please indicate resolution limits applied by STARANISO for each dataset in Tables S1-S3.

→ We thank the reviewer for this comment. The datasets listed in Table S1 were processed using XDS, whereas all fragment datasets in Tables S2 and S3 were processed using autoPROC with the STARANISO option. This distinction and the corresponding anisotropic resolution limits determined by STARANISO have now been clarified and included in Tables S1–S3.

Lines 480-481. Phenix, BUSTER, and REFMAC were used for refinement. Please, include the versions of the refinement programs and indicate the reason why different programs were used. It is difficult to compare statistics when different datasets are refined by different programs.

→ We thank the reviewer for pointing this out. REFMAC (5.8.0267) was used during PanDDA analysis, and BUSTER (2.10.4) was applied to refine ambiguous hits. However, all final fragment hits were ultimately refined using Phenix (1.20_4459) to ensure consistency in the reported statistics. We have added this information to the Methods section for clarification.

Tables S1-S3. The overall completeness of the fragment-soaked datasets ranges from 42% (FU35-2) to 88.4% (F99) with majority around 70%. The completeness of the ZM241385-soaked data in Table S1 is much higher (>99%). What is the reason for such low completeness of the fragment-soaked data, and how confident the authors are in the Fo-Fc densities for such datasets? Do the Fo-Fc densities shown in Figures 3, S6, S9 use missing Fo filled with Fc? If yes, how do the densities change if filling is not used?

→ We thank the reviewer for the comment. The dataset for ZM241385 was processed using XDS, as this ligand is a well-characterized high-affinity A$_{2A}$ binder and the dataset could be reliably handled with a straightforward processing approach. All fragment datasets, however, were processed with autoPROC with the default STARANISO option to ensure that all usable reflections were retained.
All the Fo-Fc maps shown (green mesh) were generated by DIMPLE after removing the ligand. Missing reflections in the input data were not filled with Fc; the maps therefore reflect only the experimentally observed structure factors. We note that the fragment densities remain clearly visible even with this conservative calculation.

Tables S1-S3. The Rmeas values are relatively high, exceeding 30% for many datasets and even 70% for some. It is hard to believe that such data can produce reliable density for the fragments. Wilson-B is also suspicious in several datasets, such as 9.48 (FU132-4) and 11.08 (FU35-47).

→We thank the reviewer for raising this important point regarding the data quality. We acknowledge that the Rmeas values of some datasets appear relatively high. This is mainly due to the weak and anisotropic diffraction commonly observed in GPCR–LCP crystals,

especially for small-fragment soaks. Notably, despite the elevated Rmeas, the electron density maps for these datasets remain interpretable, and the fragment-binding features are clearly supported by both 2Fo−Fc and Fo−Fc maps. We also note that all datasets were processed using autoPROC with the default STARANISO option, and no processing parameters were manually restricted, which may explain the higher global statistics.

We agree that the Wilson B-values of FU_{132}-4 and FU_{35}-47 (9.48 and 11.08 Å^2) are unusually low. Diffraction data were processed using autoPROC with anisotropic correction applied by STARANISO. Anisotropic truncation alters the distribution of reflection intensities by selectively retaining high-resolution data along stronger reciprocal-space directions while removing weak reflections in others. As a consequence, the assumptions underlying the Wilson plot, which require isotropic and uniformly sampled data, are no longer strictly valid. Wilson B-factors calculated after STARANISO correction are therefore systematically underestimated.

However, both crystals contain strong and well-defined electron density (Fig. S10), and the refined atomic B-factors are consistent with well-ordered ligand–protein complexes, indicating that the underlying diffraction data are not problematic.

For completeness, we have included the refined B-factors of individual fragments in Tables S2 and S3, and the corresponding RSCC values are provided in Figs. S7 and S10.

Table S4. Please include molecular weights for all the fragments. Also please provide a list of all 568 fragments used in this work.

→ We thank the reviewer for this suggestion. The molecular weights of all hit fragments have now been added to Table S4. We would be happy to provide a complete list of all 568 fragments used in this work upon request.

Supplementary Method. The authors describe a pipeline implemented in Python 3.11.6. They should provide the code for it in github.

→ We thank the reviewer for this suggestion. The Python-based pipeline described in the Supplementary Methods is a workflow tailored to our local setup rather than a general software tool. The underlying code is not in a publishable form—it consists of multiple interconnected scripts tied to our in-house infrastructure, vendor APIs requiring private access keys, and interactive third-party software. Preparing it for public release would require substantial redevelopment.

However, the workflow is described in sufficient detail for others to conceptually reproduce the approach, and we note that a more robust and portable version may be developed and published separately in the future.

Reviewer #4 (Remarks to the Author):

I co-reviewed this manuscript with one of the reviewers who provided the listed reports. This is part of the Communications Chemistry initiative to facilitate training in peer review and to provide appropriate recognition for Early Career Researchers who co-review manuscripts.

Point-by-point response revision 2

Reviewer #1 (Remarks to the Author):

The authors have added exemplary original data to illustrate the results from the GCI biosensor analysis. However, even with the additional data, necessary information is still missing. Clearly the authors have a challenge in using it for membrane bound receptors and they correctly state that it is not realistic to expect the estimation of kinetic parameters or affinities. So, this is not what is asked for. Nevertheless, since they state that they have integrated GCI biosensor analysis in their fragment screening pipeline, it is therefore important to understand how this method contributed to identifying and validating the hits. Without describing the method and criteria used for identifying hits and discriminating them from non-hits, this is not possible. This should be seen in the light of GCI biosensor technology being relatively new and the interpretation of the data is difficult. The reader needs to have access to the complete dataset and be guided in interpreting it. If the authors believe that there is relevant information on the procedure (screening criteria and data etc) already available in the literature, it should be referenced so the reader can find it.

→ Based on this reviewer's comment, we feel that there is a misunderstanding. The primary objective of our study was to establish and demonstrate a practical and widely accessible LCP-based fragment screening (LCP-CFS) workflow. While affinity or GCI measurements can serve as useful complementary validation data, they are not essential to the core methodology we present in the current manuscript. The figure 1 and the description in the revised manuscript now have revised for clarify this point.

In fact, our intention was to highlight that LCP-CFS can be effectively performed and yield meaningful structural insights even in the absence of any comprehensive affinity assays. For this reason, we feel that presenting the selected overlapping fragment hits - rather than the entire set of 109 GCI measurements, which would detract from the main focus of our study - is sufficient to support our conclusions. What we nevertheless present in our revised manuscript are the 19 hits that were consistently identified by both GCI analysis and crystallographic screening. Additionally, we included hits that were only detected by GCI (Table S6). These complementary results provide the most relevant validation of our main structural findings.

We have now also added further details describing the GCI biosensor method in the Material and Method section, together with the screening criteria used to identify fragments interacting with the target receptor. We acknowledge that the technology as well as their application for interaction analysis of fragments is relatively new. In addition, as mentioned in the manuscript, performing such experiments on detergent-solubilized membrane proteins leads to additional artefacts in the signals. We have developed criteria based on statistical metrics calculated by the evaluation software as well as on the R_{max} calculated form the fits to filter for higher confidence interaction signals which are still within a certain amplitude range and not showing super-stoichiometry. The statistical parameters used are the calculated error values on the on- and off-rates upon fitting the experimental data to a 1:1 Langmuir interaction

model. The obtained sensor responses are in many cases of low amplitude and distorted by artefacts, and do not provide definite proof of a 1:1 interaction. However, filtering the data with the above-mentioned criteria identifies potential interactors and removes highly over-stoichiometric signals. We would like to clarify that the GCI experiments were primarily performed to provide complementary support for the crystallographic fragment hits.

Importantly, our work is the first to demonstrate that large-scale crystallographic fragment screening can be successfully applied to membrane proteins, including the particularly challenging GPCR class. We are confident that our methodology will pave the way for new opportunities in rational drug development, especially since membrane proteins such as GPCRs represent the largest class of drug targets to date.

Specific points:
• GCI biosensor data is only shown for 5 hits although 19 out of the 56 compounds that yielded crystal structures were stated to be validated by GCI analyses. Data for all these compounds should be shown so it is possible to see that they fulfil criteria used to identify them as hits.

→ We agree with the reviewer. To ensure full transparency and allow independent assessment of the screening criteria, we now provide the GCI biosensor data for all fragments that were identified and validated (19 hits that were identified by both GCI analysis and crystallographic screening, and 12 hits that only detected by GCI) in a separate supplementary file (Table S6). This enables the reader to evaluate whether the applied filtering criteria were fulfilled in each case.

In addition, we have expanded the description of the screening workflow and included representative examples (Fig. S11 and 12) in the main manuscript to guide interpretation of the complete dataset (see page 8 of the revised manuscript).

• Figure S11.
o The criteria used to confirm binding of the hits to the target by the GCI method is described in the Methods section, but a combination of a variety of criteria appears to be considered. The way this is implemented if not shown so the reader can understand why the data shown has been interpreted to represent hits. What about the other 14 hits?

→ As mentioned, we have used the errors calculated by the evaluation software on the rate constants derived from fitting to a 1:1 model as well as the fit R_{max} to filter for higher confidence interaction signals which are still within a certain amplitude range and not showing super-stoichiometry. The full dataset of hits from the X-ray screen which could be confirmed in GCI is provided in a separate file (Table S6).

o The font size for the units on the y-axis is too small to be read. (The units can be explained in the legend.) What was the rationale for setting the confidence to high for signals between 1 and 10 pg/mm²?

→ The axis units have been now labelled more clearly.

→ An approximate theoretical R_{max} can be calculated based on the molecular weight of the receptor, the fragments and the immobilization levels. This reflects the maximum range of amplitudes to be expected for a 1:1 binding event. We have therefore defined thresholds for the signal amplitudes to filter out over-stoichiometric interactions, which are likely to be derived from unspecific binding to the target.

o The dilution series from 200 uM top concentration is not specified.

→ For the waveRAPID method, the exact dilution series cannot be specified, as the analyte concentration is dynamically modulated directly on the sensor based on the injection pulses with increasing pulse-width. Calibration injections with DMSO are used by the software to calibrate and normalize a dynamic concentration function based on refractive index (RI) changes of DMSO as a calibrator being detected as solution bulk RI signal on the sensor surface. We have added the relevant reference for this methodology (Kartal, Önder et al. waveRAPID-A Robust Assay for High-Throughput Kinetic Screens with the Creoptix WAVEsystem. SLAS discovery: advancing life sciences R & D vol. 26,8 (2021))

The data for the two controls with adenosine and NECA that have been included, as requested. But they are not commented at all, so it is not possible to understand in what way these compounds serve as controls. What information is extracted from these control measurements?

→ We thank the reviewer for this comment. We have now clarified the role of these controls in the revised manuscript (see page 8). We include an extended figure S11 showing the interaction signals measured on the apo A_{2A}R in absence of ZM241385. Adenosine and NECA interactions can be detected on apo A_{2A} R and their binding is blocked by ZM241385. Adenosine and NECA were used as control to confirm apo A_{2A} R activity on the sensor and orthosteric site blocking by ZM241385. This has now been clarified in the text.

It is not a 1:1 interaction model that is shown in black, but most likely theoretical traces obtained by regression analysis using a 1:1 model.

→ The black traces depict a modelled 1:1 interaction after fitting to the double-referenced experimental data shown in red.

•Figure S12.
The font size in the figures is too small to be legible.
→ We have improved the labelling and description of the plots.

It appears that different KD and Rmax values are estimated in the global fit and the steady-state analysis. This should be commented.

→ We thank the reviewer for pointing this out and we clarified this further. Based on previously mentioned considerations, the estimated kinetic rate constants, K_D and R_{max} values as shown on the plots are not considered precise and no interpretations are drawn from them. We do only deduce evidence for target engagement from the

biosensor data. The steady-state plots are shown as an additional representation of the signal response following a concentration series of the respective fragments. The identified potential interactions are clearly of weak affinities in the two- to three-digit micromolar range.

There is no explanation to how the data extracted from this figure is interpreted.

→ We have added further clarification in the revised manuscript (see page 8). Selected fragments identified as interactors were additionally tested and confirmed with classical multi-cycle kinetic (MCK) dose responses. The obtained signals do not show full saturation even at the maximum concentration of 400 μM tested, which reflects their weak binding affinities.

Reviewer #2 (Remarks to the Author):

The authors have satisfactorily addressed this reviewer's concerns with this version of the MS.

Reviewer #3 (Remarks to the Author):

The authors have addressed most of the critiques and improved the manuscript, however, several questions remain unresolved. My additional comments are provided below in bold.

Lines 84-88. Please describe the abbreviation tsA2AR and explain that the receptor was crystallized in the presence of 5 mM of theophylline in the first paragraph of the Results section.

→ We thank the reviewer for this comment. A description for the tsA2AR has been added to the line 84 "(human adenosine A2A receptor with A277S revert mutation, A2AR-StaR2-bRIL-A277S, hereafter referred to as tsA2AR, crystals were grown in the present of theophylline, details in method section)"

Please, add thermostabilized – ("human thermostabilized adenosine A2A ...)
→ We have added the "thermostabilized" to the description.

Line 86. The main text mentions that crystallization buffer without theophylline was added to each well, however, in the Methods (Line 430) the crystallization buffer added to each well contains theophylline. Which one is correct?

→ Thank the reviewer for pointing out this. The method (line 430) has been corrected. We removed "5 mM theophyline" in the description and change the "crystallization solution" to "reservoir solution".

Ok. Theophylline is misspelled.
→ Thank the reviewer for pointing out this. It has been corrected.

Lines 89-108. According to Figure S2, the LCP transforms into a sponge phase after about 3 h of

incubation in the SwissCI-3-lens plate even without ligand and DMSO added, meaning that the phase change occurs mostly due to dehydration rather than DMSO or ligand addition. Although tsA2AR crystals apparently survive the phase change, such change in conditions may be incompatible with crystals of other membrane proteins. This would be an additional limitation of this method.

→ We are aware that the phase transition could potentially have negative effects on certain LCP-grown crystals. For this reason, we optimized the DMSO concentration and soaking time to minimize adverse impacts during soaking. In the case of tsA2A receptor crystals, we observed that the LCP spontaneously transforms into the sponge phase after several hours of incubation, even without DMSO or ligand addition, yet the diffraction quality remains unaffected. This observation is consistent with previous reports that tsA2A crystals can tolerate such transitions. Importantly, the sponge phase, characterized by enlarged aqueous channels, may actually facilitate fragment diffusion and thus improve soaking efficiency in tsA2A crystals. Nevertheless, we acknowledge that for other membrane proteins, especially those sensitive to dehydration or phase instability, such transitions could indeed pose limitations. To minimize this risk, we recommend controlling humidity and reducing the transfer time when moving LCP from the syringe to the plate. Alternatively, using higher fragment concentrations can improve diffusion efficiency, allowing soaking to be completed before the phase transition occurs.

Please, add these recommendations to the manuscript.
→ This has been added to the discussion. Please see page 9.

Lines 89-108. The ligand soaking method was developed using the high affinity antagonist ZM241385 with Ki~1 nM. Of course, none of the screened fragments is expected to bind with such high potency. Therefore, the soaking time and concentrations optimized for ZM241385 are unlikely to be optimal for most low potency fragments. It would be helpful if the authors re-assess the soaking time vs concentration using a low affinity antagonist with Ki>1 μM.

→ We thank the reviewer for this helpful comment. Our initial objective was to confirm that the LCP-CFS soaking method functioned reliably, so we used the high-affinity antagonist ZM241385 to validate ligand uptake clearly and to establish basic soaking parameters. This initial test also successfully produced fragment hits, indicating that the optimized conditions are suitable even for weak binders.
We agree that a low-affinity antagonist (Ki > 1 μM) would better represent fragment behavior. However, due to time constraints and the limited availability of suitable weak binders during the experimental phase, we proceeded with ZM241385 for method development. We will consider further optimization with a representative weak binder in future work.

Please, acknowledge this limitation in the manuscript.
→ This has been added to the discussion. Please see page 9.

Lines 257-269. Please provide complete results for the GCI assays, including raw curves, association (Ka) and dissociation (Kd) rates and Rmax values for the screened 109 compounds, as well as the dose-rate results and measured affinities for the 19 compounds identified as hits

in both X-ray and GCI assays. Which His tag and at which terminus was used for capturing purified receptors on a PCP-NTA chip for the GCI assays? Additionally, it would be important to evaluate the allosteric effect of the most potent compounds in cell signaling assays. Do they act as negative allosteric modulators?

→ *Please see our responses to Reviewer 1 above.*
→ *The target proteins used contain a c-terminal 10x Histidine tag for capturing on the Ni-NTA PCP sensor surface. This has been clarified in the GCI method section.*
→ *Biological signaling assays for testing potential cellular effects of the identified compounds have not yet been conducted.*

C-terminal 10x His-tag is described for GPR17 but no description is given for tsA$_{2A}$ and wtA$_{2A}$.
→ The detail description has been added to the corresponding part in the "Material and Method".

Line 259. Does the wtA2AR contain bRIL fusion? This should be clarified, and perhaps it would be more appropriate to use wtA2AR-bRIL abbreviation instead of wtA2AR. The tsGPR17 construct should also be described.

→ *We thank the reviewer for this comment. Both wtA2AR and tsA2AR constructs used in this study contain a bRIL fusion; this has now been clarified in the manuscript. The GPR17 construct used as a negative control on GCI corresponds to residues 1-342 of human GPR17. A HA signal sequence and FLAG peptide were inserted at the N-terminus of the protein. The C-terminus was truncated at residue 342 and replaced with a 3C cleavage site, followed by a 10x His, TEV cleavage site and enhanced green fluorescence protein (eGFP) to facilitate purification. To enhance protein's thermal-stability, 7 mutations (D69V, C108Y, A155L, S315V, S107L, F158Y and T230L) were introduced to the wild type sequence.*
This has also been added to the corresponding method part.

ok

Line 428. SwissCI-3-lens plates are offered in three different materials: PS, UVP, and UVXPO. Each of these polymers display different birefringent background under cross-polarizers. Please, specify, which exactly plates were used.

→ *We thank the reviewer for pointing this out. All experiments reported in this study were performed using SwissCI-3-lens UVXPO plates. This plate type exhibits low birefringence and is therefore well suited for observing LCP crystals under cross-polarized light. This information has now been added to the Methods section.*

ok

Line 446. The authors mention that "fragment-soaked crystals were harvested after either 3 or 21 hours". This is a large difference. The actual soaking time for each fragment should be

indicated in Table S4.

→ *We thank the reviewer for pointing this out. All samples listed in Table S4 correspond to tsA2A LCP crystals soaked with fragments for 3 hours. We have added a note at the end of Table S4 to clarify the soaking time. This duration was chosen because the fragment binding efficiency, as indicated by the electron density maps and quantitative statistics (Fig. S4), reached sufficient occupancy within 3 hours.*

Sorry, I do not see a note about soaking time in Table S4?

→ We thank the reviewer for the comment. Upon rechecking the file, we confirmed that Note 5 was present in the Excel sheet; however, due to merged file it was not visible in the print view. Now we have carefully re-checked the supplementary file to ensure that this note is clearly visible and correctly formatted.

Lines 462-474. Some X-ray data were collected at SLS and some at ESRF. Please, indicate explicitly which datasets were collected at each of the synchrotron source.

→ *We thank the reviewer for pointing this out. The initial screening of 568 fragments and the DMSO optimization with tsA2A LCP crystals were performed at the SLS, while the subsequent soaking (follow-up) of 109 fragments with tsA2A LCP crystals was carried out at ESRF. We have added this information to the Methods section as well as to Tables S1, S2, and S3.*

ok

Line 476. STARANISO was used to apply anisotropic truncation to X-ray data. Please indicate resolution limits applied by STARANISO for each dataset in Tables S1-S3.

→ *We thank the reviewer for this comment. The datasets listed in Table S1 were processed using XDS, whereas all fragment datasets in Tables S2 and S3 were processed using autoPROC with the STARANISO option. This distinction and the corresponding anisotropic resolution limits determined by STARANISO have now been clarified and included in Tables S1–S3.*

I do not see anisotropic resolution limits in Tables S1-S3. In table S3, the resolution range for the highest resolution shell and the total resolution range are switched (the highest resolution shell should be in parentheses).

→ We thank the reviewer for pointing out this mistake. The table has been corrected so that the highest-resolution shell is now correctly shown in parentheses. Anisotropic resolution limits (based on the "Diffraction limits & principal axes of ellipsoid fitted to diffraction cut-off" from the autoProc log file) have been added to Table S3 (previously Table S2) and Table S4 (previously Table S3). The data shown in Table S2 (previously Table S1) were processed using XDS, which does not provide anisotropic resolution limits; therefore, no anisotropic resolution limits are reported for this dataset.

Lines 480-481. Phenix, BUSTER, and REFMAC were used for refinement. Please, include the versions of the refinement programs and indicate the reason why different programs were used.

It is difficult to compare statistics when different datasets are refined by different programs.

→ *We thank the reviewer for pointing this out. REFMAC (5.8.0267) was used during PanDDA analysis, and BUSTER (2.10.4) was applied to refine ambiguous hits. However, all final fragment hits were ultimately refined using Phenix (1.20_4459) to ensure consistency in the reported statistics. We have added this information to the Methods section for clarification.*

ok

Tables S1-S3. The overall completeness of the fragment-soaked datasets ranges from 42% (FU35-2) to 88.4% (F99) with majority around 70%. The completeness of the ZM241385-soaked data in Table S1 is much higher (>99%). What is the reason for such low completeness of the fragment-soaked data, and how confident the authors are in the Fo-Fc densities for such datasets? Do the Fo-Fc densities shown in Figures 3, S6, S9 use missing Fo filled with Fc? If yes, how do the densities change if filling is not used?

→ *We thank the reviewer for the comment. The dataset for ZM241385 was processed using XDS, as this ligand is a well-characterized high-affinity A2A binder and the dataset could be reliably handled with a straightforward processing approach. All fragment datasets, however, were processed with autoPROC with the default STARANISO option to ensure that all usable reflections were retained.*
All the Fo-Fc maps shown (green mesh) were generated by DIMPLE after removing the ligand. Missing reflections in the input data were not filled with Fc; the maps therefore reflect only the experimentally observed structure factors. We note that the fragment densities remain clearly visible even with this conservative calculation.

Please, include the information about the Fo-Fc maps generation and Fc filling in Methods.
→ This has been added to the corresponding part in the "Material and Method".

Tables S1-S3. The Rmeas values are relatively high, exceeding 30% for many datasets and even 70% for some. It is hard to believe that such data can produce reliable density for the fragments. Wilson-B is also suspicious in several datasets, such as 9.48 (FU132-4) and 11.08 (FU35-47).

→*We thank the reviewer for raising this important point regarding the data quality. We acknowledge that the Rmeas values of some datasets appear relatively high. This is mainly due to the weak and anisotropic diffraction commonly observed in GPCR–LCP crystals, especially for small-fragment soaks. Notably, despite the elevated Rmeas, the electron density maps for these datasets remain interpretable, and the fragment-binding features are clearly supported by both 2Fo−Fc and Fo−Fc maps. We also note that all datasets were processed using autoPROC with the default STARANISO option, and no processing parameters were manually restricted, which may explain the higher global statistics.*
We agree that the Wilson B-values of FU132-4 and FU35-47 (9.48 and 11.08 Å^2) are unusually low. Diffraction data were processed using autoPROC with anisotropic correction applied by STARANISO. Anisotropic truncation alters the distribution of reflection intensities by selectively

retaining high-resolution data along stronger reciprocal-space directions while removing weak reflections in others. As a consequence, the assumptions underlying the Wilson plot, which require isotropic and uniformly sampled data, are no longer strictly valid. Wilson B-factors calculated after STARANISO correction are therefore systematically underestimated.
However, both crystals contain strong and well-defined electron density (Fig. S10), and the refined atomic B-factors are consistent with well-ordered ligand–protein complexes, indicating that the underlying diffraction data are not problematic.
For completeness, we have included the refined B-factors of individual fragments in Tables S2 and S3, and the corresponding RSCC values are provided in Figs. S7 and S10.

ok

Table S4. Please include molecular weights for all the fragments. Also please provide a list of all 568 fragments used in this work.

→ We thank the reviewer for this suggestion. The molecular weights of all hit fragments have now been added to Table S4. We would be happy to provide a complete list of all 568 fragments used in this work upon request.

A complete list could be easily included as a supplemental Excel file.
→ A complete list of the smiles of 568 fragments is included in Table S1.

Supplementary Method. The authors describe a pipeline implemented in Python 3.11.6. They should provide the code for it in github.

→ We thank the reviewer for this suggestion. The Python-based pipeline described in the Supplementary Methods is a workflow tailored to our local setup rather than a general software tool. The underlying code is not in a publishable form—it consists of multiple interconnected scripts tied to our in-house infrastructure, vendor APIs requiring private access keys, and interactive third-party software. Preparing it for public release would require substantial redevelopment.
However, the workflow is described in sufficient detail for others to conceptually reproduce the approach, and we note that a more robust and portable version may be developed and published separately in the future.

This is unfortunate, as this part of the workflow could not be exactly reproduced by other researchers.
→ We thank the reviewer for emphasizing the importance of reproducibility. The Python-based pipeline described in the Supplementary Methods is a workflow tailored to our local computational environment rather than a standalone software package. The current implementation consists of interconnected scripts integrated with in-house infrastructure, restricted-access vendor APIs, and third-party software components, and is therefore not portable in its present form.

Nevertheless, the computational procedures, parameter settings, and decision criteria are described in sufficient detail to allow independent researchers to reproduce the analytical strategy, even if the exact internal implementation differs.

Point-by-point response revision 3

Reviewer #1 (Remarks to the Author):

The revised manuscript has addressed the concerns I had with the last version of the manuscript. There are a few minor points that remain:

1. What is meant by “fully bound” and “partially bound” fragments?

→ We thank the reviewer for pointing this out. We agree that the terms “fully bound” and “partially bound” were imprecise. Accordingly, we have revised the text on Page 2 and 4 from “**3 fully and 17 weak or partially bound fragments**” to “**20 fragments**”, and also on Page 6 from “**Most of the initial fragment hits and partial binding events occupy**…” to “**Most of the 20 fragment hits occupy…**” to avoid ambiguity.

We also revised the sentence on page 4,

“**Through this combined approach, our initial inspection of the electron density maps revealed 6 unambiguously bound fragment hits (Fig. S7a and b) and 17 weak or partially bound hits (Fig. S7c). Three of these fragments were bound in the orthosteric binding site (Fig. S7a), displacing the theophylline that was essential for crystallization. The rest of the 20 fragments bound to a distinct intracellular site (Fig. S7b and c).**”

to

“**Through this combined approach, our initial inspection of the electron density maps revealed 3 fragments bound in the orthosteric binding site (Fig. S7a), displacing the theophylline that was essential for crystallization, and 20 fragments bound to a distinct intracellular site (Fig. S7b and c)**.”

In addition, we have included a clarifying sentence on page 5 to better describe how fragment binding was assessed:

“**Initially, upon inspection of the electron density maps of the tsA$_{2A}$R–fragment structures, we contoured the sigma cut-off of the 2*Fo–Fc* and *Fo–Fc* maps at 1.0 and 3.0, respectively, and clearly identified three fragments (F132, F210, and F428) bound at the intracellular site**.”

2. The figures in Figure S11 have axis text and text inside the panels that cannot be read. Pleas make these figures larger or provide the information by some other means.

→ We thank the reviewer for highlighting this issue. The resolution and font size of Figure S11 have been optimized to improve readability.

In addition, we have clarified the axis labels in the figure legend by adding the following description:

“The Y-axis in all GCI waveRAPID plots represents surface mass (pg/mm²), and the X-axis represents time (s).”

3. Page 8: It is stated on row 8 that “ZM241385 blocked the orthosteric site and showed no binding (Fig. S11).” Please explain, it is not logical. If there was a pre-incubation of the surface with ZM241385, it could be possible, but the experiment should be explained, if so and the sentence rephrased.

→ We thank the reviewer for this important comment. To clarify the experimental design, the sentence has been revised to:
“**Adenosine and NECA were used as functional controls in the GCI assay, showing the expected binding response for apo $A_{2A}R$ (Fig. S11). In contrast, no binding was observed when the orthosteric site was blocked with ZM241385 (Fig. S11). Therefore, to specifically probe the intracellular site, ZM241385 was included at saturating concentration in all GCI experiments with the 109 follow-up compounds**.”

Reviewer #3 (Remarks to the Author):

The authors have addressed my comments satisfactory and I'm happy to recommend this manuscript for publication. One small typo in Supplementary Fig. S4, the bottom right section should likely be labeled with 10 µM ZM241385 instead of 5 µM.

→ We thank the reviewer for pointing out this typo. The label in the bottom right section of Supplementary Fig. S4 was incorrect and should read 10 mM ZM241385. This has now been corrected in the revised figure.
